# A novel exchange method to access sulfated molecules

**DOI:** 10.1038/s41598-020-72500-x

**Published:** 2020-10-06

**Authors:** Jaber A. Alshehri, Anna Mary Benedetti, Alan M. Jones

**Affiliations:** School of Pharmacy, University of Birmingham, Edgbaston, B15 2TT UK

**Keywords:** Organic chemistry, Chemical synthesis

## Abstract

Organosulfates and sulfamates are important classes of bioactive molecules but due to their polar nature, they are both difficult to prepare and purify. We report an operationally simple, double ion-exchange method to access organosulfates and sulfamates. Inspired by the novel sulfating reagent, TriButylSulfoAmmonium Betaine (TBSAB), we developed a 3-step procedure using tributylamine as the novel solubilising partner coupled to commercially available sulfating agents. Hence, in response to an increasing demand for complementary methods to synthesise organosulfates, we developed an alternative sulfation route based on an inexpensive, molecularly efficient and solubilising cation exchanging method using off-the-shelf reagents. The disclosed method is amenable to a range of differentially substituted benzyl alcohols, benzylamines and aniline and can also be performed at low temperature for sensitive substrates in good to excellent isolated yield.

## Introduction

Organosulfates and sulfamates contain polar functional groups that are important for the study of molecular interactions in the life sciences, such as: neurodegeneration^1^; plant biology^2^; neural stem cells^3^; heparan binding^4^; and viral infection^5^. Recent total syntheses including 11-saxitoxinethanoic acid^6^, various saccharide assemblies^7–10^, and seminolipid^11^ have all relied on the incorporation of a highly polar organosulfate motif. Importantly, the first in class organosulfate containing antibiotic, Avibactam^12^, has led to the discovery of other novel β-lactamase inhibitors^13,14^. Despite the importance of the sulfate group, there remain difficulties with the ease of their synthesis to enable further biological study.

Our own interest in developing sulfated molecules resulted from a medicinal chemistry challenge to reliably synthesise sulfated glycomimetics^15–18^. We recently reported the development of an all-in-one sulfating reagent, Bu_3_NSO_3_ (TBSAB)^19,20^.

To accelerate the development of complementary methods to prepare organosulfates for biological applications, and inspired by the use of a lipophilic solubilising cation, we sought to develop an alternative sulfation protocol using low-cost, commercially available reagents.

**Figure 1:**
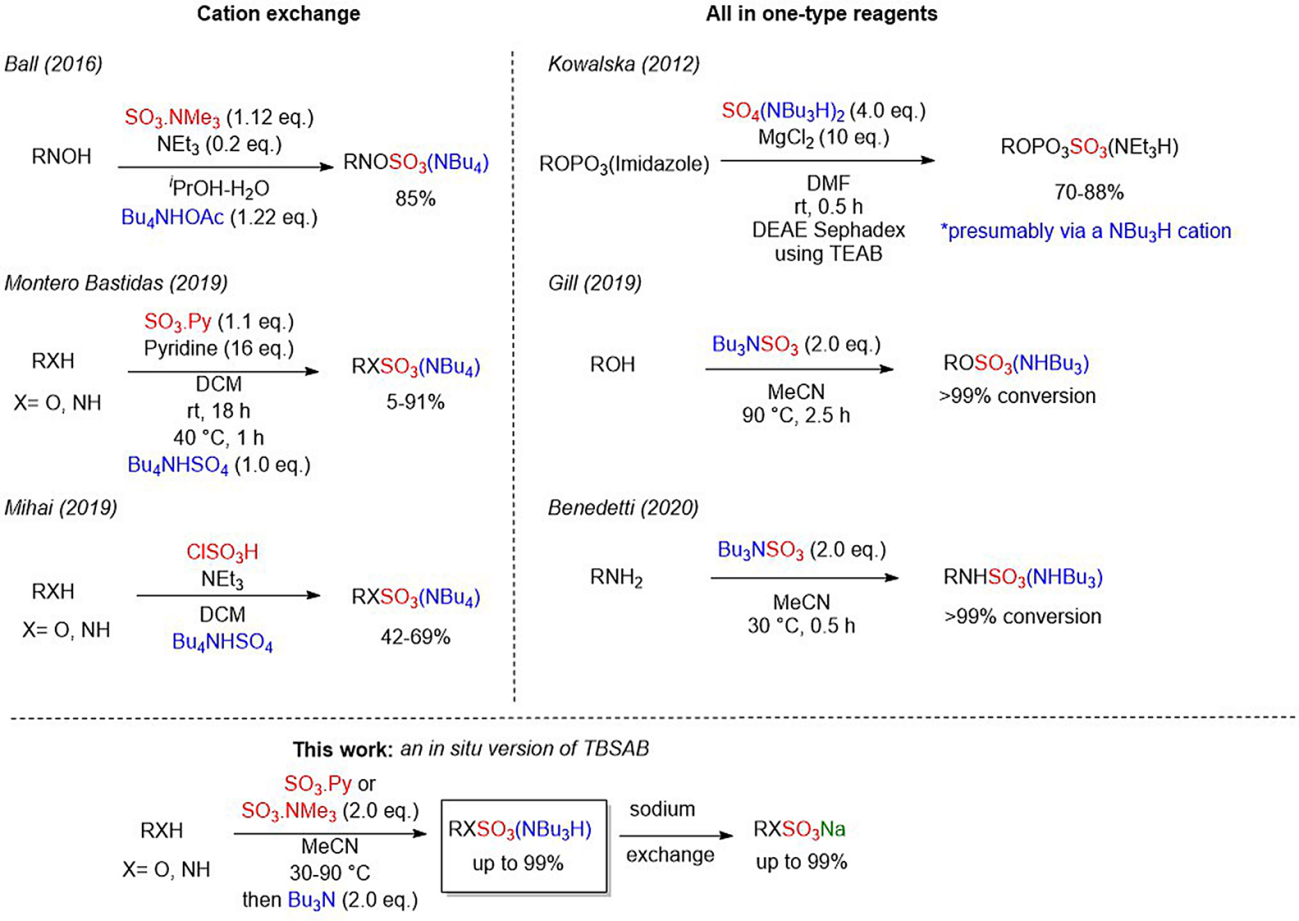
Examples of lipophilic cation-exchange used in sulfation technologies and this work.

To the best of our knowledge, methods to sulfate oxygen, nitrogen, oximes and phosphates that include an organic solubilising cation step remain limited (Fig. 1).

Methods to accomplish a lipophilic cation-exchange of highly polar sulfated molecules include the process route to Avibactam reported by Ball et al.^12^. Recent work by Montero Bastidas et al.^21^, and Mihai et al.^22^ have shown the importance of routes to sulfated molecules with a sterically bulky tetrabutylammonium cation for iridium catalysed *para*-selective C–H borylations. An alternate approach is to design an all-in-one reagent with a sulfating agent combined with a lipophilic counterion, such as our own work^19,20^ and the work of Kowalska et al.^23^.

Herein we explored an alternative method to access organosulfates and sulfamates on a range of alcohols and amines using the inexpensive bulk commodity sulfating chemicals (SO_3_–R, R=Py or NMe_3_) and tributylamine as the lipophilic counterion exchange for the first time.

## Results and discussion

Due to the current need to prepare an all-in-one reagent such as TBSAB (Bu_3_NSO_3_) prior to sulfating an alcohol or amine, we initially investigated whether TBSAB could be prepared from commercial sulfating reagents such as Py-SO_3_ or Me_3_N-SO_3_ and tributylamine. Although ^1^H NMR spectroscopy ruled out the formation of TBSAB in situ (See supporting Information Figure S5), this result led us to consider whether tributylamine could be used to exchange the polar amine sulfation product to a lipophilic tributylammonium cation (*c.f.* the TBSAB reaction product). Therefore, benzyl alcohol was selected as the model scaffold to optimise the formation of a sulfate ester using a one-pot, three-step procedure due to its diagnostic shift in the ^1^H NMR spectrum (Table 1).

**Table 1:** Optimisation and control studies for benzyl alcohol sulfation–lipophilic exchange–sodium exchange.

Entry	Py-SO_3_ (eq)	T (°C)	t (h)	Lipophilic additive	Eq	Sodium exchange	Eq	Conversion to 2 (%)	Yield of 4 (%)
1	1	70	7.0	–	–	–	–	33	–
2	1.2	70	7.0	–	–	–	–	61	–
3	1.5	70	7.0	–	–	–	–	66	–
4	2	70	7.0	–	–	–	–	85	–
5	2	90	3.0	–	–	–	–	99	
6	2	90	3.0	Bu_3_N	2.0	Na-2-ethylhexanoate	2.5	–	57
7	2	90	3.0	Bu_3_N	2.0	Na-2-ethylhexanoate	5.0	–	93
8	2	90	3.0	Bu_4_NI	2.0	Na-2-ethylhexanoate	5.0	–	73
9	2	90	3.0	Bu_4_NOAc	2.0	Na-2-ethylhexanoate	5.0	–	87
10	2	90	3.0	Bu_4_NBr	2.0	Na-2-ethylhexanoate	5.0	–	52
11	2	90	3.0	Bu_3_N	2.0	NaI	2.5	–	66
12	2	90	3.0	Bu_3_N	2.0	NaI	5.0	–	79
13	2	90	3.0	Bu_4_NI	2.0	NaI	5.0	–	83
14	2	90	3.0	Bu_4_NOAc	2.0	NaI	5.0	–	80
15	2	90	3.0	Bu_4_NBr	2.0	NaI	5.0	–	82

**Figure 2:**
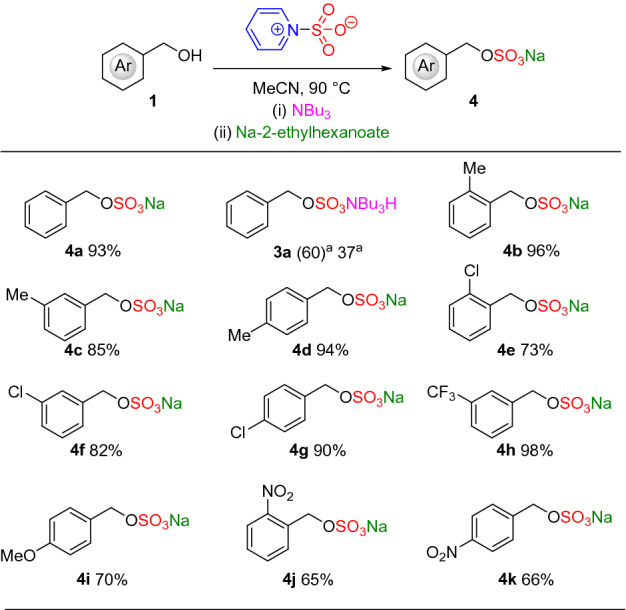
Reaction scope of the 3-step, 1-pot method with benzyl alcohols. Parentheses indicate percentage conversion as measured by ^1^H NMR spectroscopy. ^a^use of Me_3_NSO_3_ instead of PySO_3_.

Entries 1–4 in Table 1 show that conversion to the benzyl sulfate ester pyridinium salt (**2**) was improved with super-stoichiometric equivalents of the pyridine sulfur trioxide complex. Increasing the reaction temperature (entry 5 vs entry 4) resulted in quantitative conversion to the pyridinium salt (**2**). Using the optimal conversion conditions (entry 5) a variety of lipophilic cation exchanging additives were tested to compare tetrabutylammonium salts with tributylamine (entries 6–10). In combination with a sodium salt exchange using sodium 2-ethylhexanoate, it was found that tributylamine afforded the highest isolated yield (94%, entry 7) compared to the tetrabutylammonium salts of iodide, acetate or bromide. The use of an alternative sodium exchange method (NaI, entries 11–15) in tandem with tributylamine or tetrabutylammonium salts was also effective but lower yielding compared to entry 7. With the optimal conditions in hand, we explored the generality of the one-pot, three-step method (Fig. 2).

A variety of benzyl alcohols containing a range of steric and electronic effects were explored (Fig. 2). The method proved tolerant of a wide variety of functionality with isolated yields ranging from 65 to 98%. In comparison with our previously reported all-in-one TBSAB methodology^19^ isolated yields were good but generally lower: **4a** (93% vs 95%), **4e** (73% vs 99%), **4g** (90% vs 94%), **4i** (70% vs 78%), and **4 k** (66% vs 85%).

The rationale for using PySO_3_ over Me_3_NSO_3_ can be seen from the poor conversion observed with **3a** (using Me_3_NSO_3_) versus **4a**. An explanation for this relates to the Lewis basicity of the amine-SO_3_ complex (Py–SO_3_ (*p*K_a_ = 5.23); Me_3_N–SO_3_ (*p*K_a_ = 10.63)). The *sp*^3^ hybridised Lewis base of Me_3_N donates electrons more strongly into the LUMO of SO_3_ forming a hard-hard Lewis adduct with increased stability and decreased reactivity compared to *sp*^2^ hybridised Lewis base seen in Py–SO_3_^24^.

**Scheme 1:**
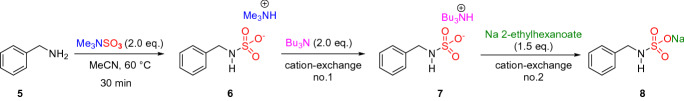
Optimised benzylamine sulfamation conditions.

Next, using the knowledge obtained from the sulfate ester optimisation and our prior work on sulfamates^20^, initial conditions of switching to Me_3_NSO_3_ resulted in quantitative conversion of the benzyl sulfamate (Scheme 1). Slightly lowering the reaction temperature due to the increased nucleophilicity of the *sp*^3^ nitrogen atom, resulted in a quantitative conversion for both the formation of the sulfamate trimethylammonium species and the cation exchanged sulfamate tributylammonium species. It was found that the use of 1.5 eq of sodium 2-ethylhexanoate resulted in a near-quantiative isolated yield (99%).

**Figure 3:**
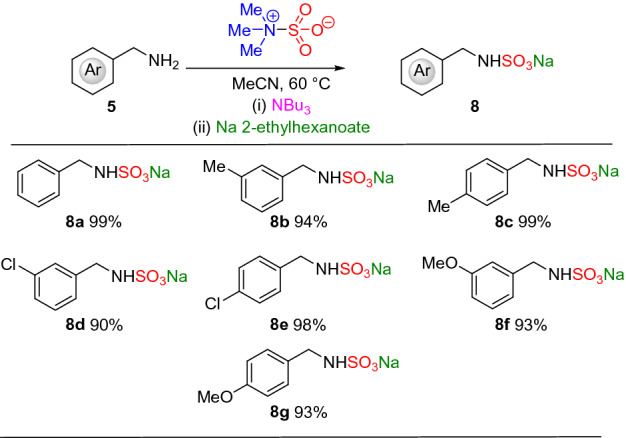
Benzylamine sulfamation reaction scope.

**Figure 4:**
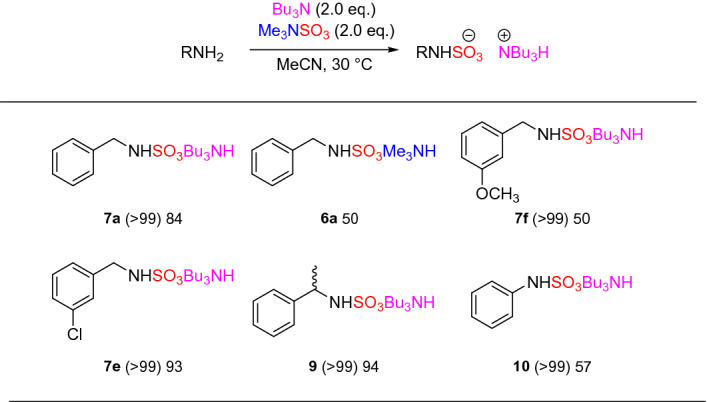
Low-temperature application of the method to a range of nitrogen containing scaffolds. Parentheses indicates % conversion as measured by ^1^H NMR spectroscopy.

With the optimal conditions in hand, we explored the methodology on a selection of benzylamines (Fig. 3). In all cases, an excellent isolated yield, considering the three steps involved, was observed (90–99%) independent of functional group effects. In comparison to our previous methodology, using TBSAB, the following observations were identified: **8a** (99% vs 98%), **8d** (90% vs 97%), **8e** (98% vs 61%), **8f** (93% vs 87%), and **8g** (99% vs 85%). In nearly all cases, the isolated yield for this new route was equivalent or improved for the benzylamines. However, one pertinent disadvantage of the three-step method was the higher reaction temperature, which may not be compatible with more complex molecules. To address this, we next studied a low temperature variant of the new method (Table 2 and Fig. 4).

**Table 2:**
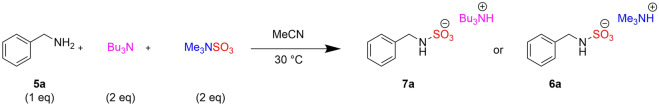

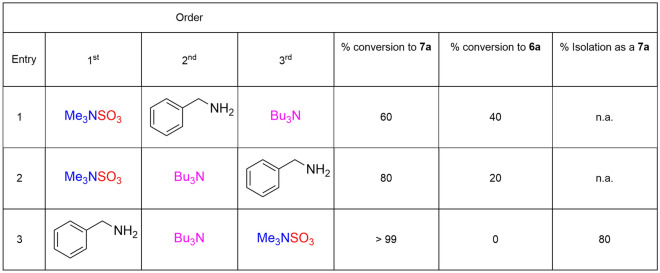
Optimization of a model tributylammonium benzyl sulfamate reaction. Reaction conversions reported as measured by ^1^H NMR spectroscopy.

Due to the rapid kinetics of the reaction between an *sp*^3^ hybridised nitrogen and Me_3_NSO_3_, we explored whether the order of addition at a lower reaction temperature was important with a series of control experiments.

At low temperature (30 °C, entry 1) demonstrated that complete conversion occurs but only 60% results as the tributylammonium cation. Entries 2 and 3 considered whether Bu_3_N should be introduced prior to addition of benzylamine or after addition of benzylamine. The order of addition for rapid, low temperature reaction, became apparent, with a quantitative conversion to the tributylammonium salt (**7a**) with entry 3 and high isolated yield.

With this insight, a series of representative primary, secondary amines and aniline were screened using a low temperature method (Fig. 4). In all cases a high conversion (> 99%) was obtained (Fig. 4) but a lower 50–94% isolated yield of the tributylammonium cation after purification. The intermediacy of the trimethylammonium species, **6a** was confirmed via isolation in a 50% yield.

## Conclusion

In summary, we have developed an alternative operationally straightforward, one-pot, three-step procedure to prepare small molecular weight organosulfates and sulfamates using only low-cost commodity chemicals in generally good to excellent isolated yields. In comparison to the all-in-one TBSAB reagent which can achieve the same or similar transformations in higher yield, the disclosed method does require an additional operational step, but for laboratories without access to methods and equipment to prepare TBSAB, this provides a user-friendly, off-the-shelf approach to preparing sulfated molecules. Improvements in molecular efficiency of lipophilic cation exchange and the avoidance of acidic tetrabutylammonium salts was possible with the new sulfation method.

## Methods

All reactions involving moisture sensitive reagents were carried out using standard Schlenk techniques, in a dry reaction vessel under argon. All solvents used under anhydrous conditions were decanted directly from an SPS dispensary or were stored over 4 Å molecular sieves 24 h prior to use.

Solvents used for workup procedures were of technical grade from Sigma-Aldrich, Honeywell, VWR or Fisher Scientific. Unless stated otherwise, solvents were removed by rotary evaporation under reduced pressure between 30–50 °C. All chemical reagents were used as received unless stated otherwise. Reactions were monitored by TLC analysis on Merck silica gel 60 F254 using UV light (254 nm) and/or potassium permanganate.

^1^H, ^13^C and ^19^F NMR spectra were recorded either on a Bruker AVIII operating at 300 MHz for ^1^H and fitted with a 5 mm BBFO probe or on a Bruker AVANCE NEO operating at 400 MHz for ^1^H fitted with a 5 mm “smart” BBFO probe, respectively^25^. Chemical shift data are reported in parts per million (ppm, δ scale) downfield from tetramethylsilane (TMS: δ 0.0) and referenced internally to the residual proton in the solvent^26^. The deuterated solvents used for NMR analysis were: chloroform (CDCl_3_: δH 7.26, δC 77.2), dimethyl sulfoxide (d_6_-DMSO: δH 2.50, δC 39.5), and deuterium oxide (D2O: δH 4.79). Coupling constants are given in Hertz (Hz)^27^. All individual signals were assigned using 2D NMR spectroscopy (^1^H–^1^H–COSY, ^1^H–^13^C–HSQC, and ^1^H–^13^C–HMBC). The data are presented as follows: chemical shift multiplicity (s = singlet, d = doublet, t = triplet, q = quartet, p = pentet, m = multiple, br = broad and combinations thereof), coupling constant, integration, and assignment^28^. Mass spectra were recorded on a Waters Xevo G2-XS Tof or Synap G2-S mass spectrometer using Zspray, Electro-spray ionization in negative (ESI-) mode. Infrared spectra were recorded on a Perkin Elmer Spectrum 100 FT-IR and a Varian 660-IR FTIR spectrometer using Agilent Resolution Pro, with absorption maxima (νmax) reported in cm^−1^. Optical rotations were measured using a Bellingham and Stanley ADP450 Series Peltier polarimeter at 25 °C using the D line of sodium (589.3 nm) in the indicated concentration and solvent.

### General experimental procedure

*General procedure 1* Synthetic procedure for the preparation of sodium benzyl sulfate ester using sulfur trioxide pyridine complex and tributylamine. A flame dried 100 mL round bottom flask was charged with the appropriate alcohol (1.0 mmol) and pyridine.sulfur trioxide complex (PST) (2.0 mmol) under argon. Anhydrous MeCN (2.0 mL) was added and the reaction mixture heated at 90 °C (monitored by TLC). After 3 h, tributylamine (2.0 mmol) was added to the reaction mixture and stirred for 30 min at 90 °C. The flask was cooled to room temperature and the solvent removed under reduced pressure to afford the desired sulfate ester as its tributylammonium salt.

*Work-up procedure A* The flask containing the tributylammonium salt was charged with EtOH (30 mL) and sodium 2-ethylhexanoate (5.0 eq. per sulfate group). The reaction mixture was stirred vigorously for 1 h at room temperature. The precipitate was collected by filtration, washed with EtOH (3 × 20 mL) and dried to a constant weight to afford the desired sulfate ester as its sodium salt.

*Work-up procedure B* The flask containing the tributylammonium salt was charged with ethyl acetate (30 mL) and sodium 2-ethylhexanoate (5.0 eq. per sulfate group). The reaction mixture was stirred vigorously for 1 h at room temperature. The precipitate was collected by filtration, washed with ethyl acetate (3 × 20 mL) and dried to a constant weight to afford the desired sulfate ester as its sodium salt.

*Work-up procedure C* The flask containing the tributylammonium salt was charged with MeCN (25 mL) and sodium iodide (5.0 eq. per sulfate group). The reaction mixture was stirred vigorously for 1 h at room temperature. The precipitate was removed by filtration, washed with MeCN (3 × 20 mL) and dried to a constant weight to afford the desired sulfate ester as its sodium salt.

*General procedure 2* Synthetic procedure for the preparation of sodium benzylsulfamates using sulfur trioxide trimethylamine complex and tributylamine. A flame dried 100 mL round bottom flask was charged with the appropriate amine (1.0 mmol) and trimethylamine.sulfur trioxide complex (TMST) (2.0 mmol) under argon. Anhydrous MeCN (2.0 mL) was added and the reaction mixture heated at 60 °C (monitored by TLC). After 30 min, tributylamine (2.0 mmol) was added to the reaction mixture and stirred for 30 min at 60 °C. The flask was cooled to room temperature and the solvent removed under reduced pressure to afford the desired sulfate ester as its tributylammonium salt.

*Work-up procedure A* The flask containing the tributylammonium salt was charged with EtOH (30 mL) and sodium 2-ethylhexanoate (1.5 eq. per sulfate group). The reaction mixture was stirred vigorously for 1 h at room temperature. The precipitate was removed by filtration, washed with EtOH (3 × 20 mL) and dried to a constant weight to afford the desired sulfate ester as its sodium salt.

*Work-up procedure B* The flask containing the tributylammonium salt was charged with MeCN (25 mL) and sodium iodide (1.5 eq. per sulfate group). The reaction mixture was stirred vigorously for 1 h at room temperature. The precipitate was removed by filtration, washed with MeCN (3 × 20 mL) and dried to a constant weight to afford the desired sulfate ester as its sodium salt.

*General procedure 3* Low-temperature preparation of trimethylammonium sulfamate salts using sulfur trioxide trimethylamine complex (Me_3_NSO_3_, TMST). A 25 mL flask was charged with the appropriate amine (1.0 mmol) and TMST (2.0 eq) under argon. Anhydrous MeCN was added (giving a concentration of 0.50 mol dm^–3^ to the limiting reagent), the reaction mixture was heated at 30 °C and monitored by TLC. After reaction completion the flask was cooled to room temperature and the solvent removed under reduced pressure. The reaction was quenched with EtOH (10 mL) and filtered. The solution was evaporated and extracted with H_2_O (10 mL) and ethyl acetate (4 × 40 mL). The organic layer was dried (MgSO_4_), filtered, and the solvent was removed *in vacuo* giving the desired trimethylammonium salt as an oil.

*General procedure 4* In situ preparation of tributylammonium sulfamate salts using sulfur trioxide trimethylamine complex (Me_3_NSO_3_, TMST).

A 25 mL flask was charged with the appropriate amine (1.0 mmol) and tributylamine (2.0 eq) dissolved in anhydrous MeCN (giving a concentration of 0.50 mol dm^–3^ to the limiting reagent) under argon. After addition of TMST (2.0 eq), the reaction mixture was heated at 30 °C and monitored by TLC. After reaction completion the flask was cooled to room temperature and the solvent removed under reduced pressure. The reaction was quenched with EtOH (10 mL) and filtered. The solution was evaporated and extracted with H_2_O (10 mL) and ethyl acetate (4 × 40 mL). The organic layer was dried (MgSO_4_), filtered, and the solvent was removed *in vacuo* giving the desired tributylammonium salt as an oil.

### Example compound characterization

#### Tributylammonium benzyl sulfate (*3a*)

Following general procedure 4: benzyl alcohol (0.10 mL, 1.0 mmol) and tributylamine (0.47 mL, 2.0 mmol) were dissolved in anhydrous MeCN (2.0 mL). After addition of TMST (278 mg, 2.0 mmol) the reaction mixture was heated at 30 °C for 3 h. The crude compound was purified with by silica gel chromatography (DCM-MeOH; 1:9) to yield the title compound as a yellow oil (138 mg, 37%). νmax cm^–1^ 3455 br w, 2960 s, 2933 s, 2874 s, 1455 s, 1258 s, 1198 s; ^1^H NMR (400 MHz, CDCl_3_) δ_H_ 9.57 (s, 1H), 7.35 (dt, *J* = 6.0, 1.6 Hz, 2H), 7.29–7.18 (m, 3H), 5.03 (s, 2H), 3.06–2.80 (m, 6H), 1.82–1.49 (m, 6H), 1.28 (h, *J* = 7.4 Hz, 6H), 0.87 (t, *J* = 7.4 Hz, 9H); ^13^C NMR (101 MHz, CDCl_3_) δ_C_ 136.8, 128.4, 128.3, 128.0, 69.7, 52.7, 29.7, 25.3, 20.0, 13.6; LRMS *m/z* (ESI+) 559.45 (100%, [M + Bu_3_NH]^+^); HRMS *m/z* (ESI+) C_31_H_63_N_2_O_4_S requires 559.4504, found 559.4503 ([M + Bu_3_NH]^+^. Data were consistent with the literature^19^.

#### Sodium benzyl sulfate (*4a*)

Following the general procedure 1: benzyl alcohol (0.1 mL, 1.0 mmol) and sulfur trioxide pyridine complex (318 mg, 2.0 mmol) were dissolved in anhydrous MeCN (2.0 mL) and heated under reflux at 90 °C for 3 h. Tributylamine (0.4 mL, 2.0 mmol) was added to the mixture and stirred for 30 min. After the completion of reaction, the flask was cooled to room temperature and the solvent removed under reduced pressure. The crude product was purified by work up procedure A to yield the title compound as a bright white solid (196 mg, 93%). M.P. 208–210 °C; ^1^H NMR (300 MHz, D_2_O) δ 7.57–7.33 (m, 5H), 5.09 (s, 2H); ^13^C NMR (101 MHz, D_2_O) δ 135.1, 128.7 (CH and C), 128.4), 70.7; LRMS. *m/z* (ESI−) 187.0 ([M^12^C − Na]^+^, 100%), 188.1 ([M^13^C − Na]^+^, 10%); Data were consistent with the literature^19^.

## Supplementary information

Supplementary Information.
