# Supplementary material for: A novel exchange method to access sulfated molecules
Source: Sci Rep. 2020 Oct 6;10:16559. doi: 10.1038/s41598-020-72500-x (PMC7538947; doi:10.1038/s41598-020-72500-x)
Supplement: Supplementary file 1 — Supplementary Information. [file 41598_2020_72500_MOESM1_ESM.pdf]

ELECTRONIC SUPPORTING INFORMATION

A Novel Exchange Method to Access Sulfated Molecules

Jaber A. Alshehri,[1] Anna Mary Benedetti,[1] and Alan M. Jones[1*]

[1]School of Pharmacy, University of Birmingham, Edgbaston, B15 2TT, United Kingdom

* corresponding author: A.M.J. +44 (0) 121 414 7288 a.m.jones.2@bham.ac.uk

CONTENTS

**S1.** General methods	page 3
**S2.** General experimental procedures	page 3-5
**S3.** Compound characterization	page 6-18
**S4.** Copies of ^{1}H, ^{13}C and ^{19}F NMR spectra	page 19-70
**S5.** TBSAB control experiment	page 71
**S6.** References	page 72

S1. General Methods:

All reactions involving moisture sensitive reagents were carried out using standard Schlenk techniques, in a dry reaction vessel under argon. All solvents used under anhydrous conditions were decanted directly from an SPS dispensary or were stored over 4 Å molecular sieves 24 h prior to use.

Solvents used for workup procedures were of technical grade from Sigma-Aldrich, Honeywell, VWR or Fisher Scientific. Unless stated otherwise, solvents were removed by rotary evaporation under reduced pressure between 30-50 °C. All chemical reagents were used as received unless stated otherwise. Reactions were monitored by TLC analysis on Merck silica gel 60 F254 using UV light (254 nm) and/or potassium permanganate.

^{1}H,^{13}C and ^{19}F NMR spectra were recorded either on a Bruker AVIII operating at 300 MHz for 1H and fitted with a 5 mm BBFO probe or on a Bruker AVANCE NEO operating at 400 MHz for 1H fitted with a 5 mm "smart"BBFO probe, respectively. Chemical shift data are reported in parts per million (ppm, δ scale) downfield from tetramethylsilane (TMS: δ 0.0) and referenced internally to the residual proton in the solvent. The deuterated solvents used for NMR analysis were: chloroform ($CDCl_3$: δH 7.26, δC 77.2), dimethyl sulfoxide (d_6-DMSO: δH 2.50, δC 39.5), and deuterium oxide (D_2O: δH 4.79).Coupling constants are given in Hertz (Hz). All individual signals were assigned using 2D NMR spectroscopy (^{1}H-^{1}H-COSY, ^{1}H-^{13}C-HSQC, and ^{1}H-^{13}C-HMBC). The data are presented as follows: chemical shift multiplicity (s = singlet, d = doublet, t = triplet, q = quartet, p = pentet, m = multiple, br = broad and combinations thereof), coupling constant, integration, and assignment.

Mass spectra were recorded on a Waters Xevo G2-XS Tof or Synap G2-S mass spectrometer using Zspray, Electro-spray ionization in negative (ESI-) mode.

Infrared spectra were recorded on a Perkin Elmer® Spectrum 100 FT-IR and a Varian 660-IR FTIR spectrometer using Agilent® Resolution Pro, with absorption maxima (vmax) reported in cm^{-1}. Optical rotations were measured using a Bellingham and Stanley ADP450 Series Peltier® polarimeter at 25 °C using the D line of sodium (589.3 nm) in the indicated concentration and solvent.

S2. General experimental procedures:

General procedure 1. Synthetic procedure for the preparation of sodium benzyl sulfate ester using sulfur trioxide pyridine complex and tributylamine.

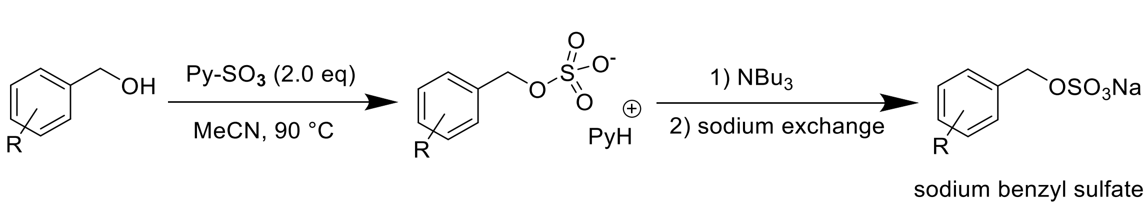

A flame dried 100 mL round bottom flask was charged with the appropriate alcohol (1.0 mmol) and pyridine.sulfur trioxide complex (**PST**) (2.0 mmol) under argon. Anhydrous MeCN (2.0 mL) was added and the reaction mixture heated at 90 °C (monitored by TLC). After 3 h, tributylamine (2.0 mmol) was added to the reaction mixture and stirred for 30 min at 90 °C. The flask was cooled to room temperature and the solvent removed under reduced pressure to afford the desired sulfate ester as its tributylammonium salt.

Work-up procedure A: The flask containing the tributylammonium salt was charged with EtOH (30 mL) and sodium 2-ethylhexanoate (5.0 eq. per sulfate group). The reaction mixture was stirred vigorously for 1 h at room temperature. The precipitate was collected by filtration, washed with EtOH (3 × 20 mL) and dried to a constant weight to afford the desired sulfate ester as its sodium salt.

Work-up procedure B: The flask containing the tributylammonium salt was charged with ethyl acetate (30 mL) and sodium 2-ethylhexanoate (5.0 eq. per sulfate group). The reaction mixture was stirred vigorously for 1 h at room temperature. The precipitate was collected by filtration, washed with ethyl acetate (3 × 20 mL) and dried to a constant weight to afford the desired sulfate ester as its sodium salt.

Work-up procedure C: The flask containing the tributylammonium salt was charged with MeCN (25 mL) and sodium iodide (5.0 eq. per sulfate group). The reaction mixture was stirred vigorously for 1 h at room temperature. The precipitate was removed by filtration, washed with MeCN (3 × 20 mL) and dried to a constant weight to afford the desired sulfate ester as its sodium salt.

General procedure 2. Synthetic procedure for the preparation of sodium benzylsulfamates using sulfur trioxide trimethylamine complex and tributylamine.

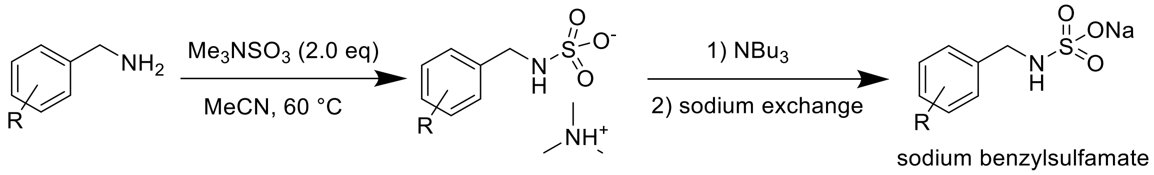

A flame dried 100 mL round bottom flask was charged with the appropriate amine (1.0 mmol) and trimethylamine.sulfur trioxide complex (**TMST**) (2.0 mmol) under argon. Anhydrous MeCN (2.0 mL) was added and the reaction mixture heated at 60 °C (monitored by TLC). After 30 min, tributylamine (2.0 mmol) was added to the reaction mixture and stirred for 30 min at 60 °C. The flask was cooled to room temperature and the solvent removed under reduced pressure to afford the desired sulfate ester as its tributylammonium salt.

Work-up procedure A: The flask containing the tributylammonium salt was charged with EtOH (30 mL) and sodium 2-ethylhexanoate (1.5 eq. per sulfate group). The reaction mixture was stirred vigorously for 1h at room temperature. The precipitate was removed by filtration, washed with EtOH (3 × 20 mL) and dried to a constant weight to afford the desired sulfate ester as its sodium salt.

Work-up procedure B: The flask containing the tributylammonium salt was charged with MeCN (25 mL) and sodium iodide (1.5 eq. per sulfate group). The reaction mixture was stirred vigorously for 1h at room temperature. The precipitate was removed by filtration, washed with MeCN (3 × 20 mL) and dried to a constant weight to afford the desired sulfate ester as its sodium salt.

General procedure 3 Low-temperature preparation of trimethylammonium sulfamate salts using sulfur trioxide trimethylamine complex (Me_3NSO_3, TMST).

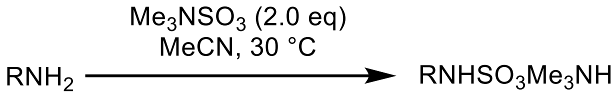

A 25 mL flask was charged with the appropriate amine (1.0 mmol) and **TMST** (2.0 eq) under argon. Anhydrous MeCN was added (giving a concentration of 0.50 mol dm^{-3} to the limiting reagent), the reaction mixture was heated at 30 °C and monitored by TLC. After reaction completion the flask was cooled to room temperature and the solvent removed under reduced pressure. The reaction was quenched with EtOH (10 mL) and filtered. The solution was evaporated and extracted with H_2O (10 mL) and ethyl acetate (4 x 40 mL). The organic layer was dried ($MgSO_4$), filtered, and the solvent was removed *in vacuo* giving the desired trimethylammonium salt as an oil.

General procedure 4. In situ preparation of tributylammonium sulfamate salts using sulfur trioxide trimethylamine complex (Me_3NSO_3, **TMST**).

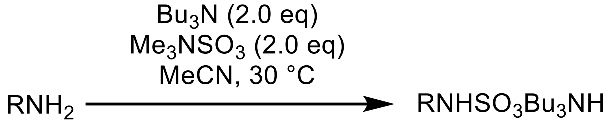

A 25 mL flask was charged with the appropriate amine (1.0 mmol) and tributylamine (2.0 eq) dissolved in anhydrous MeCN (giving a concentration of 0.50 mol dm^{-3} to the limiting reagent) under argon. After addition of **TMST** (2.0 eq), the reaction mixture was heated at 30 °C and monitored by TLC. After reaction completion the flask was cooled to room temperature and the solvent removed under reduced pressure. The reaction was quenched with EtOH (10 mL) and filtered. The solution was evaporated and extracted with H_2O (10 mL) and ethyl acetate (4 x 40 mL). The organic layer was dried ($MgSO_4$), filtered, and the solvent was removed *in vacuo* giving the desired tributylammonium salt as an oil.

S3. Compound characterization:

Tributylammonium benzyl sulfate (**3a**)[1]

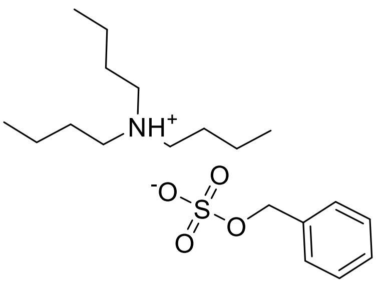

Following **general procedure 4**: benzyl alcohol (0.10 mL, 1.0 mmol) and tributylamine (0.47 mL, 2.0 mmol) were dissolved in anhydrous MeCN (2.0 mL). After addition of **TMST** (278 mg, 2.0 mmol) the reaction mixture was heated at 30 °C for 3 h. The crude compound was purified using silica gel chromatography (DCM-MeOH; 1:9) to yield the title compound as a yellow oil (138 mg, 37%).

vmax cm^{-1} 3455 br w, 2960 s, 2933 s, 2874 s, 1455 s, 1258 s, 1198 s

^{1}H NMR (400 MHz, $CDCl_3$) δ_H 9.57 (s, 1H), 7.35 (dt, *J* = 6.0, 1.6 Hz, 2H), 7.29–7.18 (m, 3H), 5.03 (s, 2H), 3.06–2.80 (m, 6H), 1.8 –1.49 (m, 6H), 1.28 (h, *J* = 7.4 Hz, 6H), 0.87 (t, *J* = 7.4 Hz, 9H).

^{13}C NMR (101 MHz, $CDCl_3$) δ_C 136.8, 128.4, 128.3, 128.0, 69.7, 52.7, 29.7, 25.3, 20.0, 13.6.

LRMS *m/z* (ESI+) 559.45 (100%, $[M+Bu_3NH]^+$)

HRMS *m/z* (ESI+) $C_{31}H_{63}N_2O_4S$ requires 559.4504, found 559.4503 ($[M+Bu_3NH]^+$

Data were consistent with the literature.[1]

Sodium benzyl sulfate (**4a**)[1]

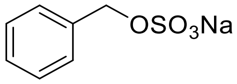

Following the **general procedure 1**: benzyl alcohol (0.1 mL, 1.0 mmol) and sulfur trioxide pyridine complex (318 mg, 2.0 mmol) were dissolved in anhydrous MeCN (2.0 mL) and heated under reflux at 90 °C for 3 h. Tributylamine (0.4 mL, 2.0 mmol) was added to the mixture and stirred for 30 min. After the completion of reaction, the flask was cooled to room temperature and the solvent removed under reduced pressure. The crude product was purified using **work up procedure A** to yield the title compound as a bright white solid (196 mg, 93%).

M.P. 193-195 °C (lit.[1] 194-196 °C)

^{1}H NMR (300 MHz, D_2O) δ 7.57–7.33 (m, 5H), 5.09 (s, 2H).

^{13}C NMR (101 MHz, D_2O) δ 135.1, 128.7 (CH and C), 128.4), 70.7.

LRMS *m/z* (ESI-) 187.0 ($[M^{12}C\text{-}Na]^+$, 100 %), 188.1 ($[M^{13}C\text{-}Na]^+$, 10 %).

Data were consistent with the literature.[1]

Sodium 2-methylbenzyl sulfate (**4b**)

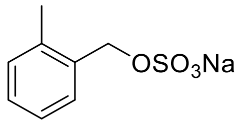

Following **general procedure 1**: 2-methylbenzyl alcohol (122 mg, 1.0 mmol) and sulfur trioxide pyridine complex (318 mg, 2.0 mmol) were dissolved in anhydrous MeCN (2.0 mL) and heated under reflux at 90 °C for 3 h. Tributylamine (0.4 mL, 2.0 mmol) was added to the mixture and stirred for 30 min. After the completion of the reaction, the flask was cooled to room temperature and the solvent removed under reduced pressure. The crude product was purified using **work up procedure B** to yield the title compound as a bright white solid (216 mg, 96%).

M.P. 200-202 °C

IR ν_{max} cm^{-1} 1475w, 1383w, 1247m, 1204s, 1108s, 1071s

^{1}H NMR (400 MHz, D_2O) δ 7.41–7.23 (m, 4H), 5.09 (s, 2H), 2.36 (s, 3H).

^{13}C NMR (101 MHz, D_2O) δ 138.0, 132.9, 130.4, 129.8, 129.3, 126.1, 69.3, 17.7.

LRMS *m/z* (ESI-) 201.0 ([M^{12}C-Na]$^+$, 100 %), 202.0 ([M^{13}C-Na]$^+$**,** 10 %).

HRMS *m/z* (ESI-) $C_8H_9O_4S$ requires 201.0222, found 201.0225 [M-Na]$^+$.

Sodium 3-methylbenzyl sulfate (**4c**)

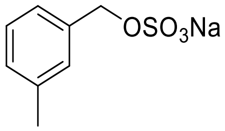

Following **general procedure 1**: 3-methylbenzyl alcohol (0.12 mL, 1.0 mmol) and sulfur trioxide pyridine complex (318 mg, 2.0 mmol) were dissolved in anhydrous MeCN (2.0 mL) and heated under reflux at 90 °C for 3 h. Tributylamine (0.4 mL, mmol) was added to the mixture and stirred for 30 min. After the completion of the reaction, the flask was cooled to room temperature and the solvent removed under reduced pressure. The crude product was purified using **work up procedure A** to yield the title compound as a bright white solid (190 mg, 85%).

M. P. 172-174 °C

IR ν_{max} cm^{-1} 2904w, 1469w, 1380w, 1248s, 1203s, 1130s

^{1}H NMR (400 MHz, D_2O) δ 7.50–7.08 (m, 4H), 5.03 (s, 2H), 2.34 (s, 3H).

^{13}C NMR (101 MHz, D_2O) δ 138.9, 135.1, 129.3, 129.0, 128.7, 125.3, 70.7, 20.3.

LRMS *m/z* (ESI-) 201.0 ([M^{12}C-Na]$^+$, 100%), 202.0 ([M^{13}C-Na]$^+$, 10%).

HRMS *m/z* (ESI-) $C_8H_9O_4S$ requires 201.0222, found 201.0228 [M-Na]$^+$.

Sodium 4-methylbenzyl sulfate (**4d**)

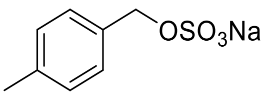

Following **general procedure 1**: 4-methylbenzyl alcohol (122 mg, 1.0 mmol) and sulfur trioxide pyridine complex (318 mg, 2.0 mmol) were dissolved in anhydrous MeCN (2.0 mL) and heated under reflux at 90 °C for 3 h. Tributylamine (0.4 mL, 2.0 mmol) was added to the mixture and stirred for 30 min. After the completion of the reaction, the flask was cooled to room temperature and the solvent removed under reduced pressure. The crude product was purified using **work up procedure C** to yield the title compound as a bright white solid (212 mg, 94%).

M. P. 182-184 °C

IR ν_{max} cm^{-1} 1615w, 1469w, 1380w, 1252s, 1202s, 1072s

^{1}H NMR (300 MHz, D_2O) δ 7.39–7.34 (m, 2H), 7.32–7.26 (m, 2H), 5.04 (s, 2H), 2.35 (s, 3H).

^{13}C NMR (101 MHz, D_2O) δ 139.1, 132.0, 129.3, 128.6, 70.7, 20.2.

LRMS *m/z* (ESI-) 201.0 ([M^{12}C-Na]$^+$, 100%), 202.0 ([M^{13}C-Na]$^+$, 10%).

HRMS *m/z* (ESI-) $C_8H_9O_4S$ requires 201.0222, found 201.0222 [M-Na]$^+$.

Sodium 2-chlorobenzyl sulfate (**4e**)[1]

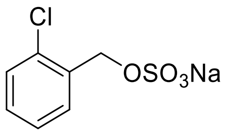

Following the **general procedure 1**: 2-chlorobenzyl alcohol (142.5 mg, 1.0 mmol) and sulfur trioxide pyridine complex (318 mg, 2.0 mmol) were dissolved in anhydrous MeCN (2.0 mL) and heated under reflux at 90 °C for 3 h. Tributylamine (0.4 mL, 2.0 mmol) was added to the mixture and stirred for 30 min. After the completion of the reaction, the flask was cooled to room temperature and the solvent removed under reduced pressure. The crude product was purified using **work up procedure C** to yield the title compound as a yellow solid (180 mg, 73%).

M.P. 239-241°C (lit.[1] 219-221°C)

^{1}H NMR (300 MHz, D_2O) δ 7.58–7.48 (m, 2H), 7.45–7.34 (m, 2H), 5.20 (s, 2H).

^{13}C NMR (101 MHz, D_2O) δ 133.5, 132.4, 130.7, 130.4, 129.5, 127.2, 68.1.

LRMS *m/z* (ESI-) 220.9 ([M^{35}Cl-Na]$^+$, 100%), 222.9 ([M^{37}Cl-Na]$^+$, 40%).

HRMS *m/z* (ESI-) $C_7H_6O_4SCl$ requires 220.9675, found 220.9680 [M^{35}Cl-Na]$^+$.

Data were consistent with the literature.[1]

Sodium 3-chlorobenzyl sulfate (**4f**)

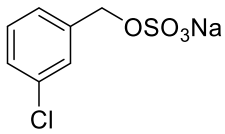

Following **general procedure 1**: 3-chlorobenzyl alcohol (0.11 mL, 1.0 mmol) and sulfur trioxide pyridine complex (318 mg, 2.0 mmol) were dissolved in anhydrous MeCN (2.0 mL) and heated under reflux at 90 °C for 3 h. Tributylamine (0.4 mL, 2.0 mmol) was added to the mixture and stirred for 30 min. After the completion of the reaction, the flask was cooled to room temperature and the solvent removed under reduced pressure. The crude product was purified using **work up procedure A** to yield the title compound as a bright white solid (201 mg, 82%).

M.P. 219- 221 °C

IR ν_{max} cm^{-1} 1573w, 1469w, 1378w, 1253m, 1205m, 1106s, 611s

^{1}H NMR (400 MHz, D_2O) δ 7.59–7.23 (m, 4H), 5.04 (s, 2H).

^{13}C NMR (101 MHz, D_2O) δ 137.3, 133.7, 130.1, 128.5, 128.0, 126.4, 69.7.

LRMS *m/z* (ESI-) 220.9 ([M^{35}Cl-Na]$^+$, 100%), 222.9 ([M^{37}Cl-Na]$^+$, 40%).

HRMS *m/z* (ESI-) $C_7H_6O_4SCl$ requires 220.9675, found 220.9681 [M^{35}Cl-Na]$^+$.

Sodium 4-chlorobenzyl sulfate (**4g**)[1]

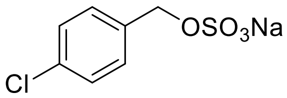

Following **general procedure 1**: 4-chlorobenzyl alcohol (142.5 mg, 1.0 mmol) and sulfur trioxide pyridine complex (318 mg, 2.0 mmol) were dissolved in anhydrous MeCN (2.0 mL) and heated under reflux at 90 °C for 3 h. Tributylamine (0.4 mL, 2.0 mmol) was added to the mixture and stirred for 30 min. After the completion of the reaction, the flask was cooled to room temperature and the solvent removed under reduced pressure. The crude product was purified using **work up procedure C** to yield the title compound as a bright white solid (220 mg, 90%).

M.P. 227-229 °C (lit.[1] 202-204 °C)

^{1}H NMR (400 MHz, D_2O) δ 7.45–7.38 (m, 4H), 5.04 (s, 2H).

^{13}C NMR (101 MHz, D_2O) δ 133.9, 129.9, 128.7, 69.9.

LRMS *m/z* (ESI-) 220.9 ([M^{35}Cl-Na]$^+$, 100%), 222.9 ([M^{37}Cl-Na]$^+$, 40%).

HRMS *m/z* (ESI-) $C_7H_6O_4SCl$ requires 220.9675, found 220.9681 [M^{35}Cl-Na]$^+$.

Data were consistent with the literature.[1]

Sodium3-(trifluoromethyl) benzyl sulfate (**4h**)

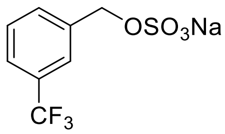

Following **general procedure 1**: 3-(trifluoromethyl) benzyl alcohol (0.13 mL, 1.0 mmol) and sulfur trioxide pyridine complex (318 mg, 2.0 mmol) were dissolved in anhydrous MeCN (2.0 mL) and heated under reflux at 90 °C for 3 h. Tributylamine (0.4 mL, 2.0 mmol) was added to the mixture and stirred for 30 min. After the completion of the reaction, the flask was cooled to room temperature and the solvent removed under reduced pressure. The crude product was purified using **work up procedure C** to yield the title compound as a white solid (274 mg, 98%).

M.P. 224-226 °C

IR ν_{max} cm^{-1} 3109w, 1470w, 1452w, 1329w, 1250w, 1203m, 1069m

^{1}H NMR (300 MHz, D_2O) δ 7.83–7.56 (m, 4H), 5.14 (s, 2H).

^{13}C NMR (101 MHz, D_2O) δ 136.2, 131.7, 130.1 (q, $^{2}J_{C-F}$ = 32.2 Hz), 129.3, 125.3 (q, $^{3}J_{C-F}$ = 3.9 Hz), 124.8 (q, $^{3}J_{C-F}$ = 4.1 Hz), 123.5 (q, $^{1}J_{C-F}$ = 271.2 Hz), 69.7.

^{19}F NMR (377 MHz, D2O) δ -62.42.

LRMS *m/z* (ESI-) 254.9 ([M^{12}C-Na]$^{+}$, 100%), 254.9 ([M^{13}C-Na]$^{+}$, 10%).

HRMS *m/z* (ESI-) $C_8H_6O_4F_3S$ requires 254.9939, found 254.9946 [M-Na]$^{+}$.

Sodium 4-methoxybenzyl sulfate (**4i**)[1]

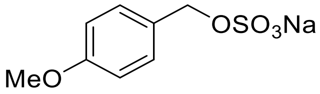

Following **general procedure 1**: 4-methoxybenzyl alcohol (0.12 mL, 1.0 mmol) and sulfur trioxide pyridine complex (318 mg, 2.0 mmol) were dissolved in anhydrous MeCN (2.0 mL) and heated under reflux at 90 °C for 3 h. Tributylamine (0.4 mL, 2.0 mmol) was added to the mixture and stirred for 30 min. After the completion of the reaction, the flask was cooled to room temperature and the solvent removed under reduced pressure. The crude product was purified using **work up procedure C** to yield the title compound as a bright white solid (170 mg, 70%).

M.P. >235°C (dec.)

^{1}H NMR (400 MHz, D_2O) δ 7.41–7.23 (m, 2H), 6.98 (d, *J* = 8.7 Hz, 2H), 4.54 (s, 2H), 3.80 (s, 3H).

^{13}C NMR (101 MHz, D_2O) δ 158.3, 132.8, 129.2, 114.0, 63.4, 55.3.

LRMS *m/z* (ESI-) 217.0 ([M^{12}C-Na]$^{+}$, 100%), 218.0 ([M^{13}C-Na]$^{+}$, 10%).

HRMS *m/z* (ESI-) $C_8H_9O_5S$ requires 217.0171, found 217.0177 [M-Na]$^{+}$.

Sodium 2-nitrobenzyl sulfate (**4j**)[1]

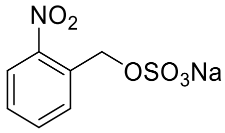

Following **general procedure 1**: 2-nitrobenzyl alcohol (153 mg, 1.0 mmol) and sulfur trioxide pyridine complex (318 mg, 2.0 mmol) were dissolved in anhydrous MeCN (2.0 mL) and heated under reflux at 90 °C for 3 h. Tributylamine (0.4 mL, 2.0 mmol) was added to the mixture and stirred for 30 min. After the completion of the reaction, the flask was cooled to room temperature and the solvent removed under reduced pressure. The crude product was purified using **work up procedure C** to yield the title compound as a yellow solid (166 mg, 65%).

M.P. 228-230 °C

^{1}H NMR (300 MHz, D_2O) δ 8.14 (d, 1H), 7.83–7.70 (m, 2H), 7.59 (d, *J* = 9.0, 6.5, 2.6 Hz, 1H), 5.44 (s, 2H).

^{13}C NMR (101 MHz, D_2O) δ 146.9, 134.4, 131.2, 129.4, 129.2, 124.9, 67.1.

LRMS *m/z* (ESI-) 231.9 ($[M\text{-}Na]^+$, 100%).

HRMS *m/z* (ESI-) $C7H_6NO_6S$ requires 231.9916, found 231.9918 $[M\text{-}Na]^+$.

Data were consistent with the literature.[1]

Sodium 4-nitrobenzyl sulfate (**4k**)[1]

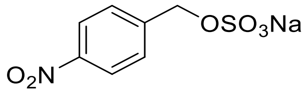

Following the **general procedure 1**: 4-nitrobenzyl alcohol (153 mg, 1.0 mmol) and sulfur trioxide pyridine complex (318.3 mg, 2.0 mmol) were dissolved in anhydrous MeCN (2.0 mL) and heated under reflux at 90 °C for 3 h. Tributylamine (0.4 mL, 2.0 mmol) was added to the mixture and stirred for 0.5 h. After the completion of the reaction, the flask was cooled to room temperature and the solvent removed under reduced pressure. The crude product was purified using **work up procedure C** to yield the title compound as a white solid (167 mg, 66%).

M.P. 188-190 °C

^{1}H NMR (300 MHz, D_2O) δ 8.39 – 8.13 (m, 2H), 7.76 – 7.53 (m, 2H), 5.19 (s, 2H).

^{13}C NMR (101 MHz, D_2O) δ 147.3, 143.1, 128.4, 123.7, 69.0.

LRMS *m/z* (ESI-) 231.9 ($[M^{12}C\text{-}Na]^+$, 100%), 232.9 ($[M^{13}C\text{-}Na]^+$, 10%).

HRMS *m/z* (ESI-) $C7H_6NO_6S$ requires 231.9916, found 231.9922 $[M\text{-}Na]^+$.

Data were consistent with the literature.[1]

Trimethylammonium benzyl sulfamate (**6a**)[2]

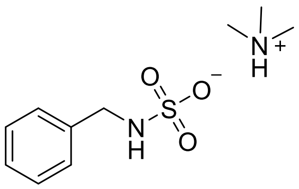

Following **general procedure 3**: benzyl amine (0.109 mL, 1.0 mmol) and **TMST** (278.3 mg, 2.0 mmol) were dissolved in anhydrous MeCN (2.0 mL) and the reaction mixture was heated at 30 °C for 3 h. After purification the title compound was afforded as a clear oil (122 mg, 50%).

IR ν_{max} cm^{-1} 3444w, 3033w, 2928w, 1453w, 1157w, 1023w

^{1}H NMR δ_H (400 MHz, $CDCl_3$) 9.93 (s, 1H), 7.29 – 7.25 (m, 2H), 7.20 – 7.09 (m, 3H), 4.10 (s, 2H), 3.34 (s, 1H), 2.72 (s, 9H).

^{13}C NMR δ_C (101 MHz, $CDCl_3$) 139.3, 128.5, 128.3, 127.2, 48.2, 45.5.

Data were consistent with the literature.[2]

Tributylammonium benzyl sulfamate (**7a**)[2]

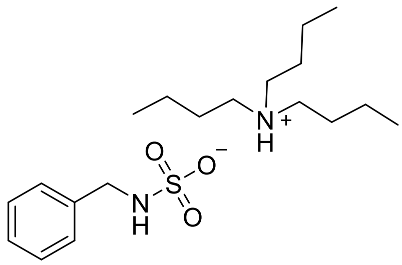

Following **general procedure 4**: benzyl amine (0.109 mL, 1.0 mmol) and tributylamine (0.47 mL, 2.0 mmol) were dissolved in anhydrous MeCN (2.0 mL). After addition of **TMST** (278.3 mg, 2.0 mmol) the reaction mixture was heated at 30 °C for 3 h. The title compound was obtained as a clear oil (317 mg, 84%) after purification.

IR ν_{max} cm^{-1} 3456w, 2960w, 1453w, 1287w, 1233w, 1164w, 1025w

^{1}H NMR δ_H (400 MHz, d_6-DMSO) 8.86 (s, 1H), 7.35 - 7.12 (m, 5H), 4.86 (s, 1H), 3.93 (s, 2H), 3.05 - 2.91 (m, 6H), 1.58 (ddt, *J* = 12.0, 8.0, 6.0 Hz, 6H), 1.30 (h, *J* = 7.5 Hz, 6H), 0.90 (t, *J* = 7.5 Hz, 9H)

^{13}C NMR δ_C (101 MHz, d_6-DMSO) 140.7, 127.9, 127.6, 126.2, 51.8, 47.3, 25.0, 19.4, 13.5

LRMS *m/z* (ESI+) 558.47 ($[M+Bu_3NH]^+$, 20%), 186.23 ($[Bu_3NH]^+$, 100%)

HRMS *m/z* (ESI+) $C_{31}H_{64}N_3O_3S$ requires 558.4668, found 558.4663 ($[M-H]^+$).

Data were consistent with the literature.[2]

Tributylammonium 3-chloro benzyl sulfamate (**7e**)

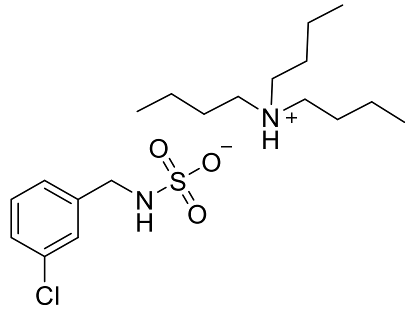

Following **general procedure 4**: 3-chloro benzyl amine (0.12 mL, 1.0 mmol) and tributylamine (0.47 mL, 2.0 mmol) were dissolved in anhydrous MeCN (2.0 mL). After addition of **TMST** (278.3 mg, 2.0 mmol) the reaction mixture was heated at 30 °C for 3 h. The title compound was obtained as a clear oil (382 mg, 93%) after purification.

IR ν_{max} cm^{-1} 3251w, 2960w, 2873w, 1470w, 1159w, 1030w, 682w

^{1}H NMR δ_H (400 MHz, $CDCl_3$) 7.38 (d, *J* = 1.0 Hz, 1H), 7.29 – 7.12 (m, 3H), 4.22 – 4.17 (m, 2H), 3.92 (s, 1H), 3.01 – 2.84 (m, 6H), 1.71 – 1.59 (m, 6H), 1.34 (h, *J* = 7.5 Hz, 6H), 0.92 (t, *J* = 7.5 Hz, 9H).

^{13}C NMR δ_C (101 MHz, $CDCl_3$) 141.6, 134.1, 129.6, 128.1, 127.1, 126.1, 52.5, 47.6, 25.5, 20.1, 13.7.

Tributylammonium 3-methoxy benzyl sulfamate (**7f**)

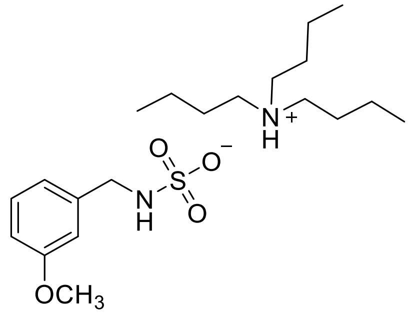

Following **general procedure 4**: 3-methoxy benzyl amine (0.13 mL, 1.0 mmol) and tributylamine (0.47 mL, 2.0 mmol) were dissolved in anhydrous MeCN (2.0 mL). After addition of **TMST** (278.3 mg, 2.0 mmol) the reaction mixture was heated at 30 °C for 3 h. The title compound was obtained as a clear oil (197 mg, 50%) after purification.

IR ν_{max} cm^{-1} 3444w, 2960w, 1458w, 1232w, 1154w, 1027w

^{1}H NMR δ_H (400 MHz, $CDCl_3$) 10.15 (s, 1H), 7.23 – 7.15 (m, 1H), 6.98 – 6.90 (m, 2H), 6.76 (ddd, *J* = 8.0, 2.5, 1.0 Hz, 1H), 4.22 (s, 2H), 3.77 (s, 3H), 3.08 – 2.91 (m, 6H), 1.76 – 1.62 (m, 6H), 1.37 (h, *J* = 7.5 Hz, 6H), 0.95 (t, *J* = 7.5 Hz, 9H).

^{13}C NMR δ_C (101 MHz, $CDCl_3$) 159.8, 140.8, 129.4, 120.4, 113.4, 113.0, 55.3, 48.3, 25.3, 20.1, 13.7.

Sodium benzyl sulfamate (**8a**)[1]

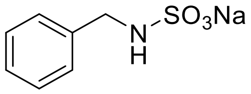

Following the **general procedure 2**: Benzylamine (0.1 mL, 1.0 mmol) and sulfur trioxide trimethylamine complex (278 mg, 2.0 mmol) were dissolved in anhydrous MeCN (2.0 mL) and heated under reflux at 60 °C for 30 mins. Tributylamine (0.4 mL, 2.0 mmol) was added to the mixture and stirred for 30 mins. After the completion of the reaction, the flask was cooled to room temperature and the solvent removed under reduced pressure. The crude compound was purified using **work up procedure B** to yield the title compound as a white solid (208 mg, 99%).

M.P. 238-240 °C (Lit. >230 °C)[2]

^{1}H NMR (400 MHz, D_2O) δ 7.43–7.30 (m, 5H), 4.12 (s, 2H).

^{13}C NMR (101 MHz, D_2O) δ 137.9, 128.6, 128.2, 127.5, 47.3.

LRMS *m/z* (ESI-) 186.0 ([M^{12}C-Na]$^+$, 100%), 187.0 ([M^{13}C-Na]$^+$, 10%).

HRMS *m/z* (ESI-) $C_7H_8NO_3S$ requires 186.0225, found 186.0228 [M-Na]$^+$.

Data were consistent with the literature.[1]

Sodium (3-methylbenzyl) sulfamate (**8b**)

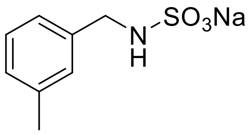

Following the **general procedure 2**: 3-methylbenzylamine (0.12 mL, 1.0 mmol) and sulfur trioxide trimethylamine complex (278 mg, 2.0 mmol) were dissolved in anhydrous MeCN (2.0 mL) and heated under reflux at 60 °C for 30 mins. Tributylamine (0.4 mL, 2.0 mmol) was added to the mixture and stirred for 30 mins. After the completion of the reaction, the flask was cooled to room temperature and the solvent removed under reduced pressure. The crude compound was purified using **work up procedure A** to yield the title compound as a white solid (209 mg, 94%).

M.P. 224- 226 °C

IR v_{max} cm^{-1} 3299w, 2907w, 1608w, 1400w, 1343w, 1166s

^{1}H NMR (300 MHz, D_2O) δ 7.37–7.14 (m, 4H), 4.10 (s, 2H), 2.35 (s, 3H).

^{13}C NMR (101 MHz, D_2O) δ 138.7, 138.0, 128.8, 128.6, 128.1, 125.1, 47.2, 20.3.

LRMS *m/z* (ESI-) 200.0378 ([M^{12}C-Na]$^+$, 100%), 201.0406 ([M^{13}C-Na]$^+$, 10%).

HRMS *m/z* (ESI-) $C_8H_{10}NO_3S$ requires 200.0381, found 200.0378 [M-Na]$^+$.

Sodium (4-methylbenzyl) sulfamate (**8c**)

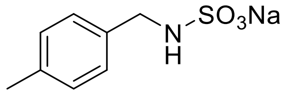

Following the **general procedure 2**: 4-methylbenzylamine (0.13 mL, 1.0 mmol) and sulfur trioxide trimethylamine complex (278 mg, 2.0 mmol) were dissolved in anhydrous MeCN (2.0 mL) and heated under reflux at 60 °C for 30 mins. Tributylamine (0.4 mL, 2.0 mmol) was added to the mixture and stirred for 30 mins. After the completion of the reaction, the flask was cooled to room temperature and the solvent removed under reduced pressure. The crude compound was purified using **work up procedure A** to yield the title compound as a white solid (222 mg, 99%).

M.P. 234-236 °C

IR ν_{max} cm^{-1} 3300w, 1514w, 1403w, 1341w, 1167w, 1058m

^{1}H NMR (300 MHz, D_2O) δ 7.35 – 7.22 (m, 4H), 4.10 (s, 2H), 2.33 (s, 3H).

^{13}C NMR (101 MHz, D_2O) δ 137.6, 134.8, 129.2, 128.3, 47.0, 20.1.

LRMS *m/z* (ESI-) 200.0376 ([M^{12}C-Na]$^+$, 100%), 201.0399 ([M^{13}C-Na]$^+$, 20%).

HRMS *m/z* (ESI-) $C_8H_{10}NO_3S$ requires 200.0381, found 200.0376 [M-Na]$^+$.

Preparation of Sodium (3-chlorobenzyl) sulfamate (**8d**)[2]

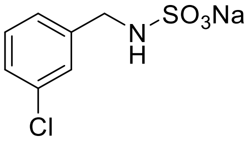

Following the **general procedure 2**: 3-chlorobenzylamine (0.12 mL, 1.0 mmol) and sulfur trioxide trimethylamine complex (278 mg, 2.0 mmol) were dissolved in anhydrous MeCN (2.0 mL) and heated under reflux at 60 °C for 30 mins. Tributylamine (0.4 mL, 2.0 mmol) was added to the mixture and stirred for 30 mins. After the completion of the reaction, the flask was cooled to room temperature and the solvent removed under reduced pressure. The crude compound was purified using **work up procedure A** to yield the title compound as a white solid (220 mg, 90%).

M.P. 223-225 °C (lit.[2] 223-225 °C)

^{1}H NMR (300 MHz, D_2O) δ 7.47–7.43 (m, 1H), 7.38–7.30 (m, 3H), 4.12 (s, 2H).

^{13}C NMR (101 MHz, D_2O) δ 140.2, 133.5, 130.0, 127.9, 127.3, 126.4, 46.6.

LRMS *m/z* (ESI-) 219.98 ([M^{35}Cl-Na]$^+$, 100%), 221.98 ([M^{37}Cl-Na]$^+$, 40%).

HRMS *m/z* (ESI-) $C_7H_7O_3NS$ Cl requires 219.9841, found 219.9835 [M^{35}Cl-Na]$^+$.

Data were consistent with the literature.[2]

Preparation of Sodium (4-chlorobenzyl) sulfamate (**8e**)[2]

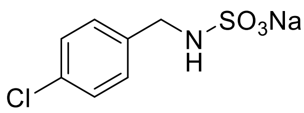

Following the **general procedure 2**: 4-chlorobenzylamine (0.12 mL, 1.0 mmol) and sulfur trioxide trimethylamine complex (278 mg, 2.0 mmol) were dissolved in anhydrous MeCN (2.0 mL) and heated under reflux at 60 °C for 30 mins. Tributylamine (0.4 mL, 2.0 mmol) was added to the mixture and stirred for 30 mins. After the completion of the reaction, the flask was cooled to room temperature and the solvent removed under reduced pressure. The crude compound was purified using **work up procedure A** to yield the title compound as a white solid (240 mg, 98%).

M.P. 234-236 °C (lit.[2] 202-203 °C)

^{1}H NMR (300 MHz, D_2O) δ 7.43 – 7.35 (m, 4H), 4.11 (s, 2H).

^{13}C NMR (101 MHz, D_2O) δ 136.7, 132.5, 129.7, 128.4, 46.6.

LRMS *m/z* (ESI-) 219.9835 ([M^{35} Cl -Na]$^{+}$, 100 %), 221.9806 ([M^{37}Cl-Na]$^{+}$, 40 %), 220.9863 ([C^{13} M^{35} Cl -Na]$^{+}$, 10%).

HRMS *m/z* (ESI-) $C_7H_7O_3NS$ Cl requires 219.9839, found 219.9835 [M^{35}Cl-Na]$^{+}$.

Data were consistent with the literature.[2]

Sodium (3-methoxybenzyl) sulfamate (**8f**)[2]

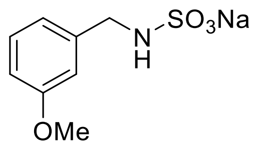

Following the **general procedure 2**: 3-methoxybenzylamine (0.13 mL, 1.0 mmol) and sulfur trioxide trimethylamine complex (278 mg, 2.0 mmol) were dissolved in anhydrous MeCN (2.0 mL) and heated under reflux at 60 °C for 30 mins. Tributylamine (0.4 mL, 2.0 mmol) was added to the mixture and stirred for 30 mins. After the completion of the reaction, the flask was cooled to room temperature and the solvent removed under reduced pressure. The crude compound was purified using **work up procedure A** to yield the title compound as a white solid (224 mg, 93 %).

M.P. 219-221 °C (lit.[2] 218-220 °C)

^{1}H NMR (300 MHz, D_2O) δ 7.38–7.30 (m, 1H), 7.07–7.00 (m, 2H), 6.97–6.89 (m, 1H), 4.11 (s, 2H), 3.83 (s, 3H).

^{13}C NMR (101 MHz, D_2O) δ 158.8, 139.9, 129.8, 120.9, 113.5, 113.1, 55.2, 47.1**.**

LRMS *m/z* (ESI-) 216.0327 ([M^{12}C-Na]$^{+}$, 100%), 217.0358 ([M^{13}C-Na]$^{+}$, 10%).

HRMS *m/z* (ESI-) $C_8H_{10}NO_4S$ requires 216.0331, found 216.0327 [M-Na]$^{+}$.

Data were consistent with the literature.[2]

Sodium (4-metheoxybenzyl) sulfamate (**8g**)[2]

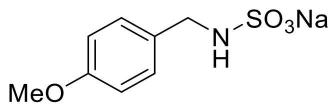

Following the **general procedure 2**: 4-methoxybenzylamine (0.13 mL, 1.0 mmol) and sulfur trioxide trimethylamine complex (278 mg, 2.0 mmol) were dissolved in anhydrous MeCN (2.0 mL) and heated under reflux at 60 °C for 30 mins. Tributylamine (0.4 mL, 2.0 mmol) was added to the mixture and stirred for 30 mins. After the completion of the reaction, the flask was cooled to room temperature and the solvent removed under reduced pressure. The crude compound was purified using **work up procedure A** to yield the title compound as a white solid (238 mg, 99%).

M.P. 235-237 °C (Lit.[2] 232-237 °C)

^{1}H NMR (300 MHz, D_2O) δ 7.40–7.30 (m, 2H), 7.03–6.93 (m, 2H), 4.07 (s, 2H), 3.82 (s, 3H).

^{13}C NMR (101 MHz, D_2O) δ 158.1, 130.5, 129.7, 114.0, 55.3, 46.7.

LRMS *m/z* (ESI-) 216.0329 ([M^{12}C-Na]$^+$, 100%), 217.0364 ([M^{13}C-Na]$^+$, 10%).

HRMS *m/z* (ESI-) $C_8H_{10}NO_4S$ requires 216.0331, found 216.0329 [M-Na]$^+$.

Data were consistent with the literature.[2]

Tributylammonium (1-phenylethyl) sulfamate (**9**)

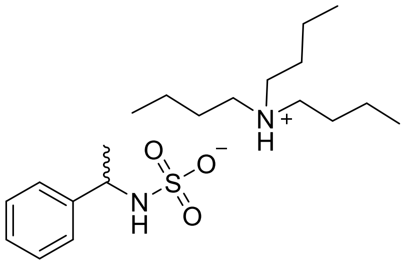

Following **general procedure 4**: (+/-)-1-phenyl-ethyl amine (0.13 mL, 1.0 mmol) and tributylamine (0.47 mL, 2.0 mmol) were dissolved in anhydrous MeCN (2.0 mL). After addition of **TMST** (278.3 mg, 2.0 mmol) the reaction mixture was heated at 30 °C for 3 h. The title compound was obtained as a white solid (365 mg, 94%) after purification.

M.P. 78-83 °C

IR ν_{max} cm^{-1} 3223w, 2959w, 1457w, 1214w, 1157w, 1029w

^{1}H NMR δ_H (400 MHz, $CDCl_3$) 7.41 – 7.38 (m, 2H), 7.30 – 7.25 (m, 2H), 7.21 – 7.16 (m, 1H), 4.58 (q, *J* = 7.0 Hz, 1H), 3.90 (s, 1H), 2.93 – 2.78 (m, 6H), 1.66 – 1.58 (m, 6H), 1.51 (d, *J* = 7.0 Hz, 3H), 1.34 (h, *J* = 7.5 Hz, 6H), 0.94 (t, *J* = 7.5 Hz, 9H).

^{13}C NMR δ_C (101 MHz, $CDCl_3$) 145.8, 128.3, 126.8, 126.7, 54.0, 52.4, 25.5, 24.4, 20.2, 13.8.

[α]$_D^{25}$ 0.346 (c. 1.0, MeOH)

Tributylammonium phenyl sulfamate (**10**)

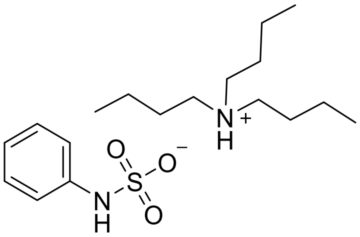

Following **general procedure 4**: aniline (0.10 mL, 1.0 mmol) and tributylamine (0.47 mL, 2.0 mmol) were dissolved in anhydrous MeCN (2.0 mL). After addition of **TMST** (278.3 mg, 2.0 mmol) the reaction mixture was heated at 30 °C for 3 h. The title compound was obtained as a white solid (206 mg, 57%) after purification.

M.P. 120-125 °C

IR ν_{max} cm^{-1} 3251w, 2962w, 1496w, 1226w, 1173w, 1031w

^{1}H NMR δ_H (400 MHz, $CDCl_3$) 9.86 (s, 1H), 7.24 – 7.16 (m, 4H), 6.91 (tt, *J* = 7.0, 1.5 Hz, 1H), 3.04 – 2.91 (m, 6H), 1.73 – 1.55 (m, 6H), 1.33 (h, *J* = 7.5 Hz, 6H), 0.92 (t, *J* = 7.5 Hz, 9H)

^{13}C NMR δ_C (101 MHz, $CDCl_3$) 141.6, 129.0, 121.6, 118.5, 52.5, 25.3, 20.1, 13.7.

S4. Copies of ^{1}H, ^{13}C and ^{19}F NMR spectra

^{1}H-NMR spectrum of **3a** (400 MHz, $CDCl_3$)

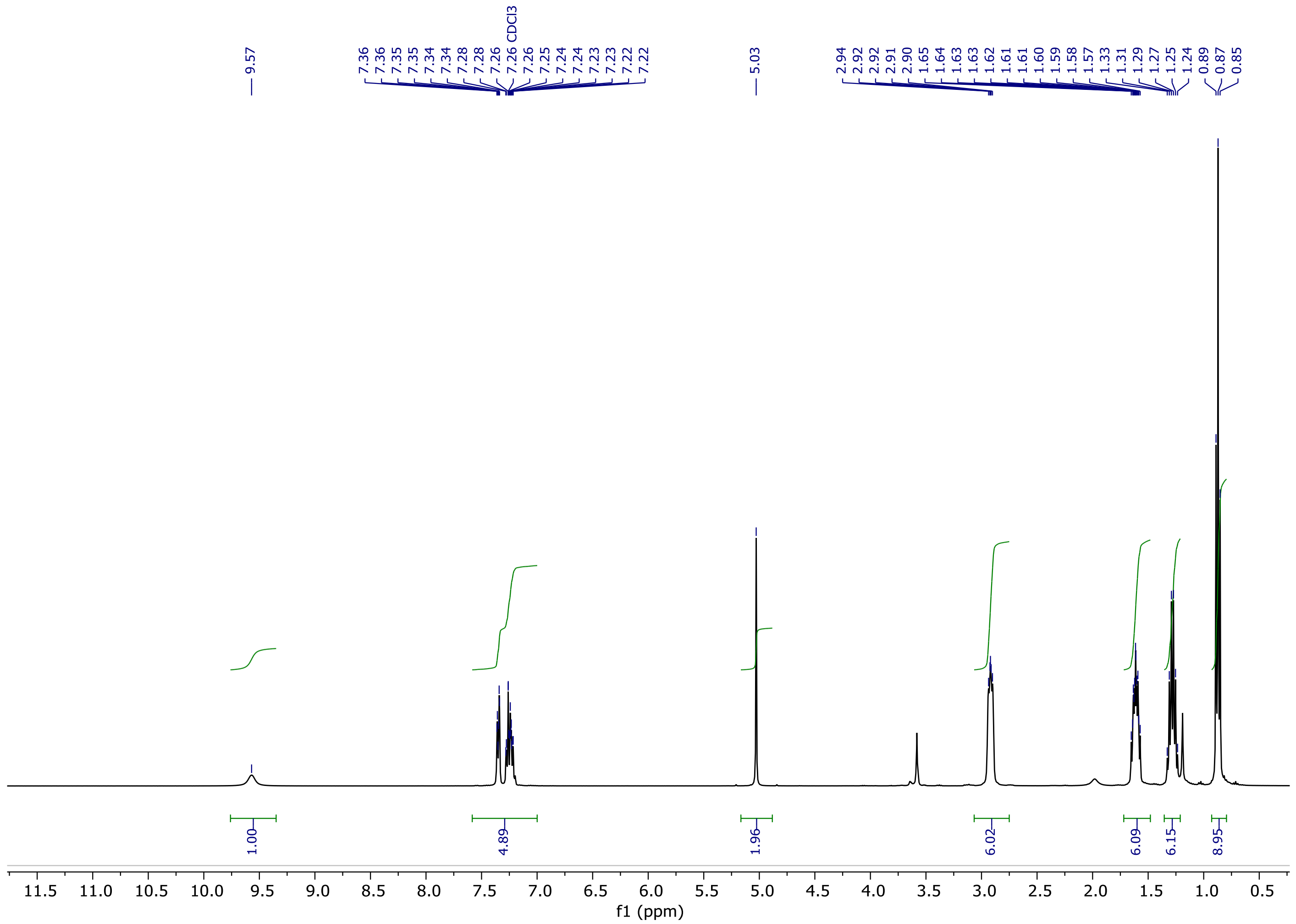

^{13}C-NMR spectrum of **3a** (101 MHz, $CDCl_3$)

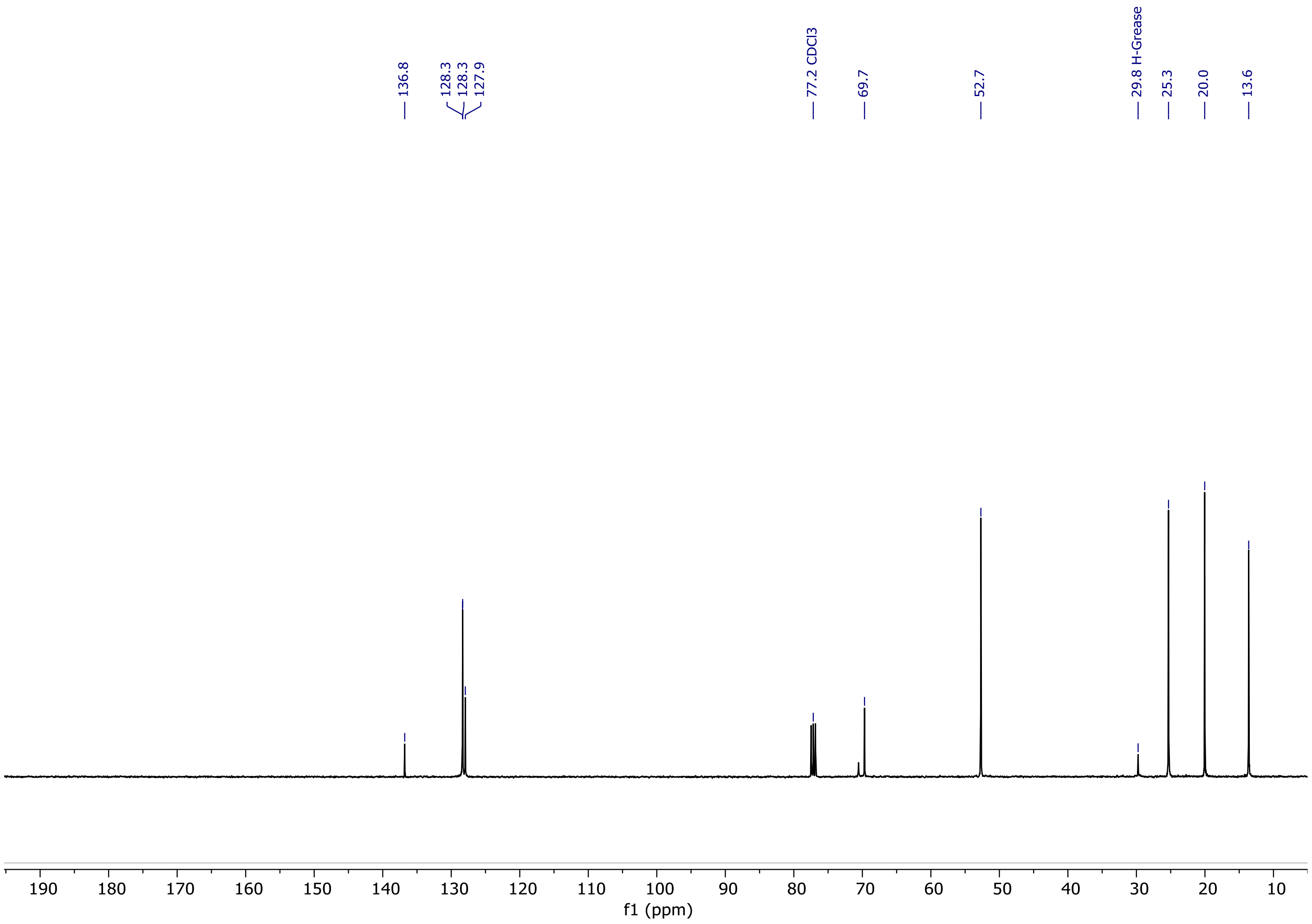

^{1}H NMR spectrum of **4a** (300 MHz, D_2O)

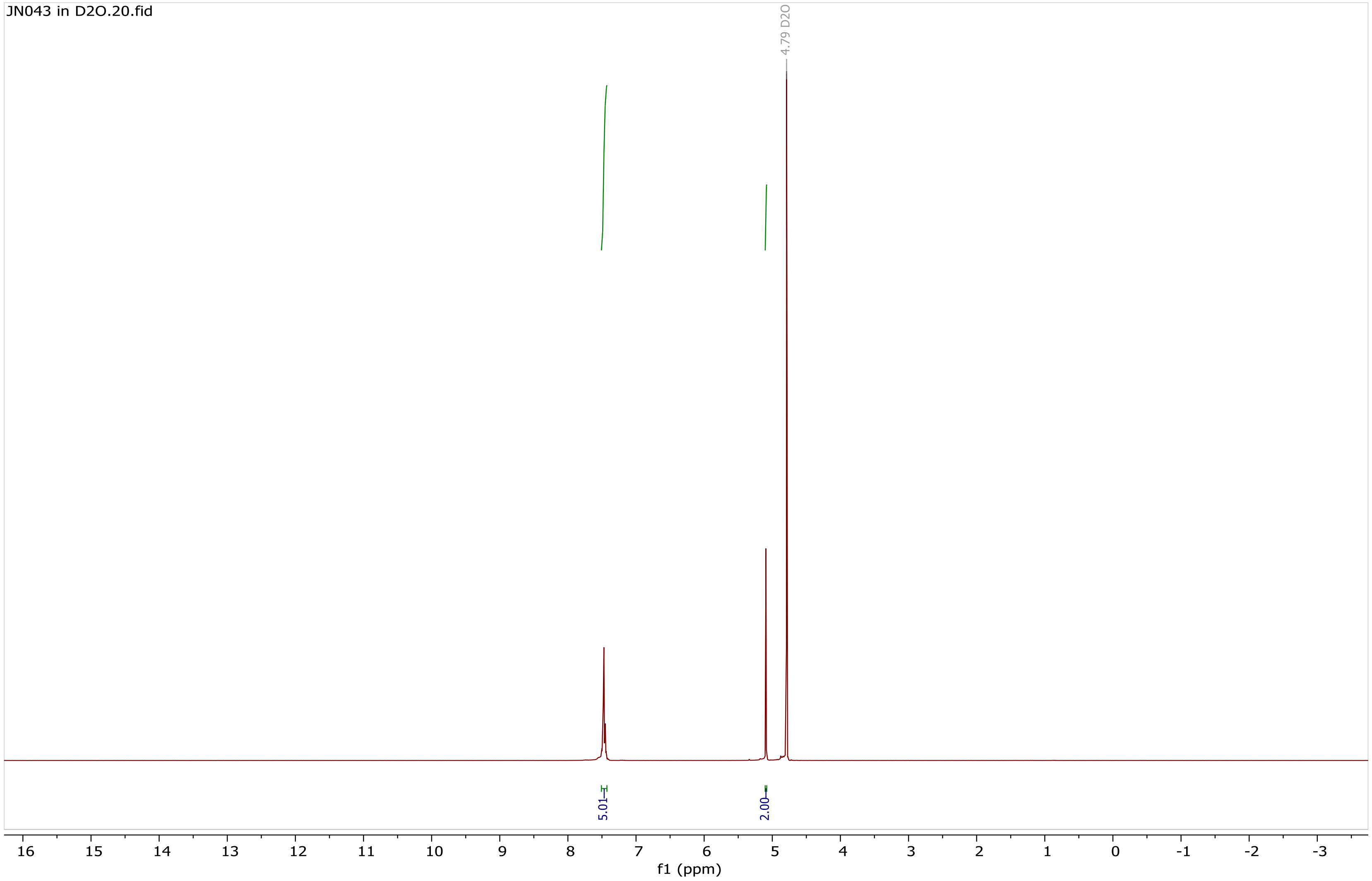

^{13}C NMR spectrum of **4a** (400 MHz, D_2O)

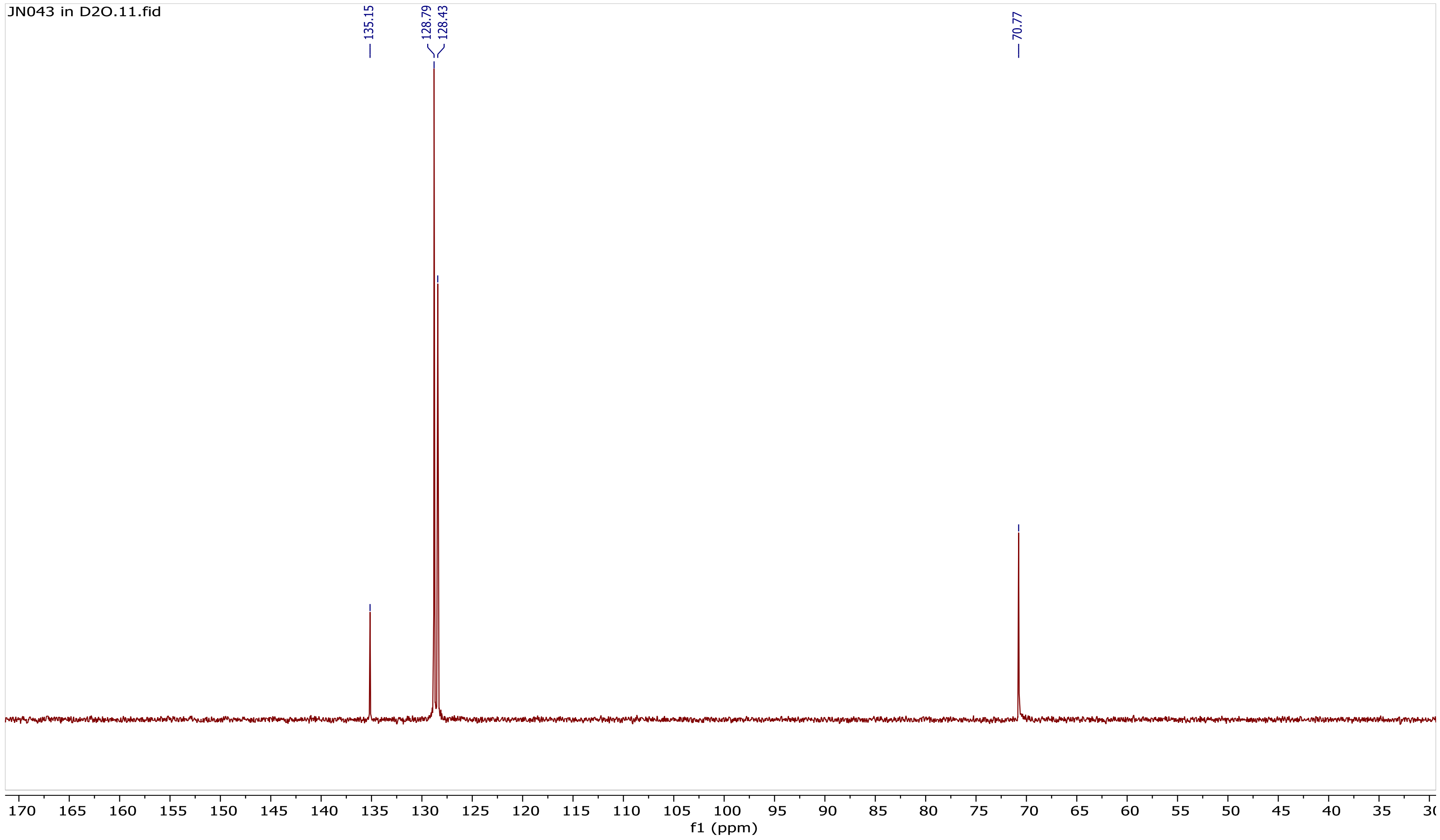

^{1}H NMR spectrum of **4b** (300 MHz, D_2O)

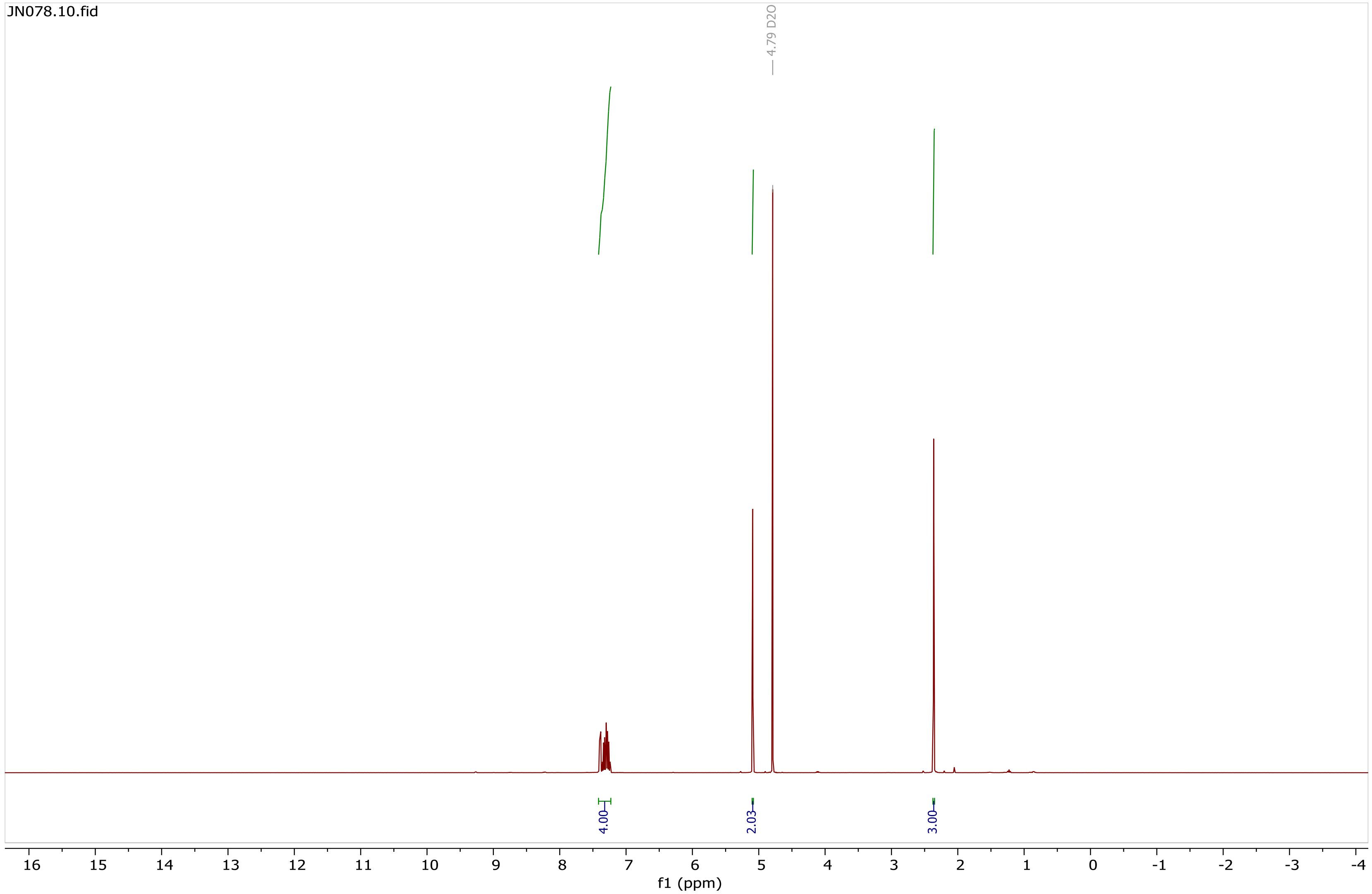

^{13}C NMR spectrum of **4b** (101 MHz, D_2O)

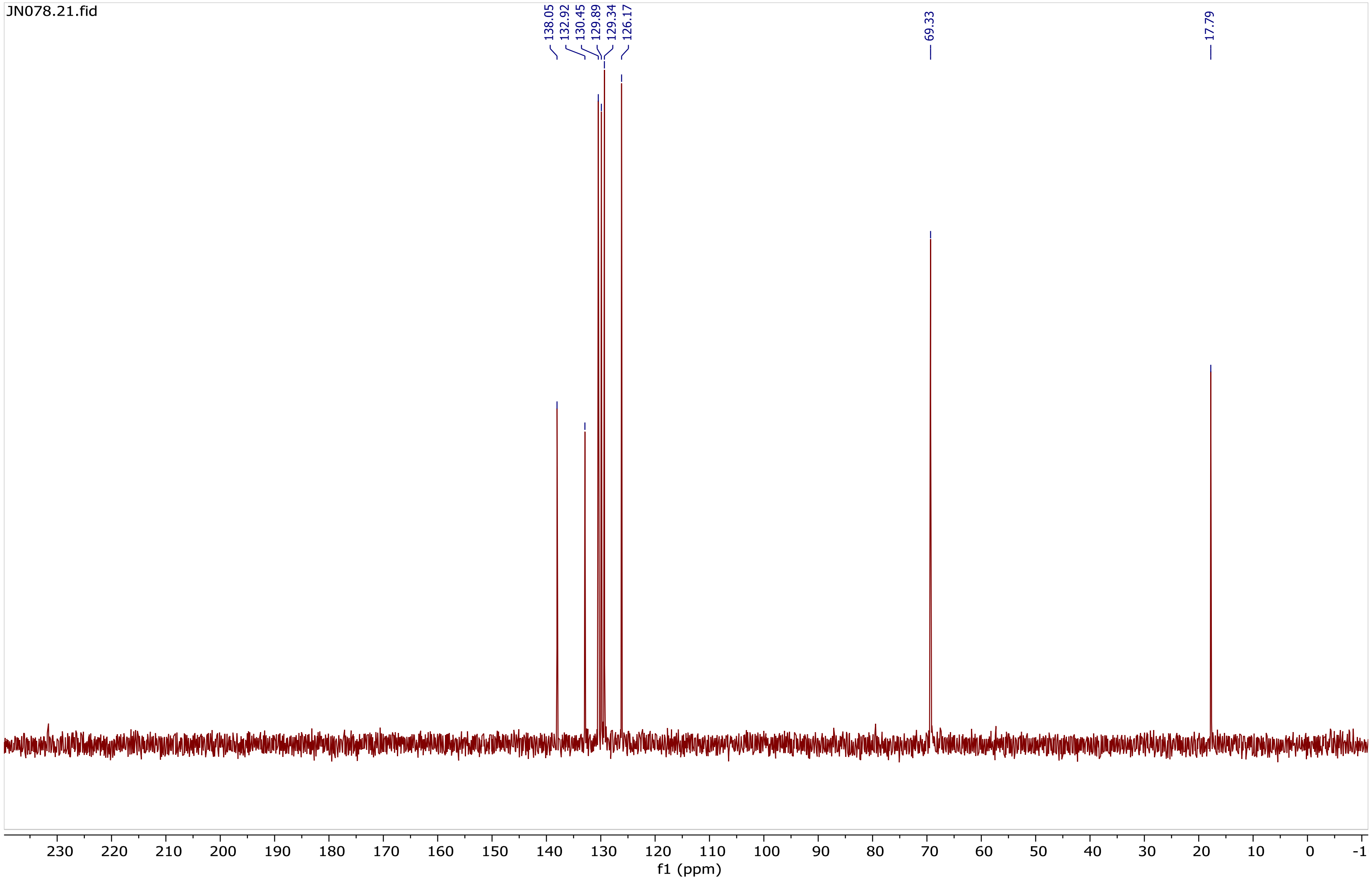

^{1}H NMR spectrum of **4c** (300 MHz, D_2O)

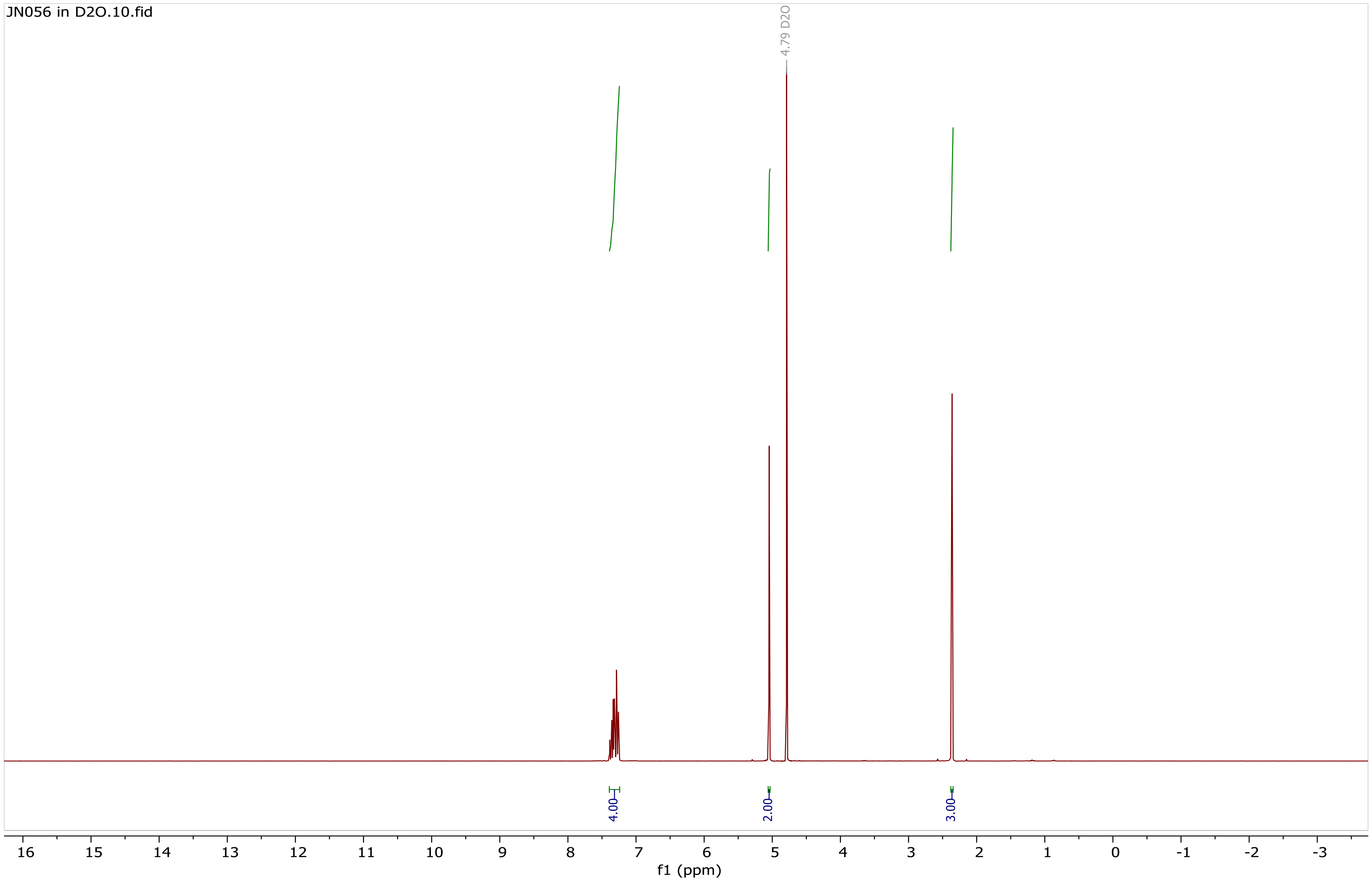

^{13}C NMR spectrum of **4c** (101 MHz, D_2O)

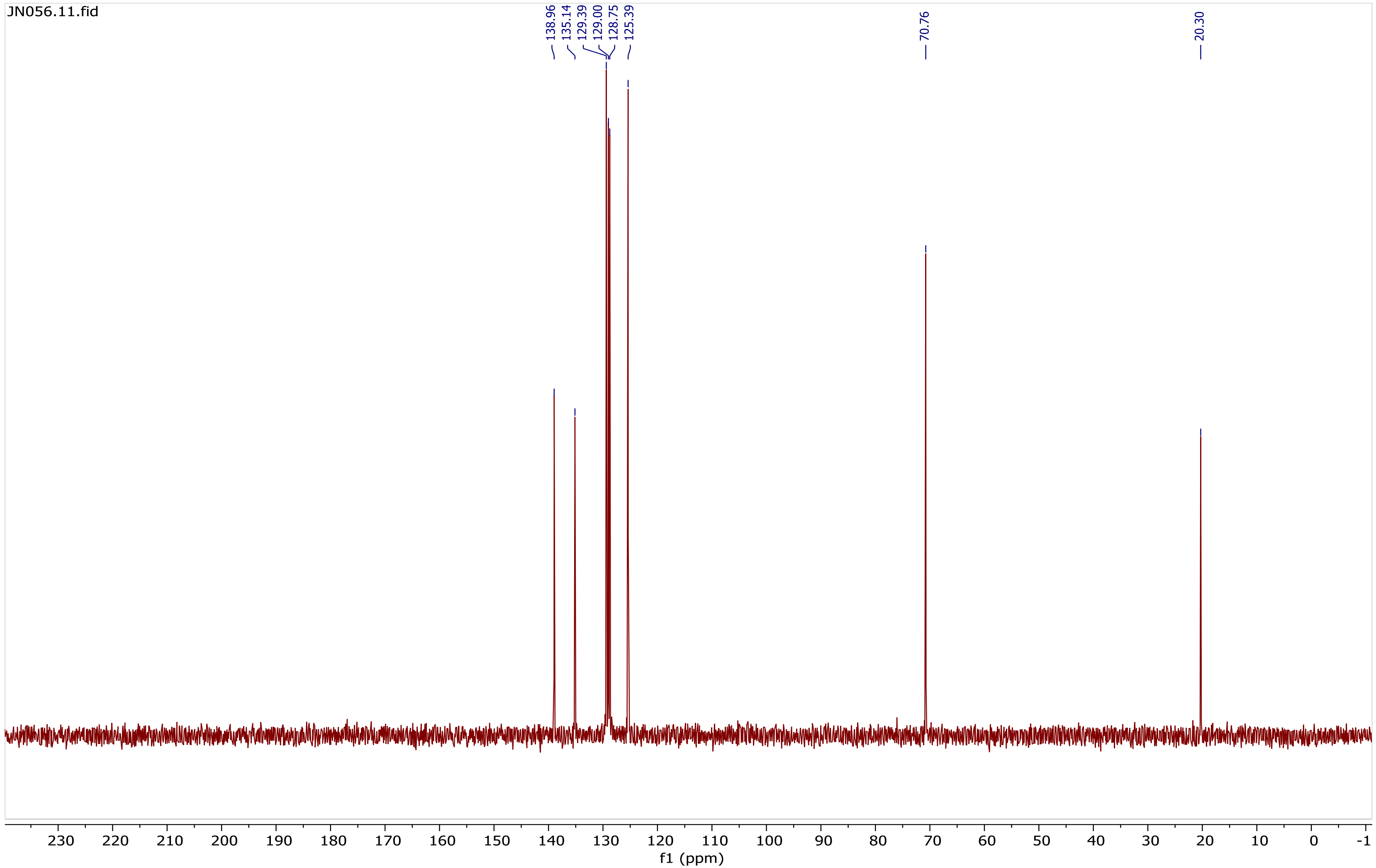

^{1}H NMR spectrum of **4d** (300 MHz, D_2O)

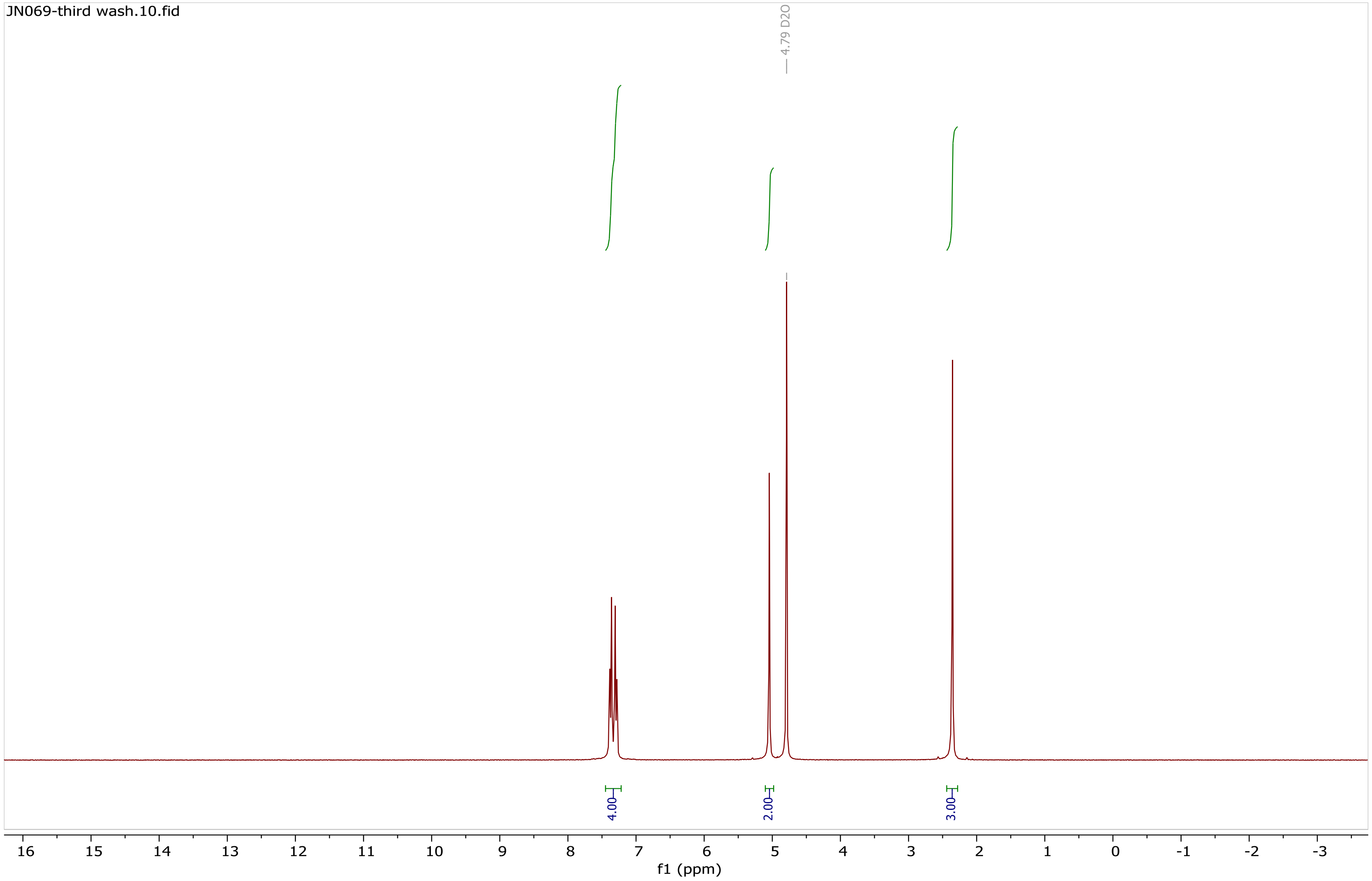

^{13}C NMR spectrum of **4d** (101 MHz, D_2O)

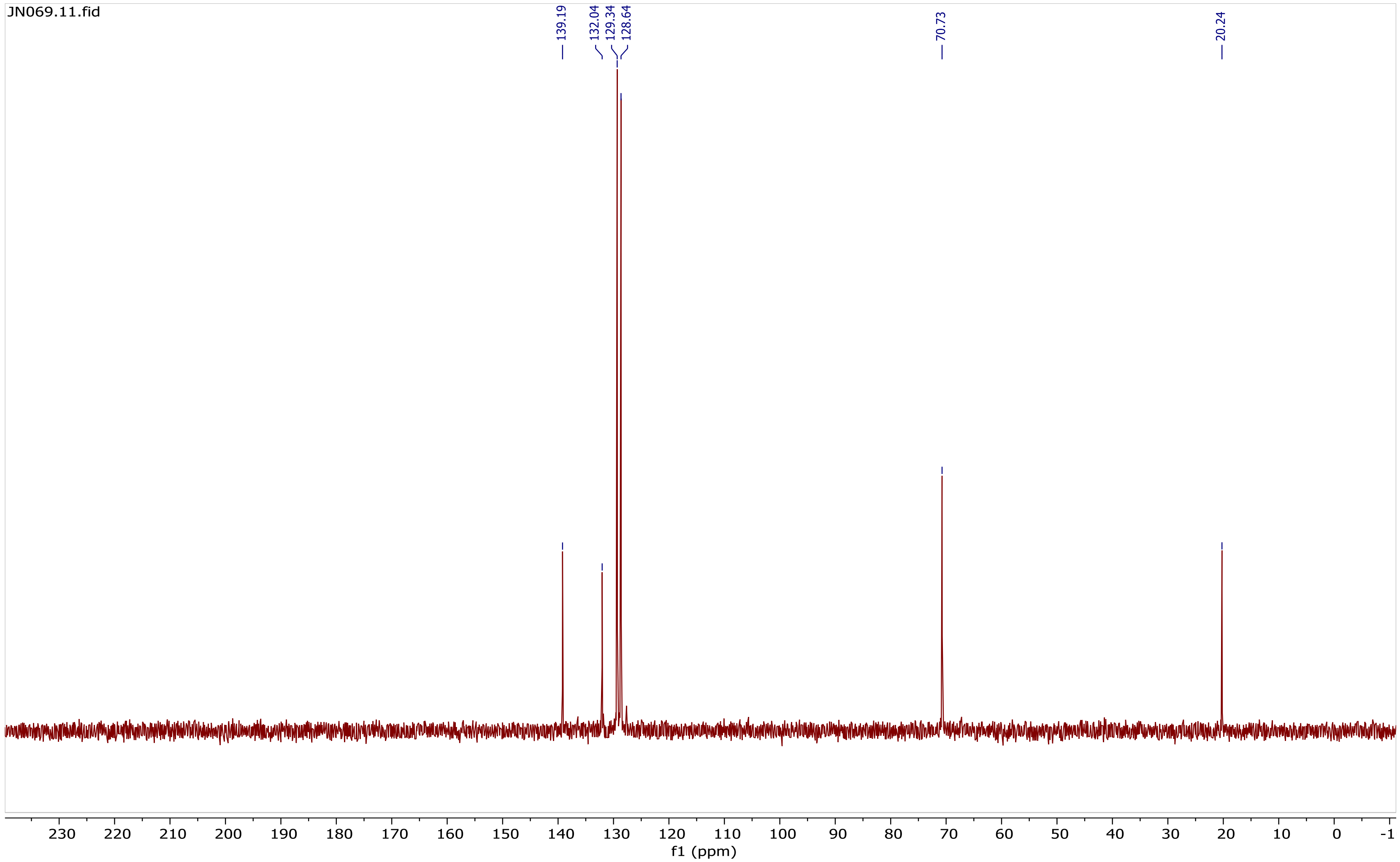

^{1}H NMR spectrum of **4e** (300 MHz, D_2O)

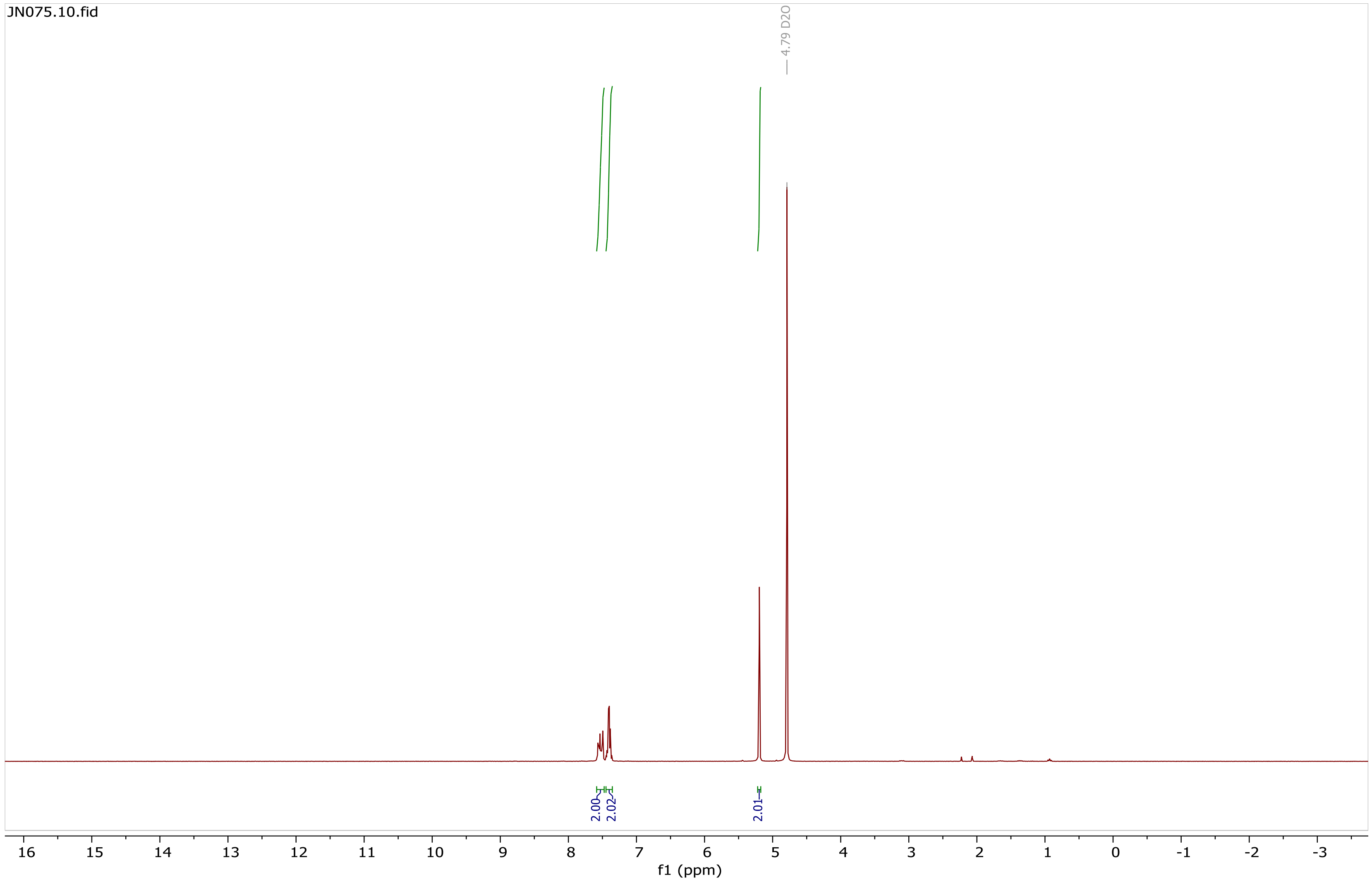

^{13}C NMR spectrum of **4e** (101 MHz, D_2O)

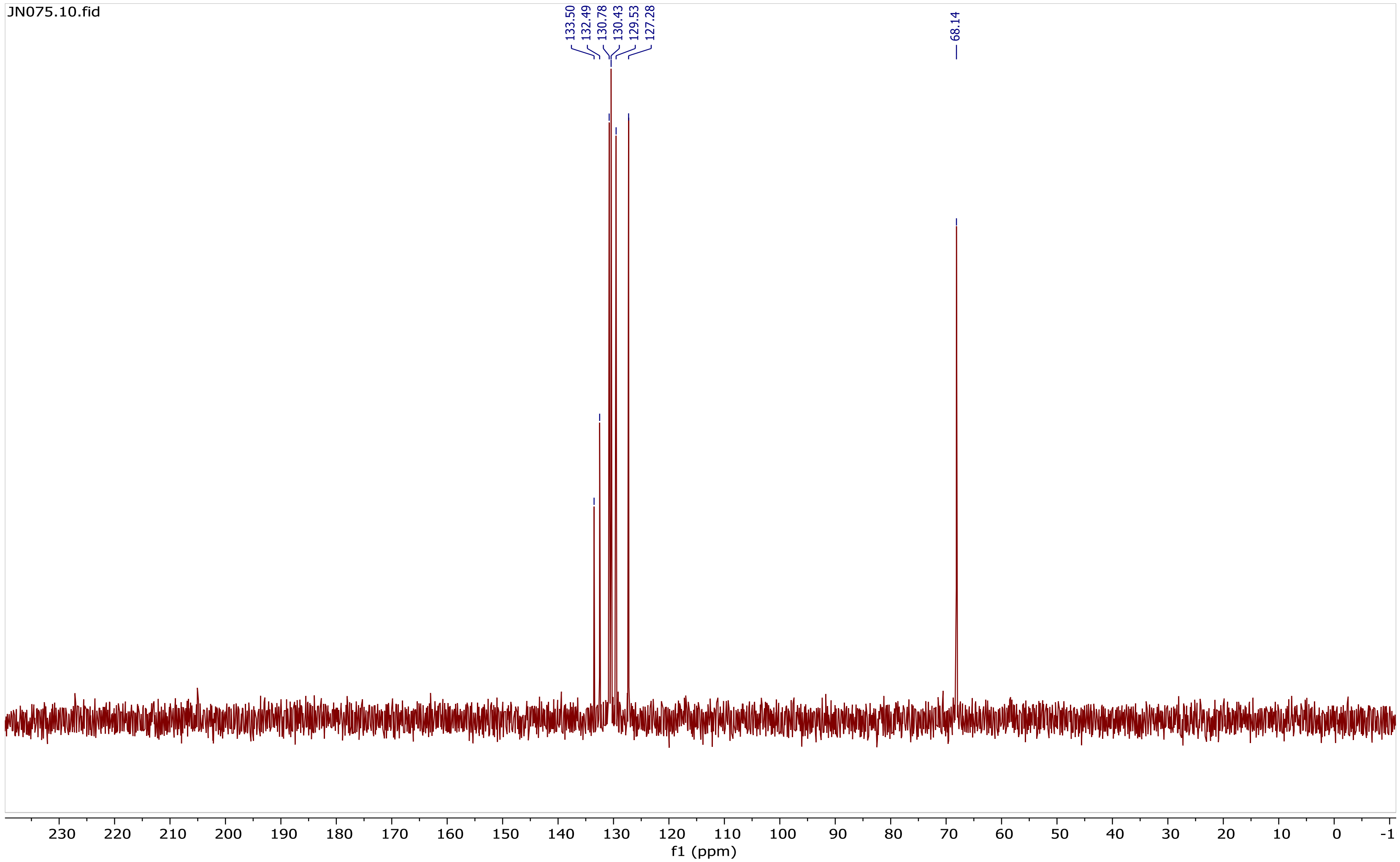

^{1}H NMR spectrum of **4f** (300 MHz, D_2O)

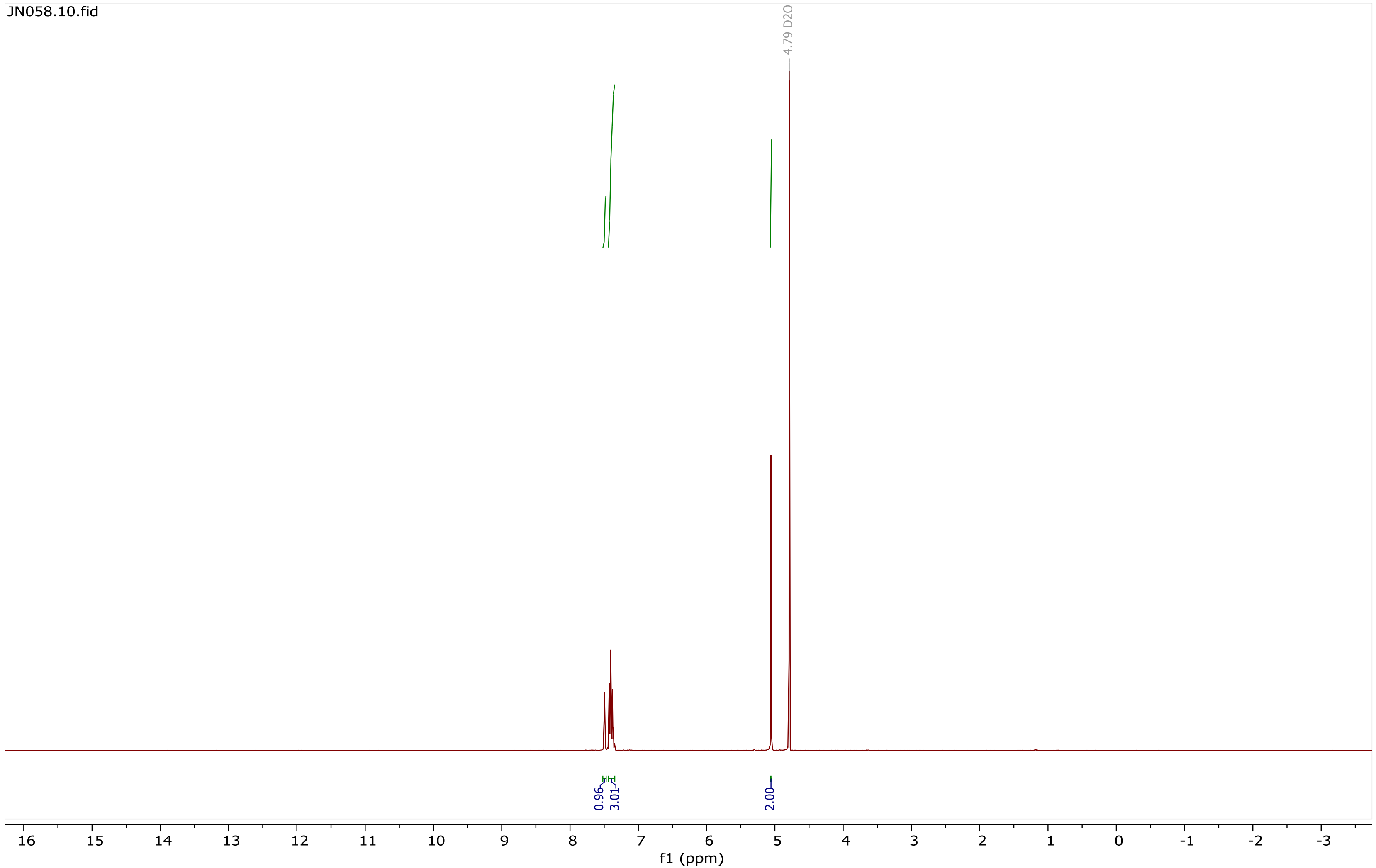

^{13}C NMR spectrum of **4f** (101 MHz, D_2O)

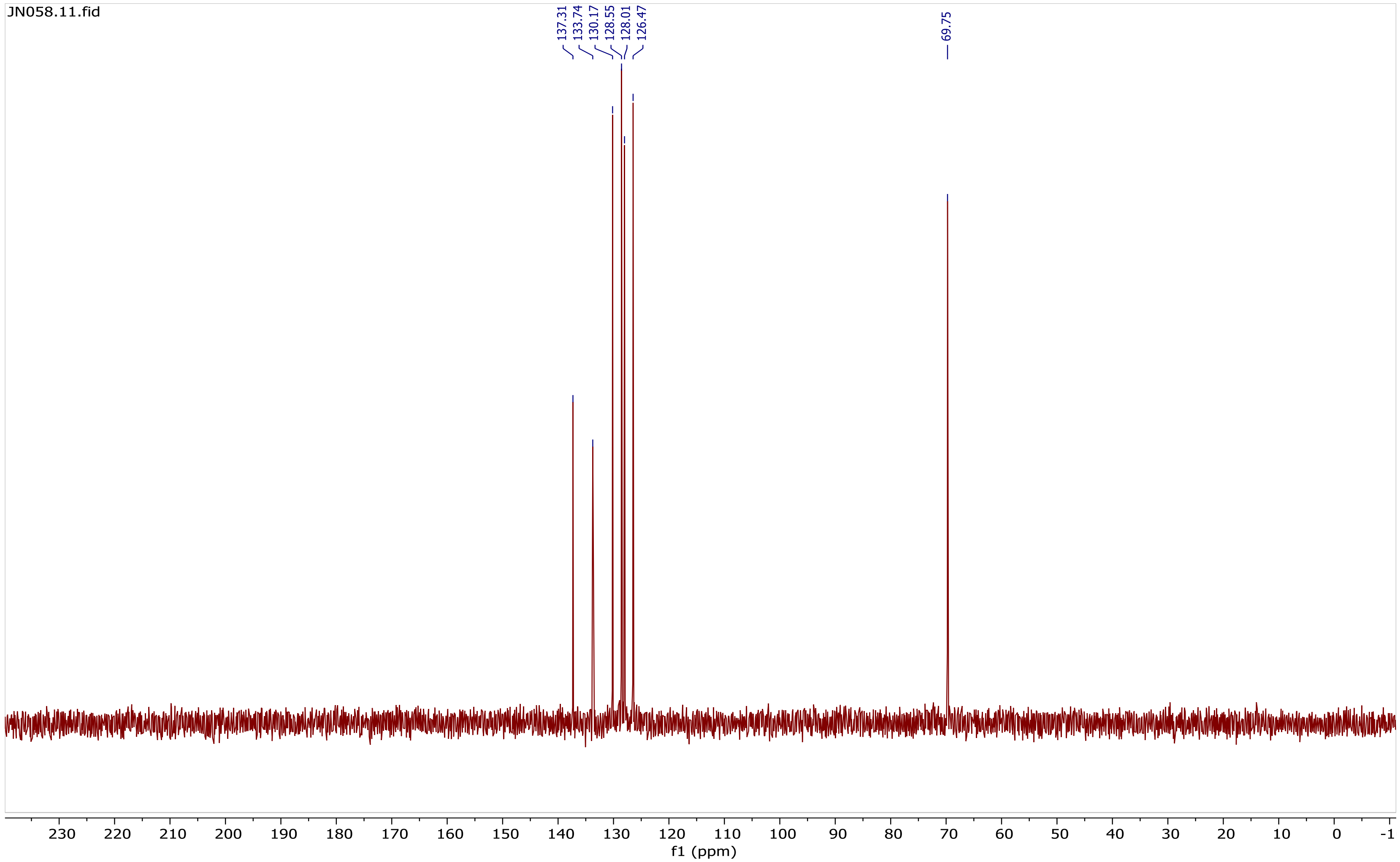

^{1}H NMR spectrum of **4g** (300 MHz, D_2O)

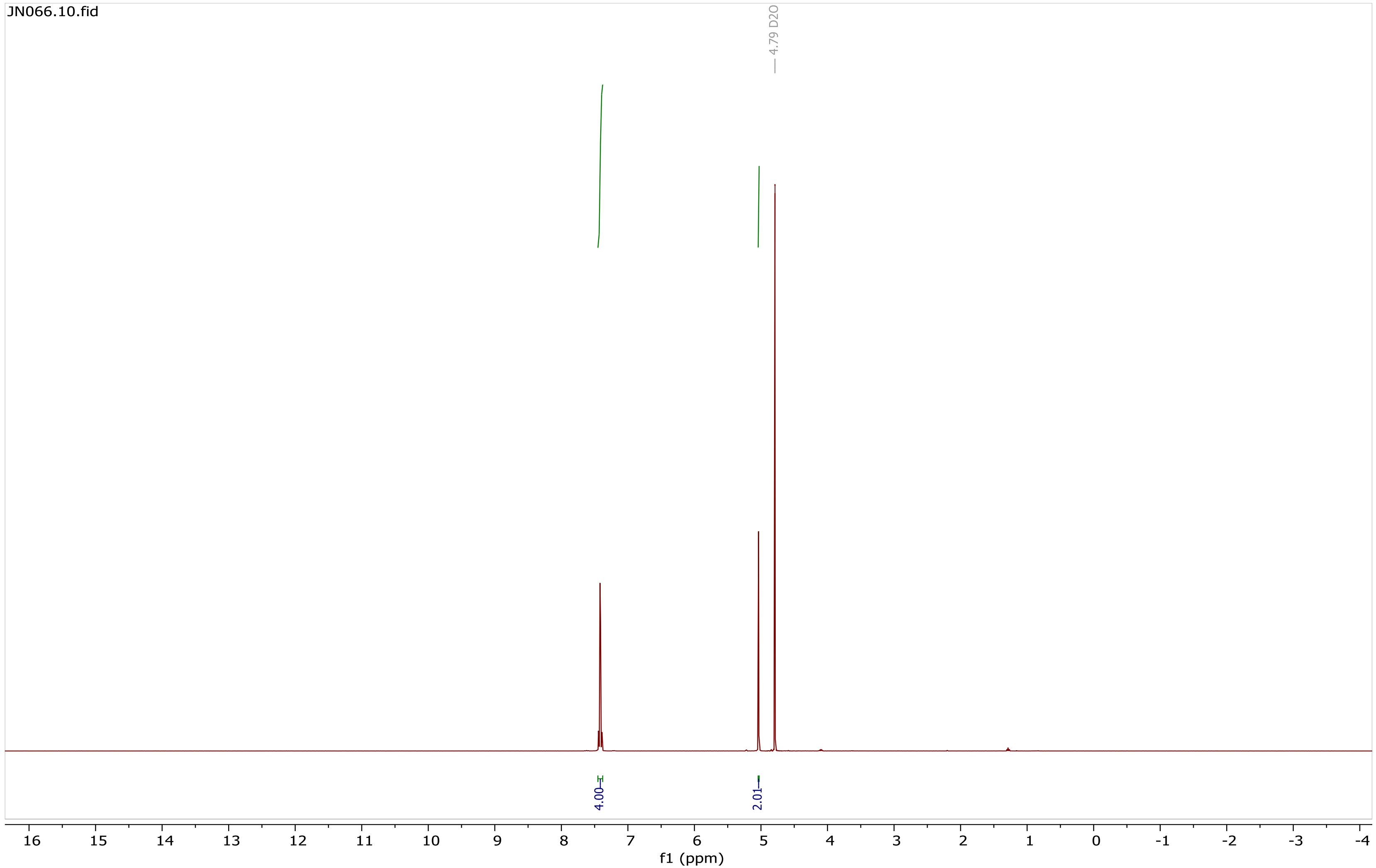

^{13}C NMR spectrum of **4g** (101 MHz, D_2O)

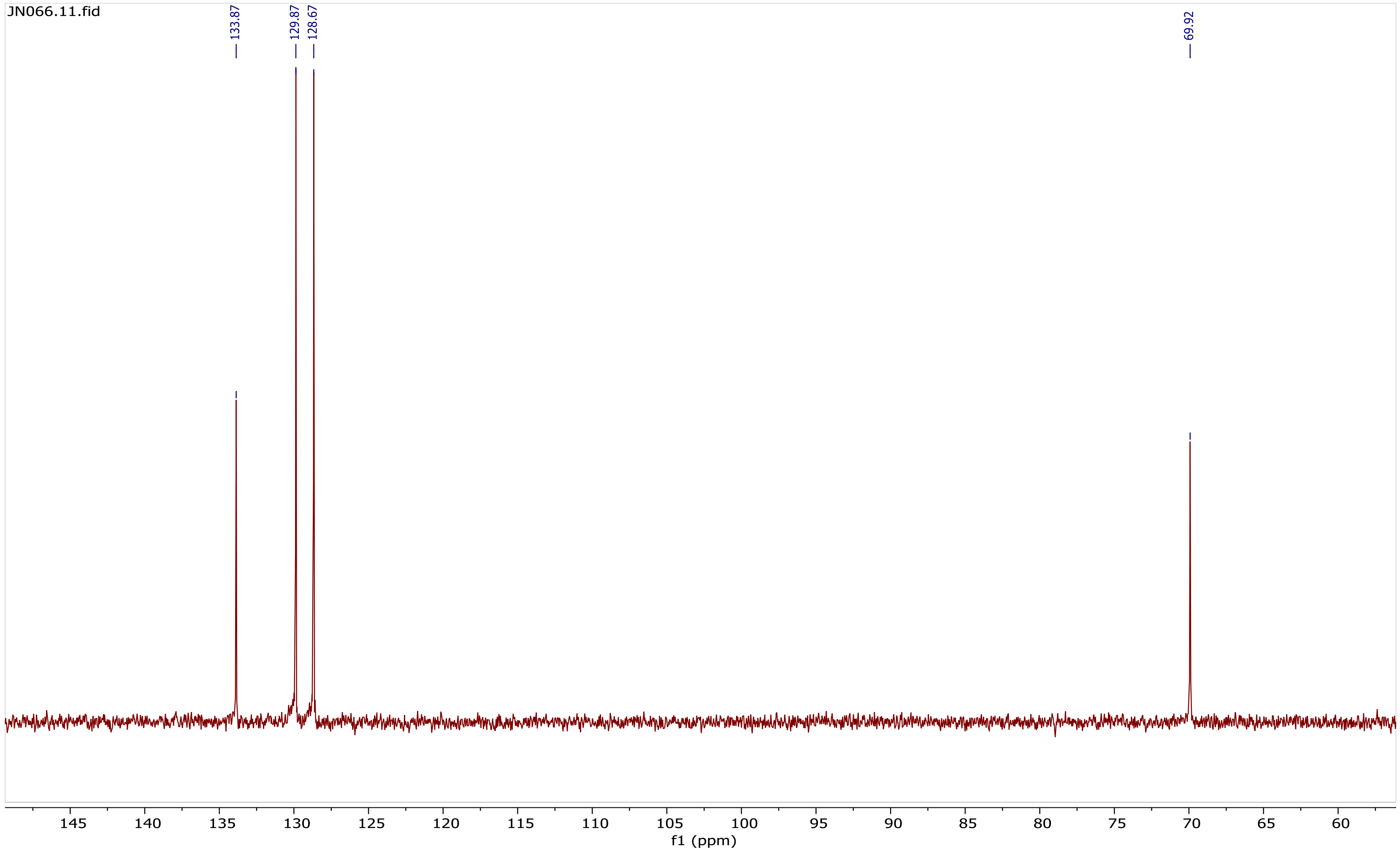

^{1}H NMR spectrum of **4h** (300 MHz, D_2O)

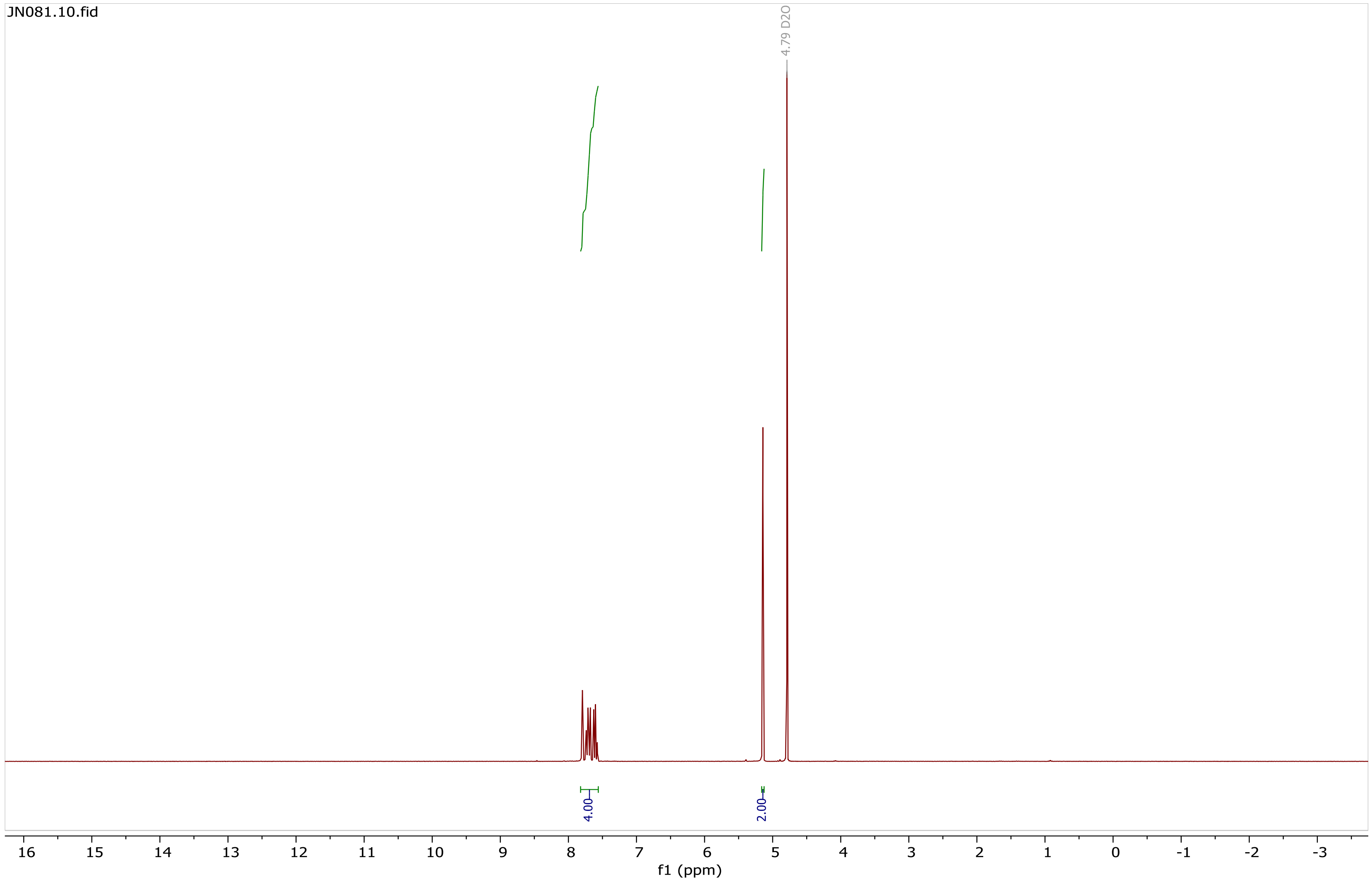

^{13}C NMR spectrum of **4h** (101 MHz, D_2O)

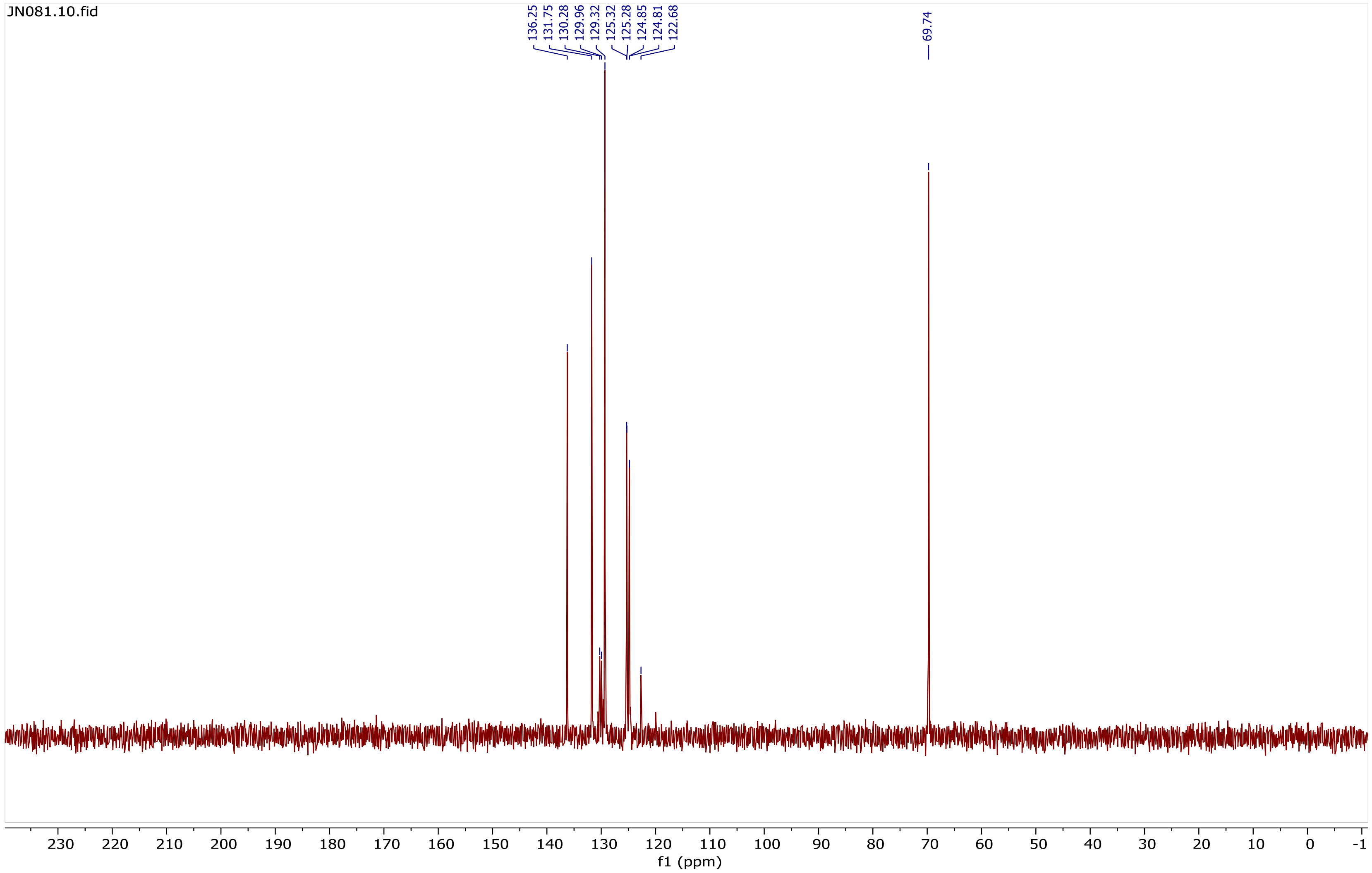

^{19}F NMR spectrum of **4h** (377 MHz, D_2O)

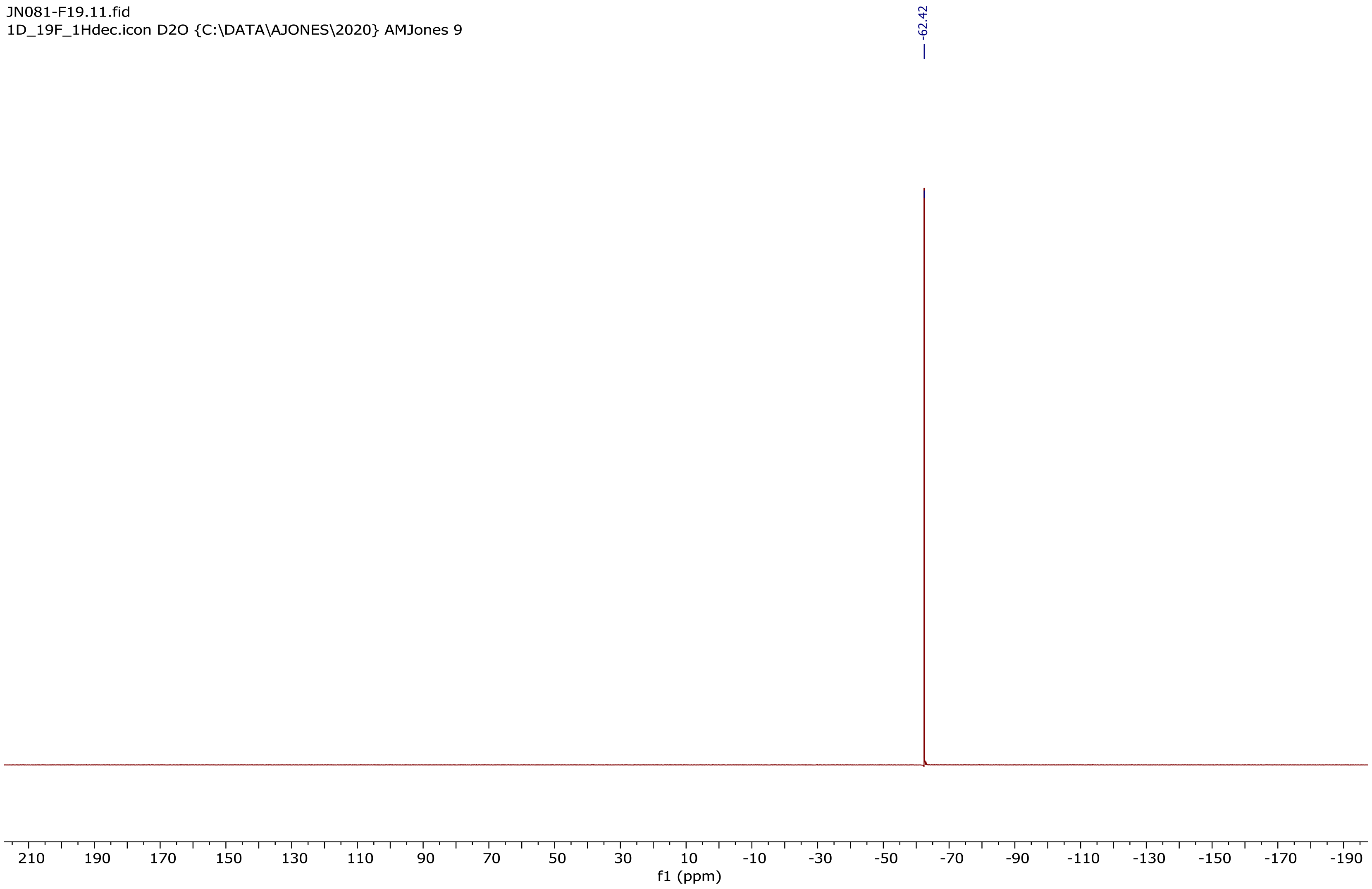

^{1}H NMR spectrum of **4i** (300 MHz, D_2O)

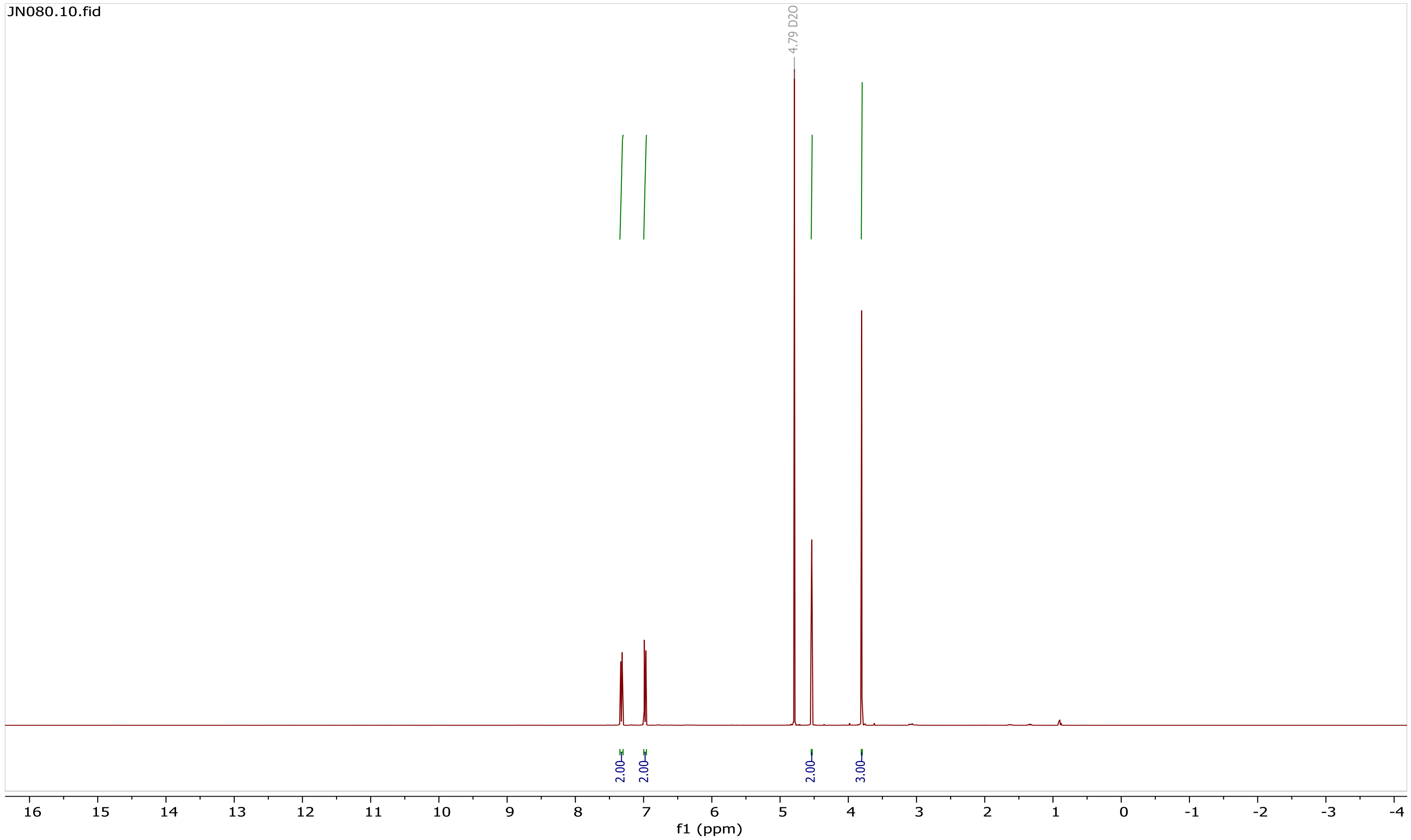

^{13}C NMR spectrum of **4i** (101 MHz, D_2O)

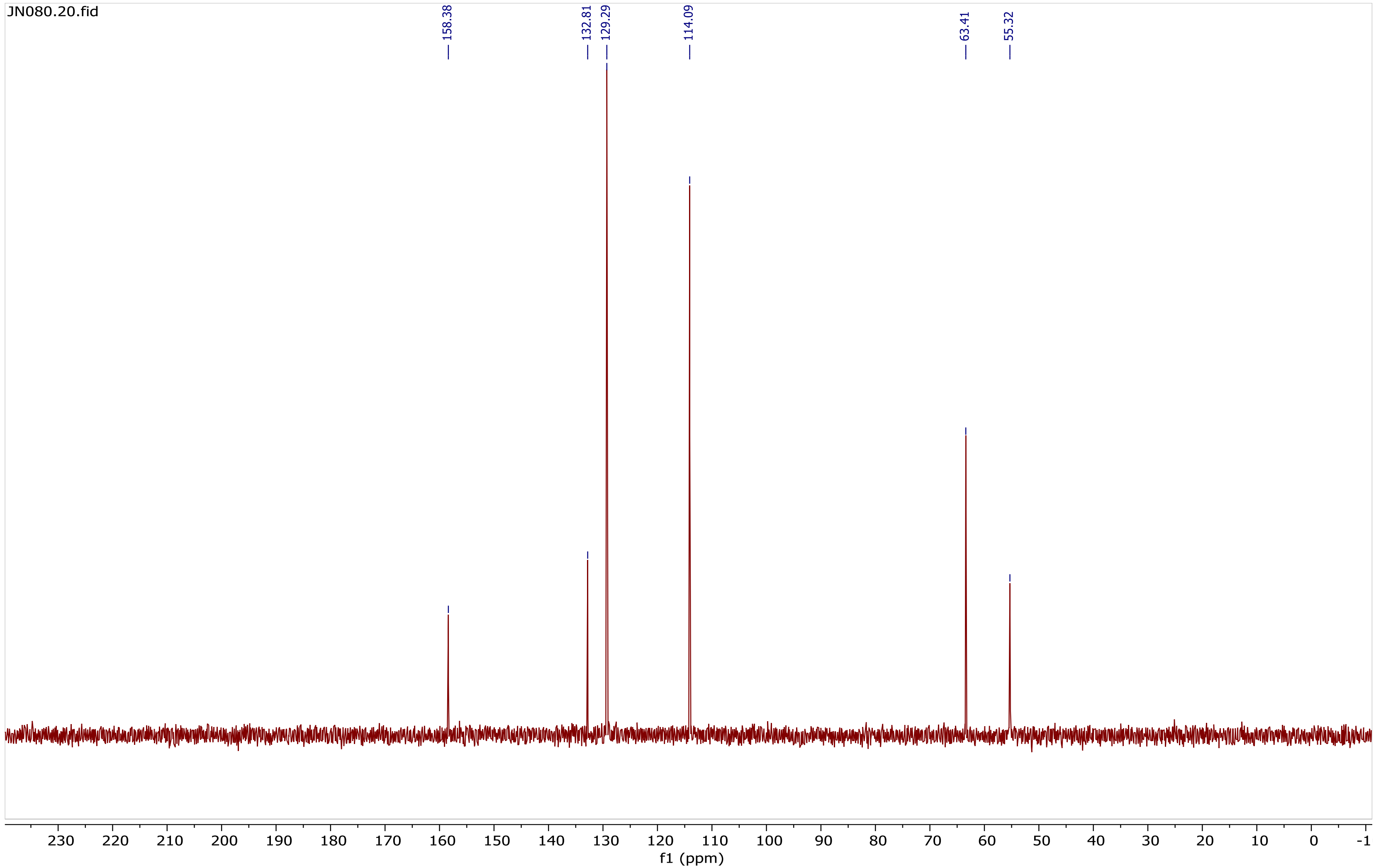

^1H NMR spectrum of **4j** (300 MHz, D_2O)

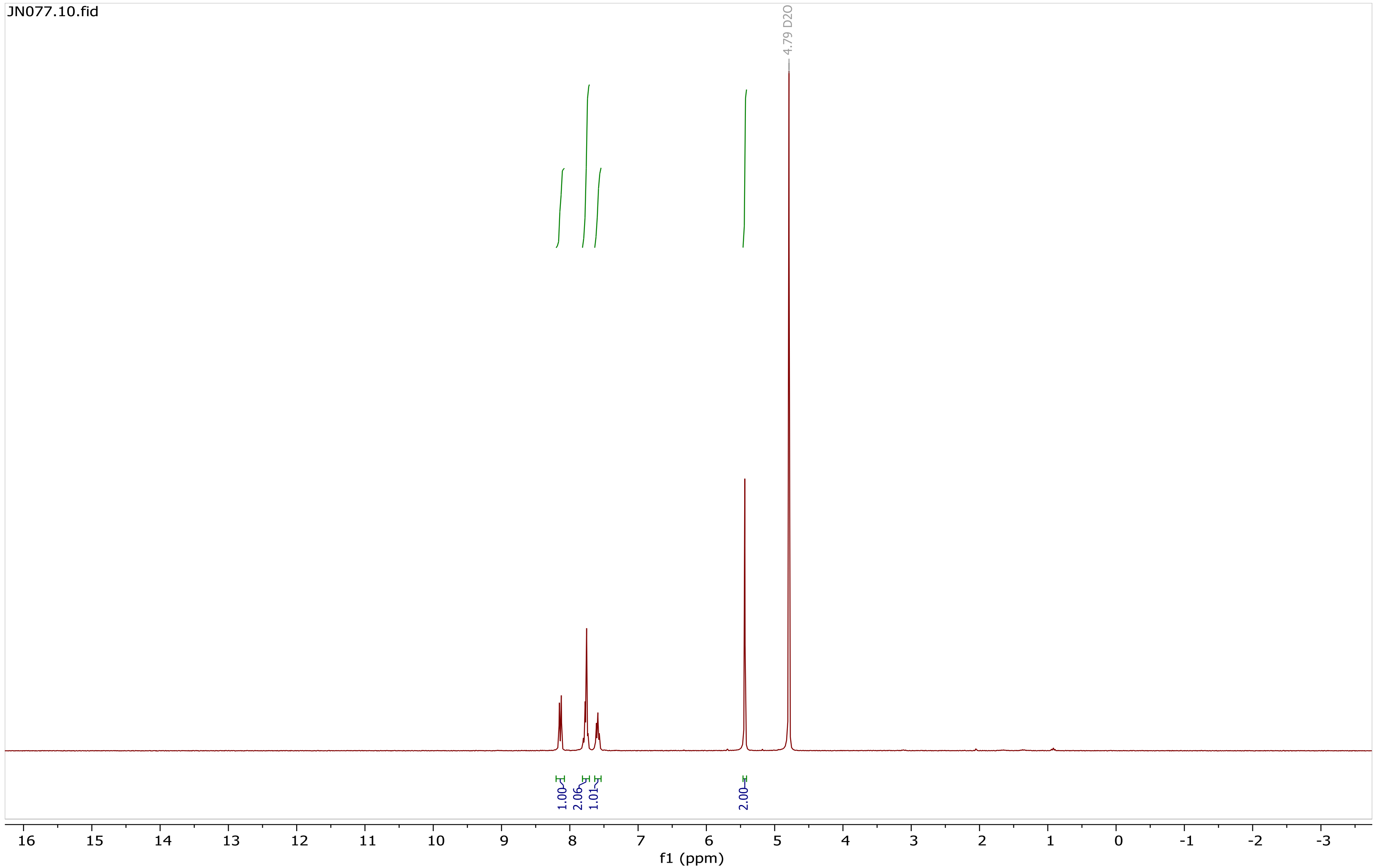

^{13}C NMR spectrum of **4j** (101 MHz, D_2O)

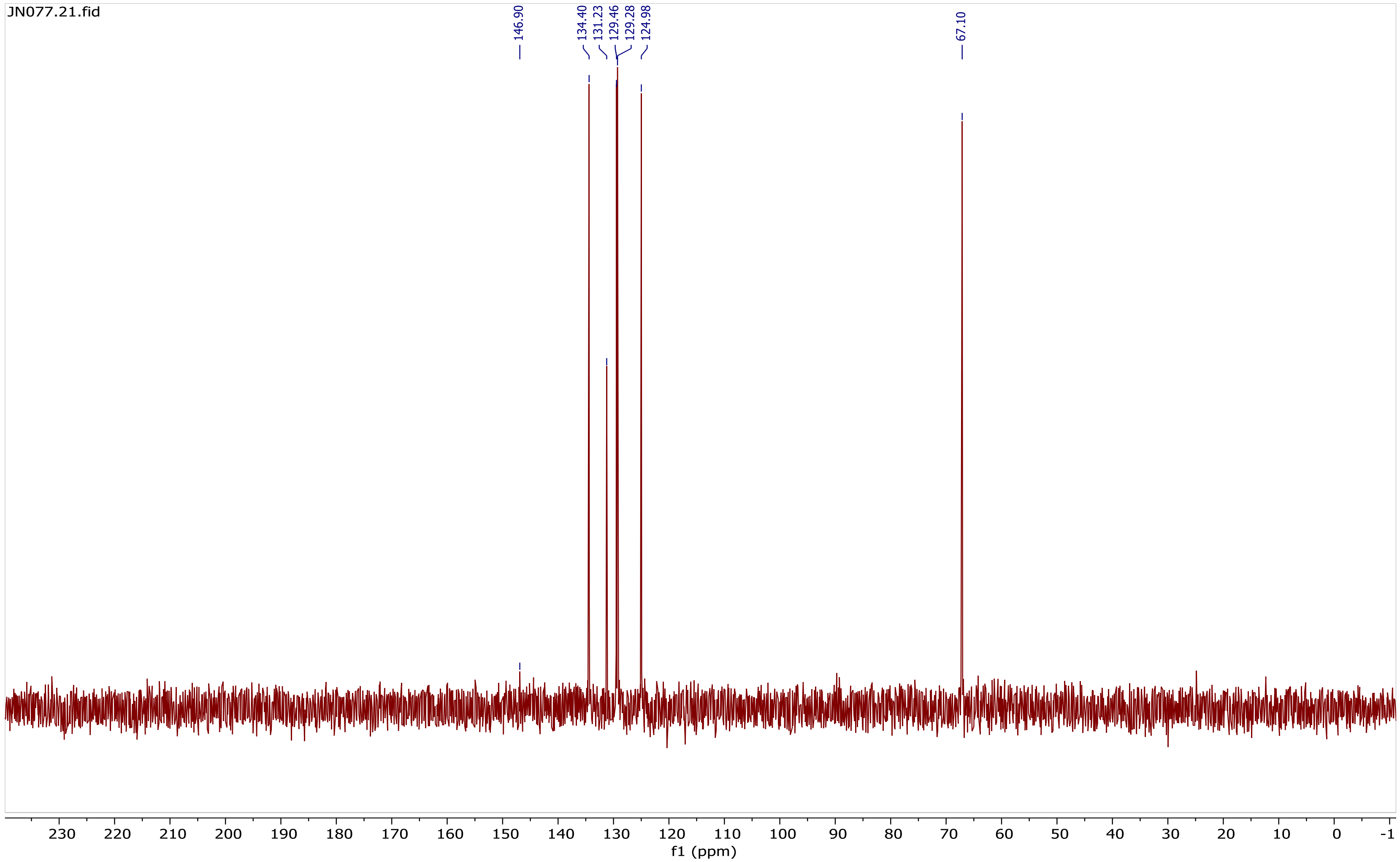

^{1}H NMR spectrum of **4k** (300 MHz, D_2O)

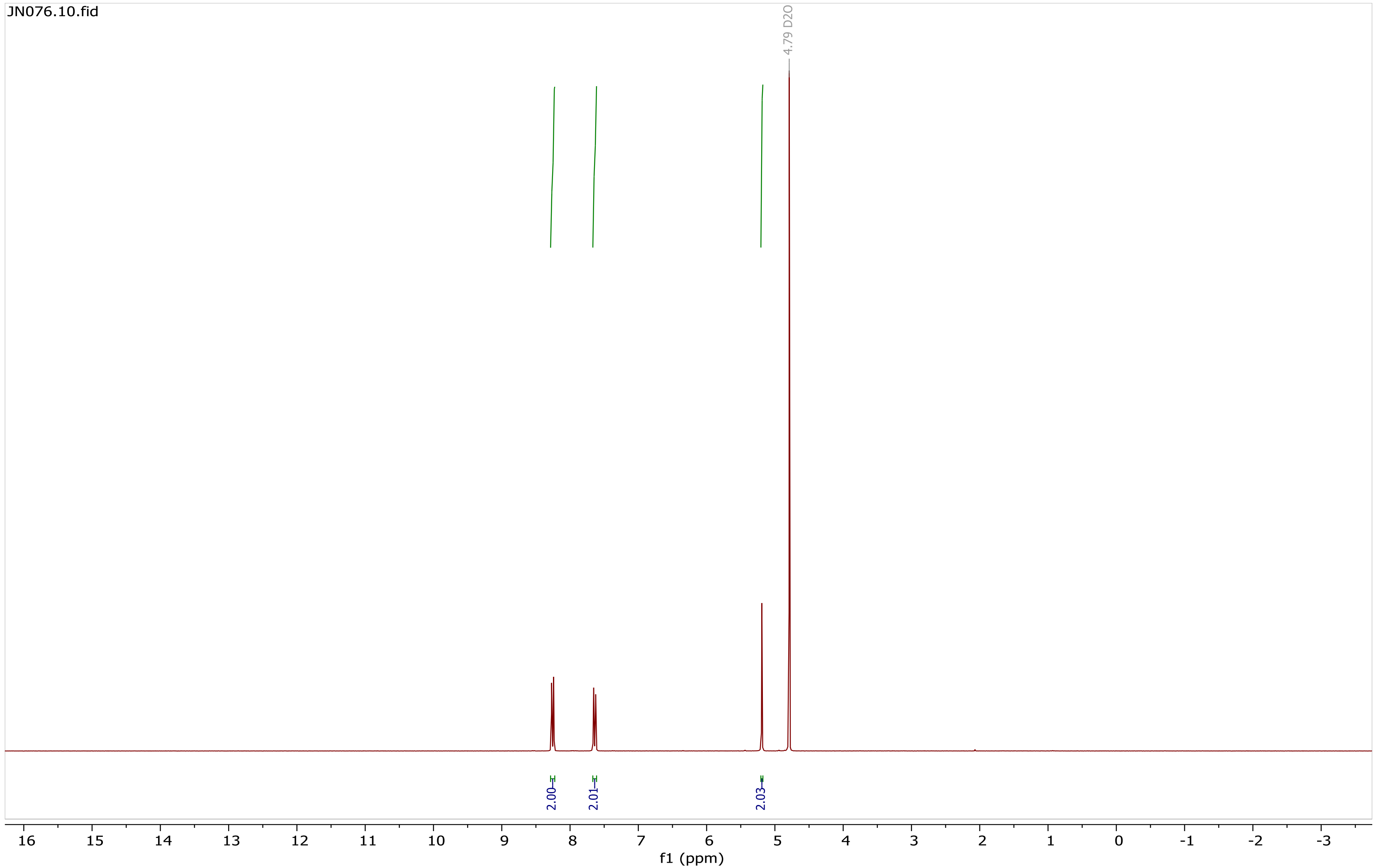

^{13}C NMR spectrum of **4k** (101 MHz, D_2O)

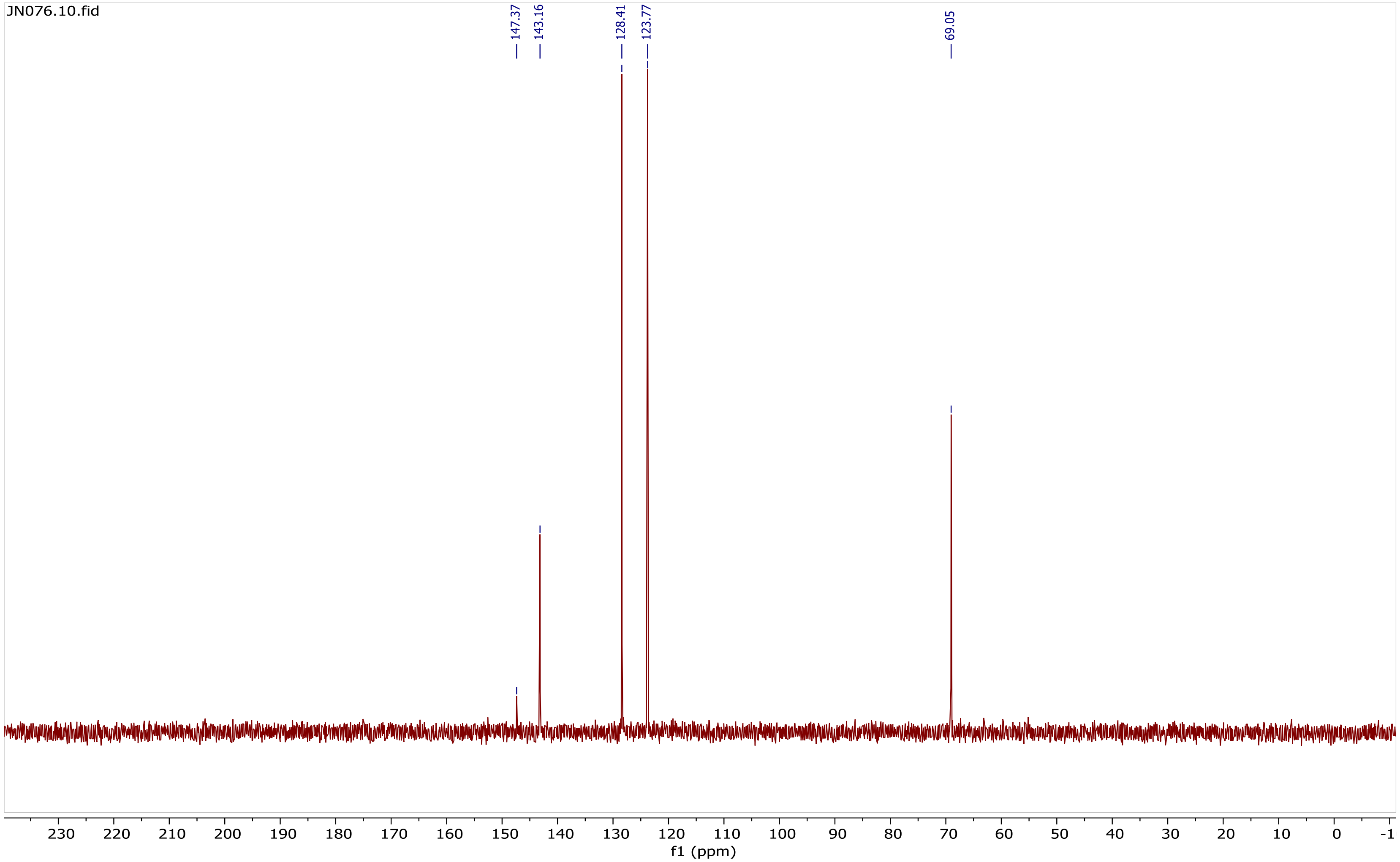

^{1}H NMR spectrum of **6a** (400 MHz, $CDCl_3$)

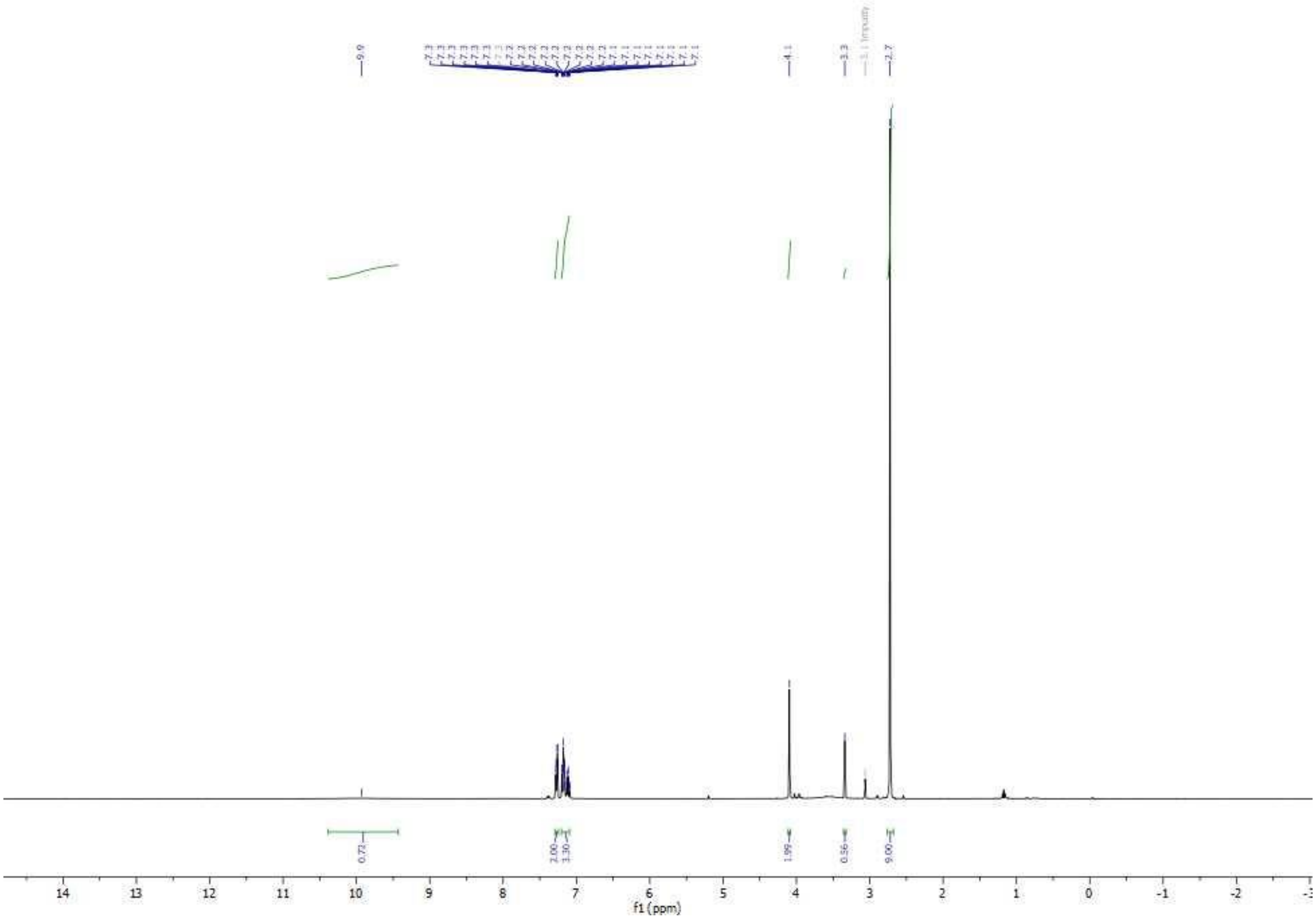

^{13}C NMR spectrum of **6a** (101 MHz, $CDCl_3$)

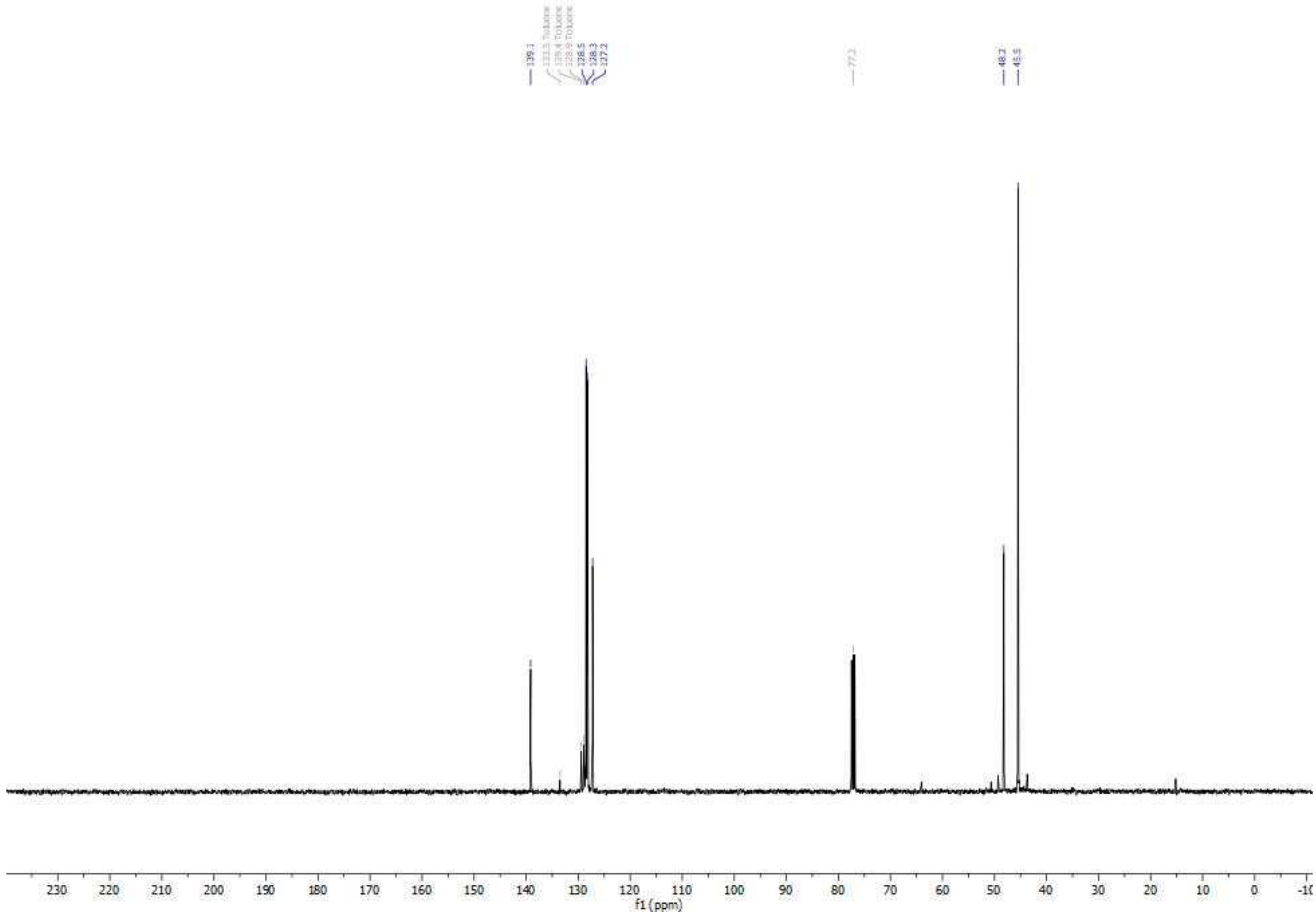

^{1}H NMR spectrum of **7a** (400 MHz, d_6-DMSO)

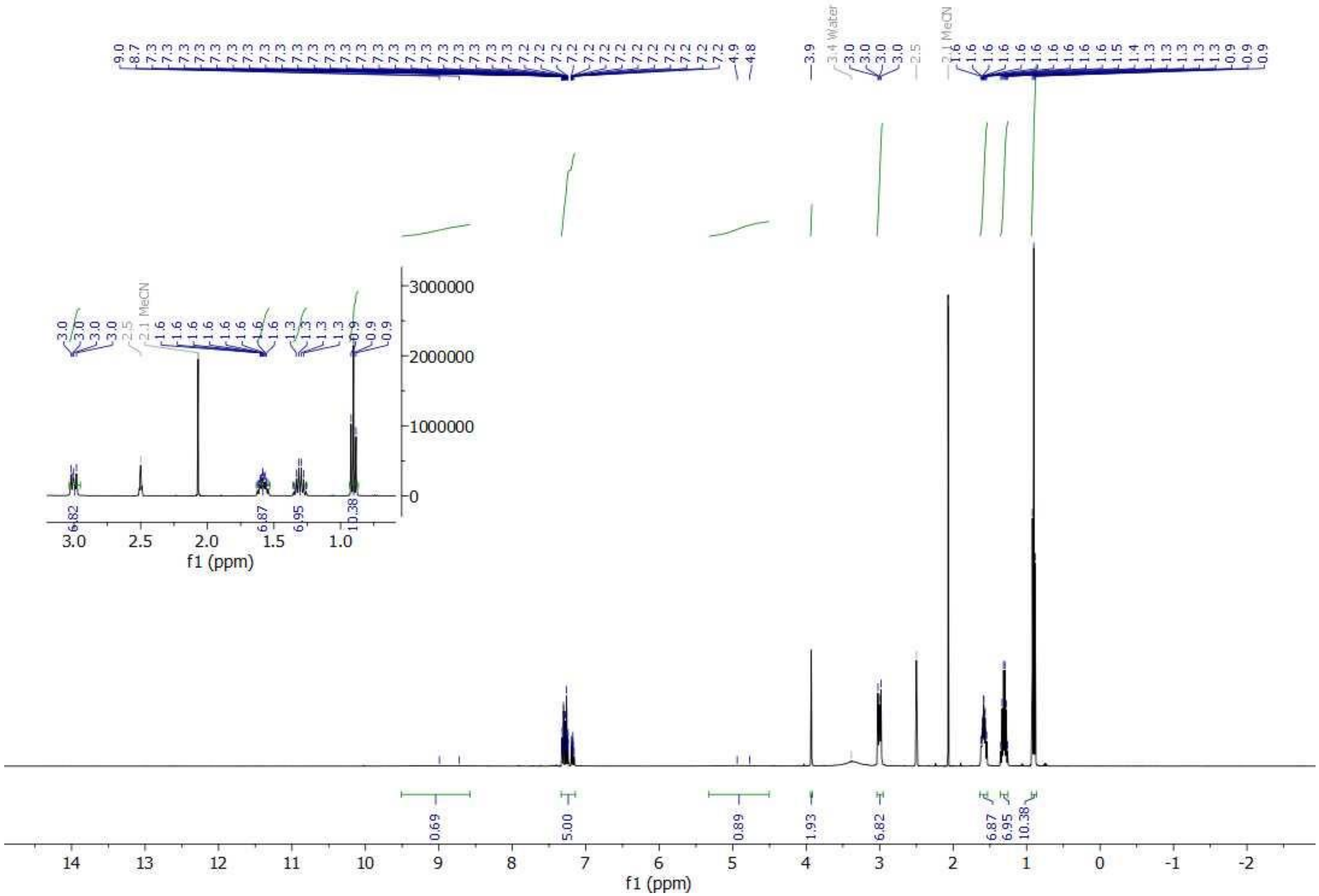

^{13}C NMR spectrum of **7a** (101 MHz, d_6-DMSO)

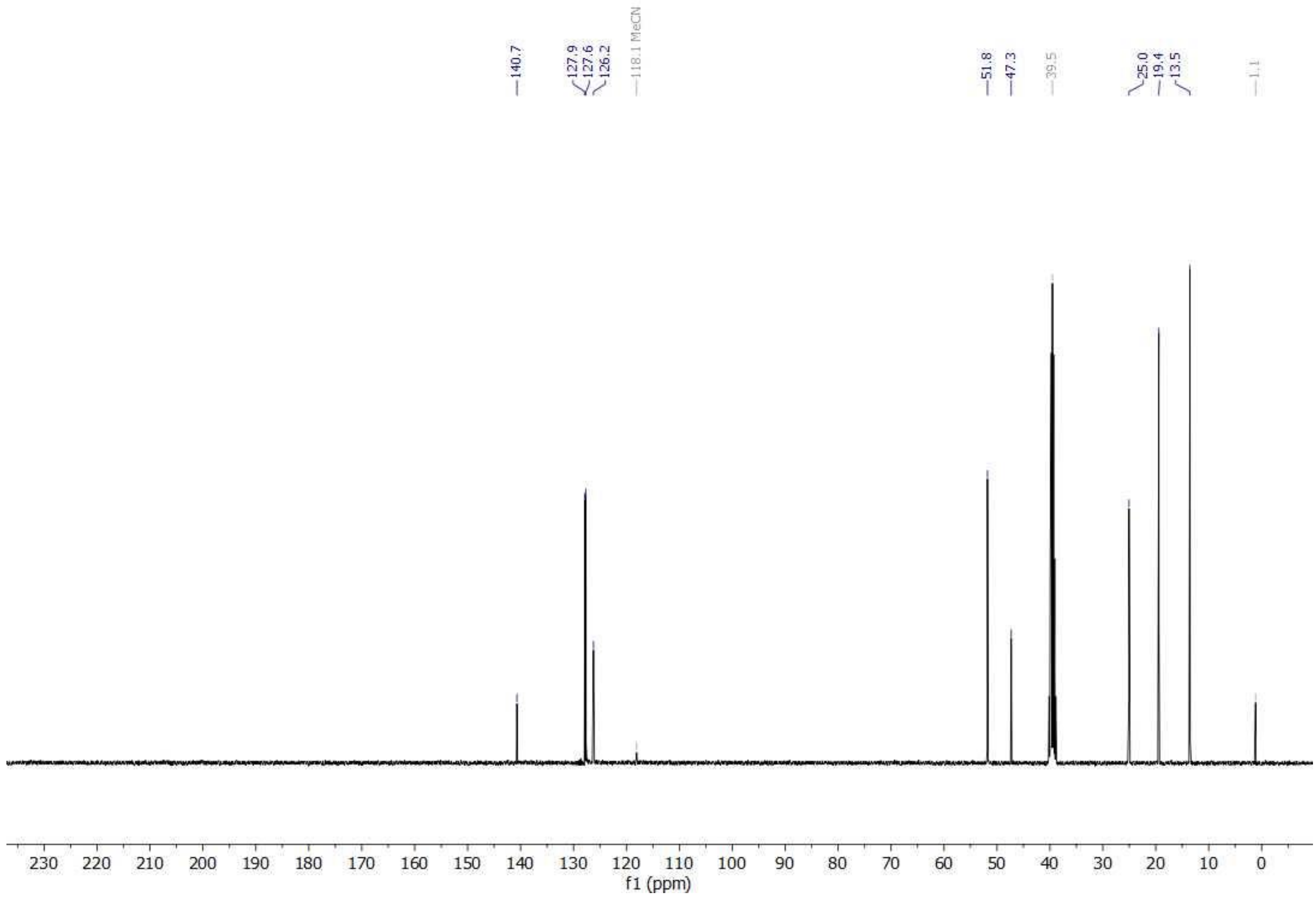

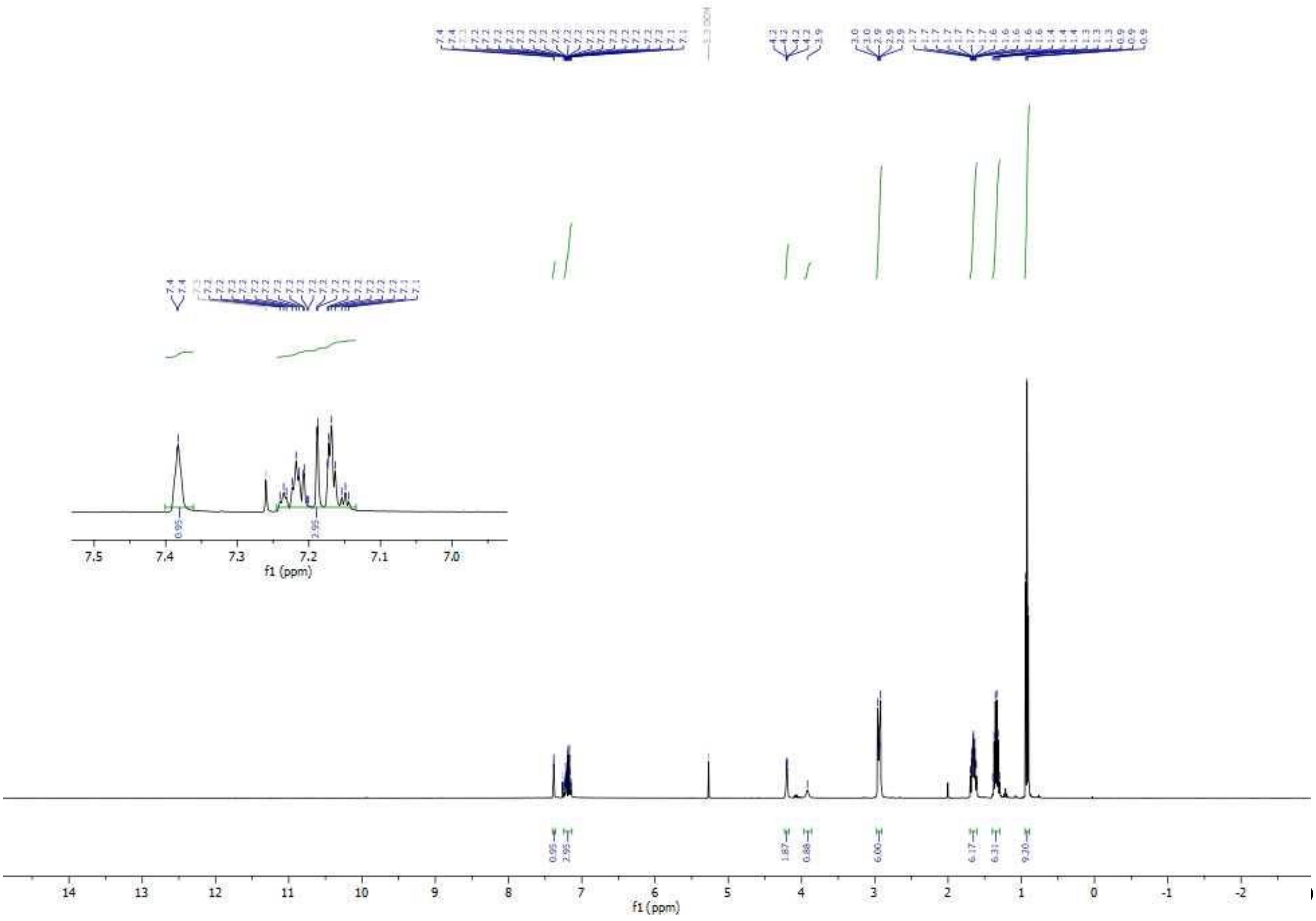

^{1}H NMR spectrum of **7e** (400 MHz, $CDCl_3$)

^{13}C NMR spectrum of **7e** (101 MHz, $CDCl_3$)

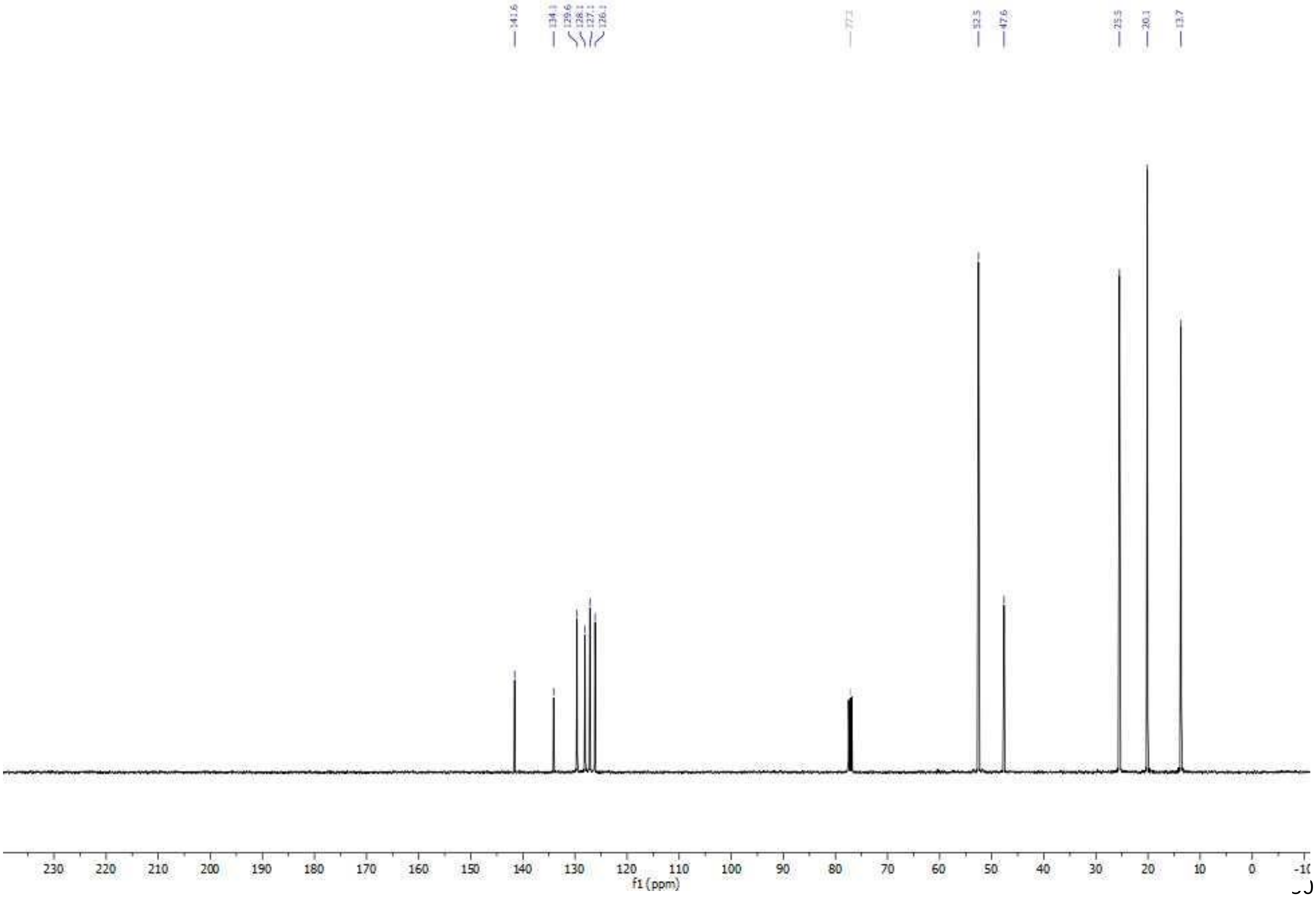

^{1}H NMR spectrum of **7f** (400 MHz, $CDCl_3$)

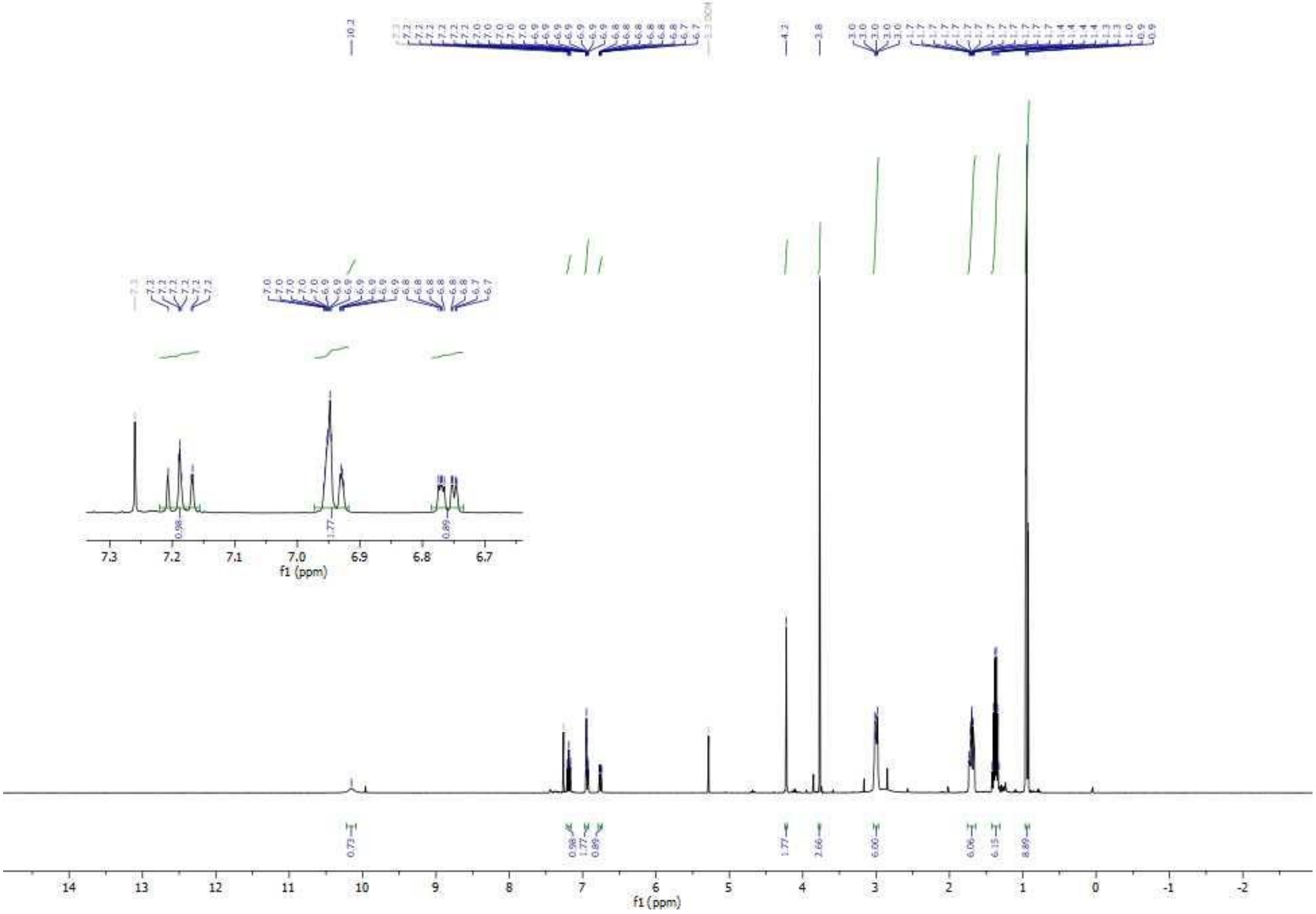

^{13}C NMR spectrum of **7f** (101 MHz, $CDCl_3$)

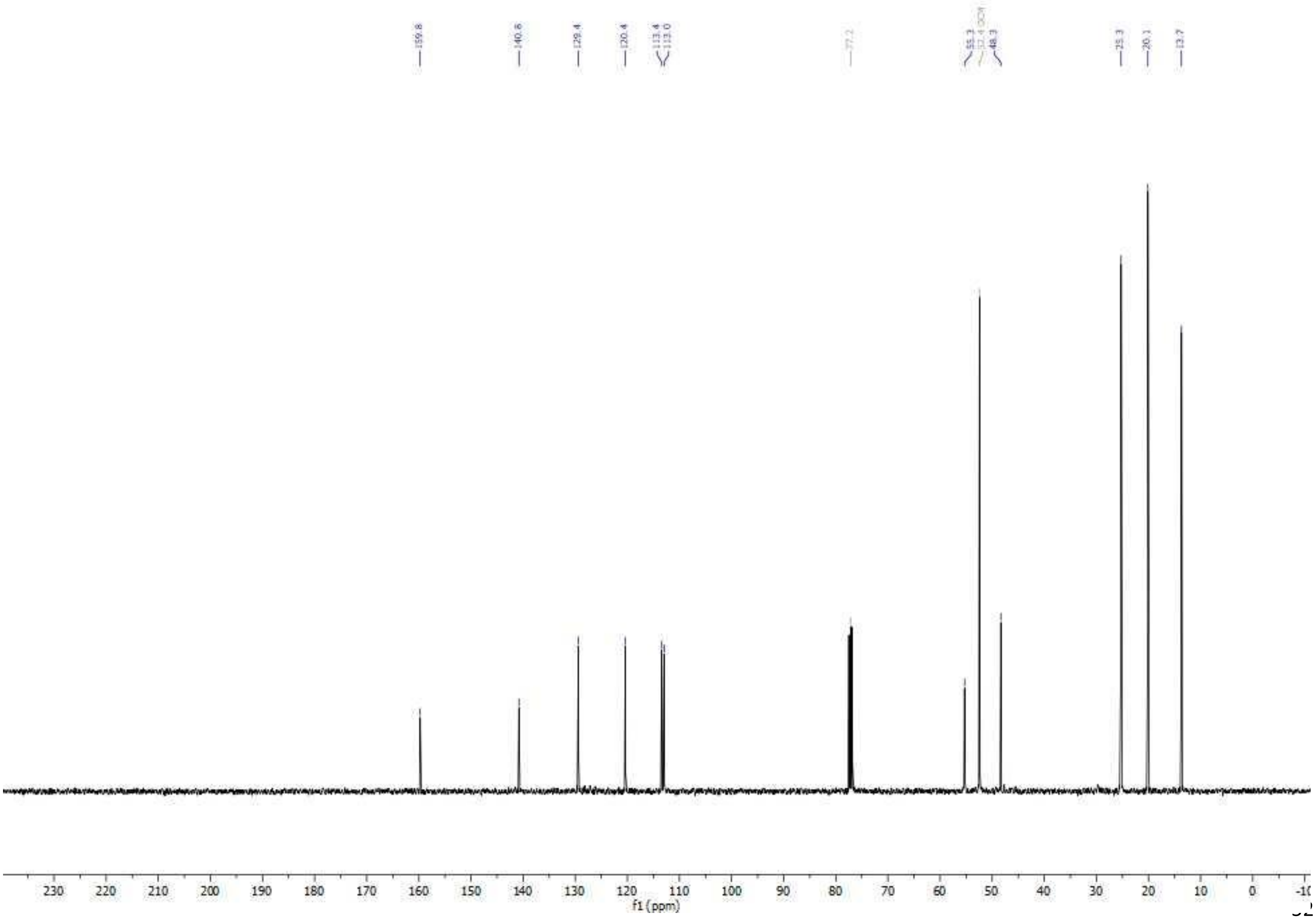

^{1}H NMR spectrum of **8a** (300 MHz, D_2O)

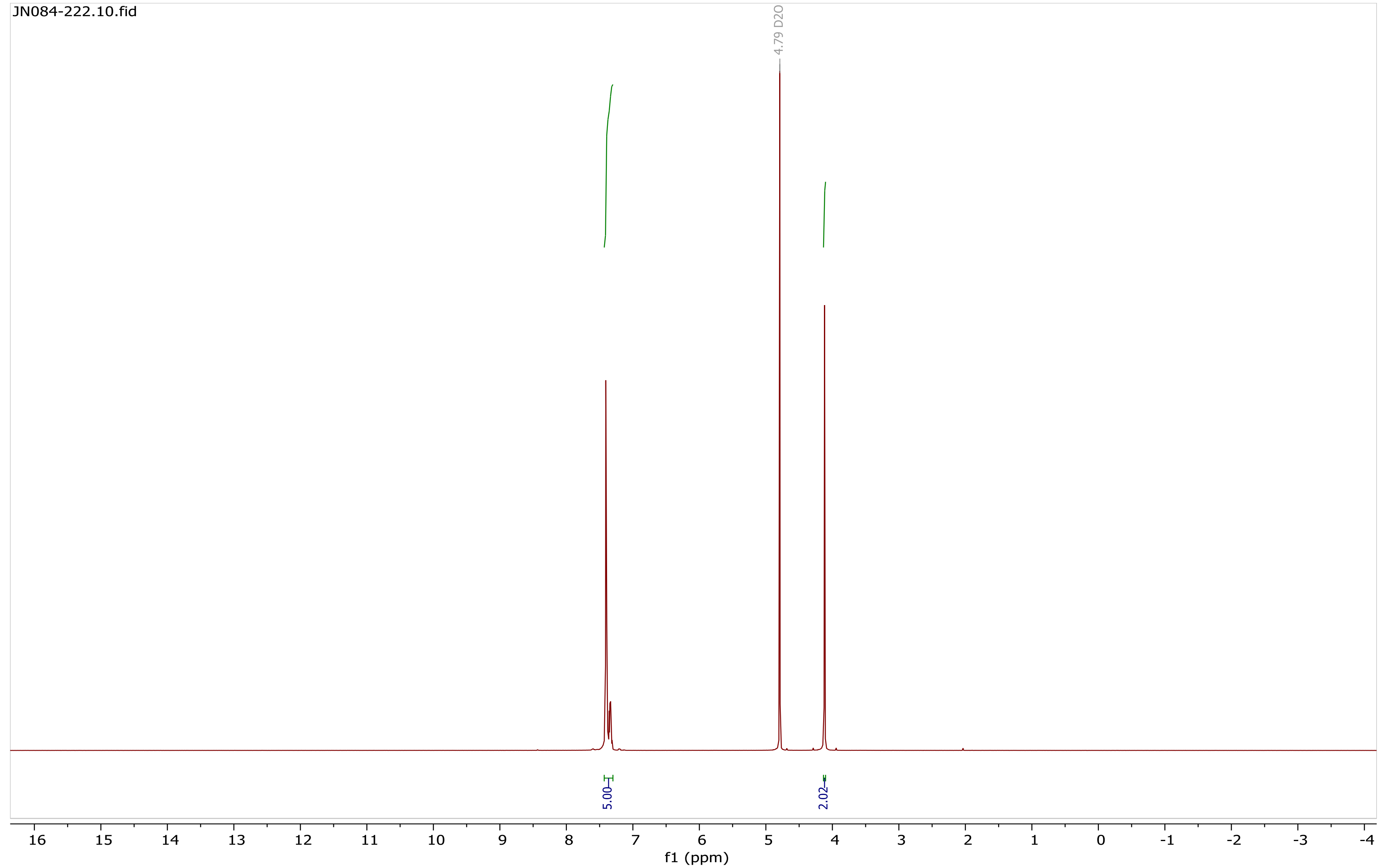

^{13}C NMR spectrum of **8a** (101 MHz, D_2O)

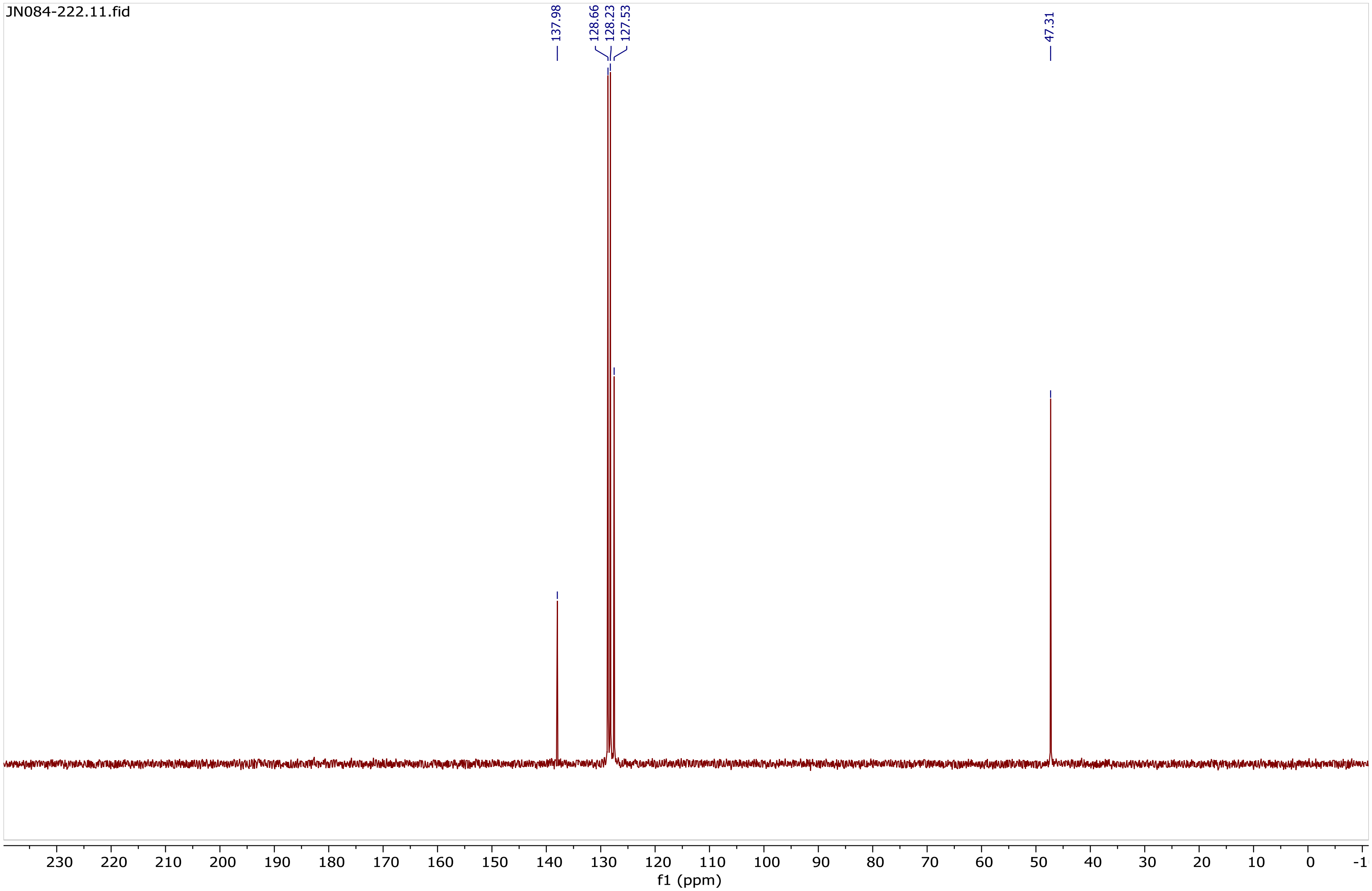

^{1}H NMR spectrum of **8b** (300 MHz, D_2O)

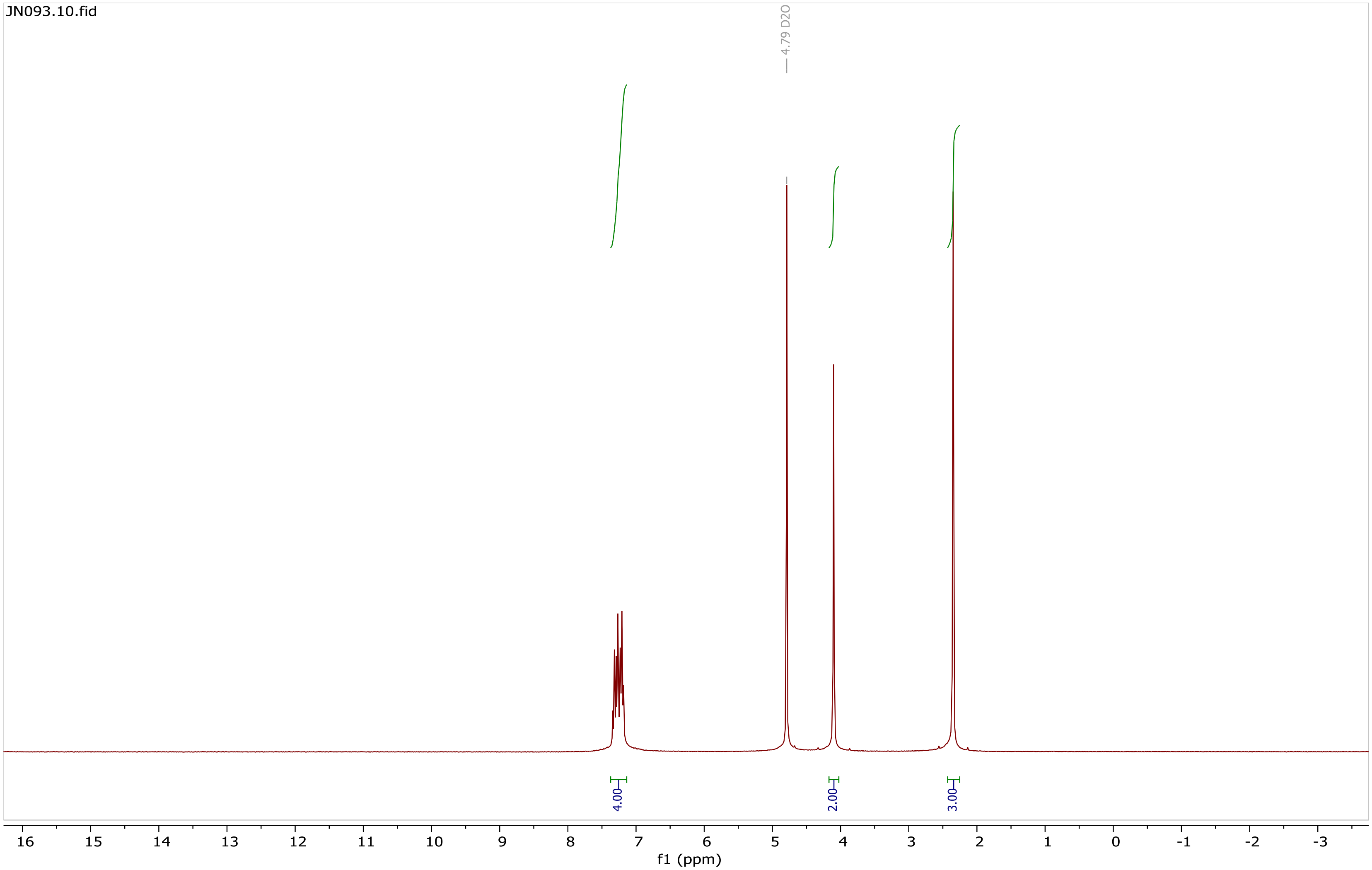

^{13}C NMR spectrum of **8b** (101 MHz, D_2O)

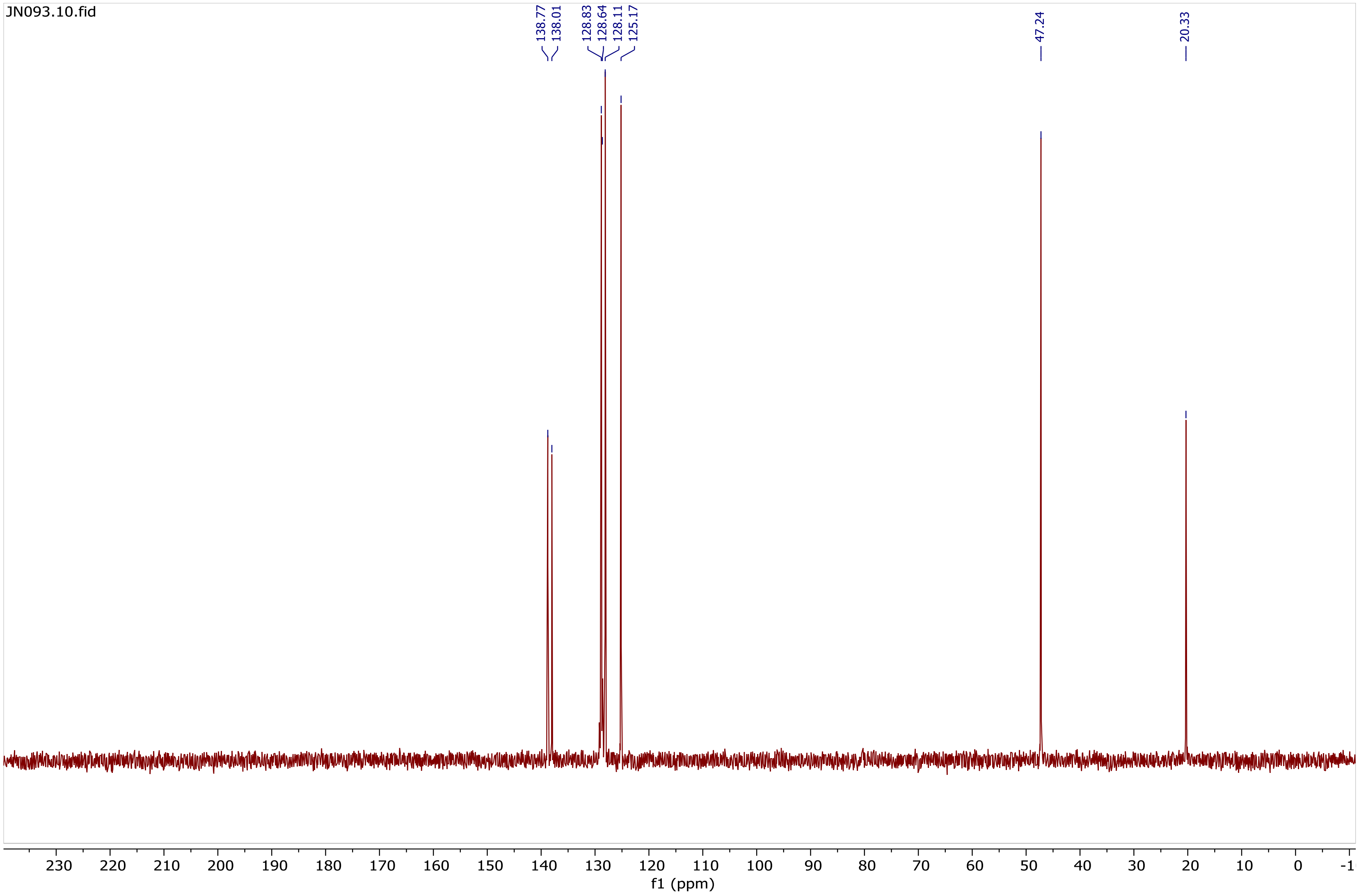

^{1}H NMR spectrum of **8c** (300 MHz, D_2O)

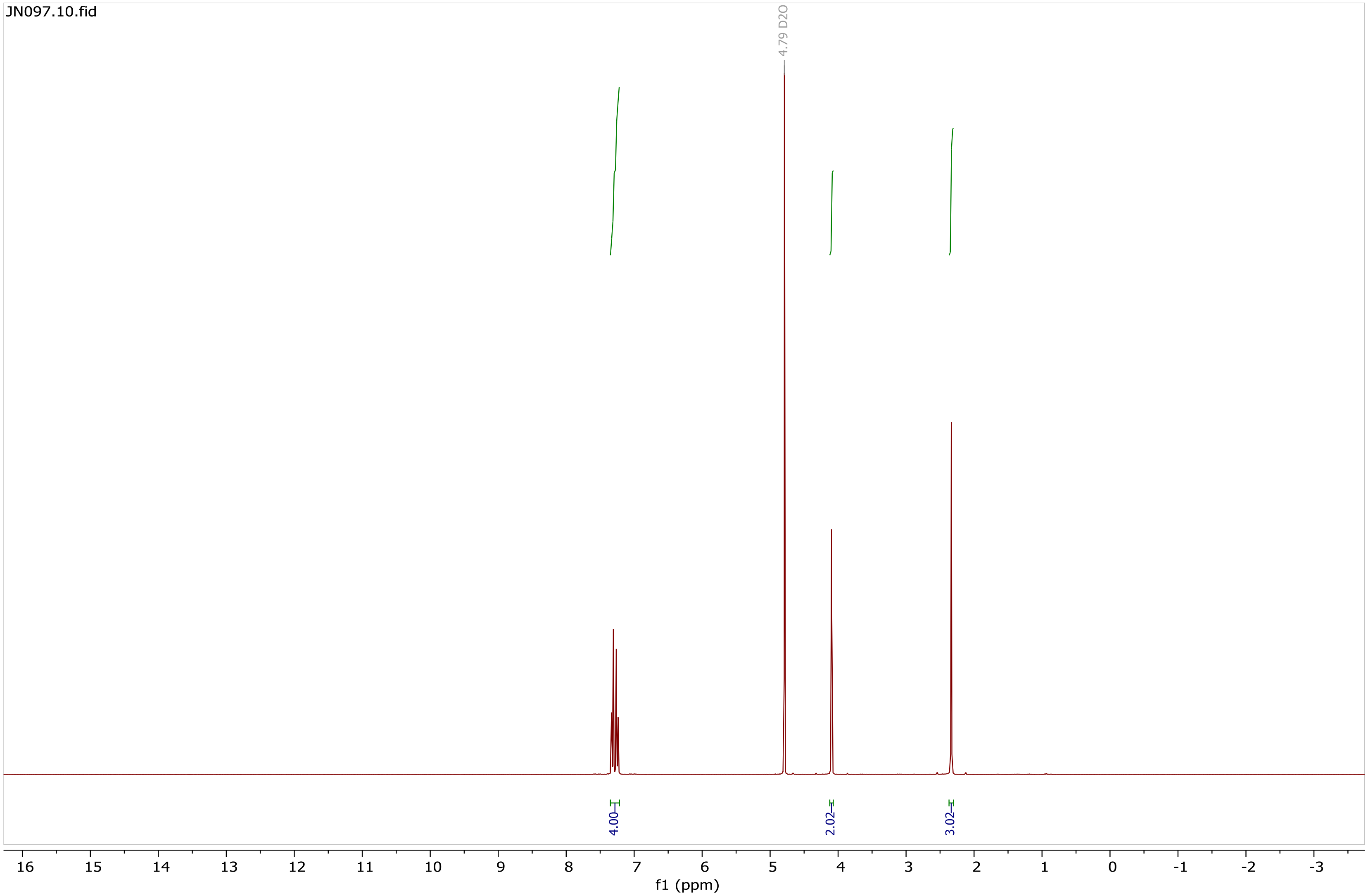

^{13}C NMR spectrum of **8c** (101 MHz, D_2O)

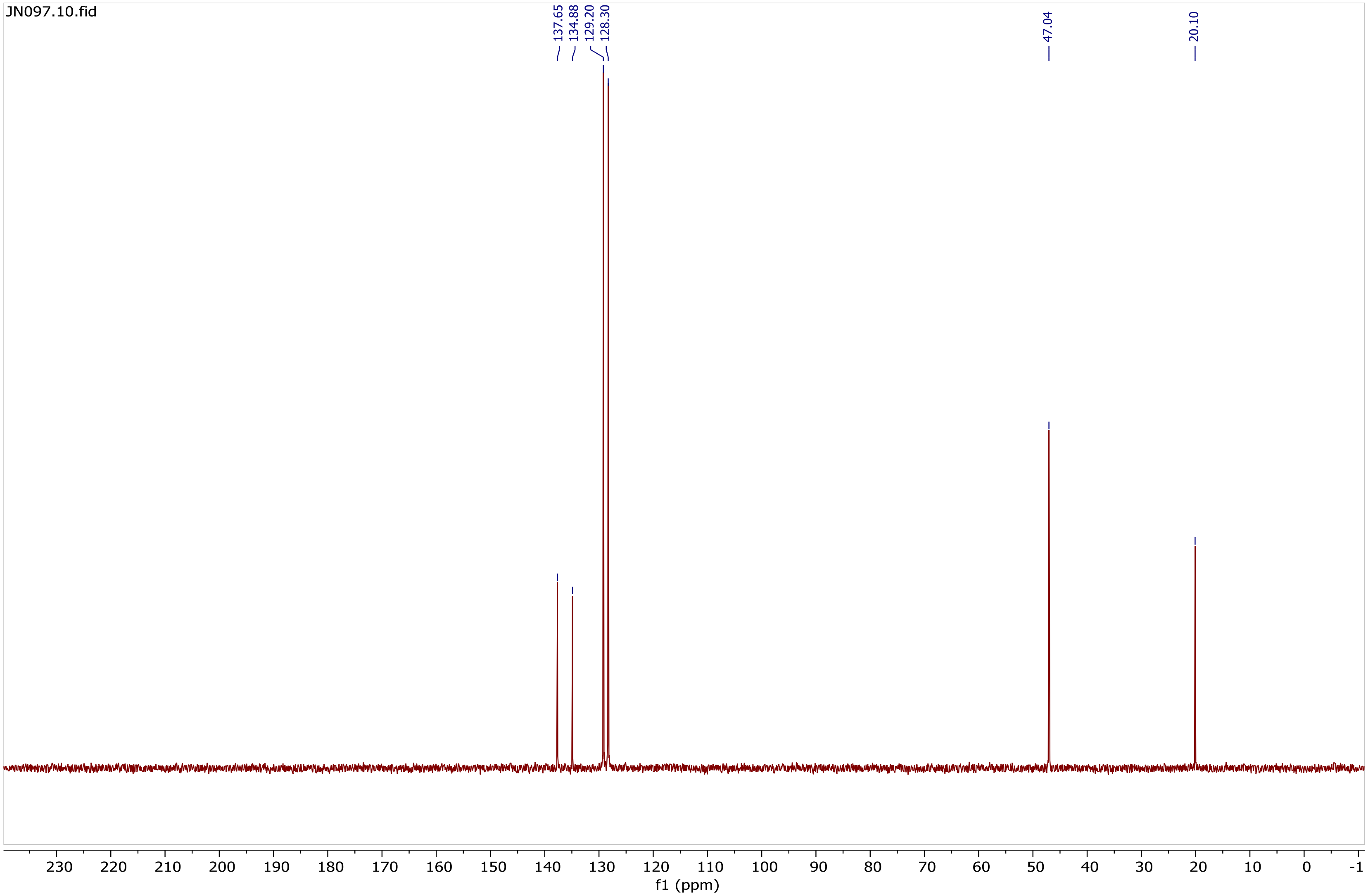

^{1}H NMR spectrum of **8d** (300 MHz, D_2O)

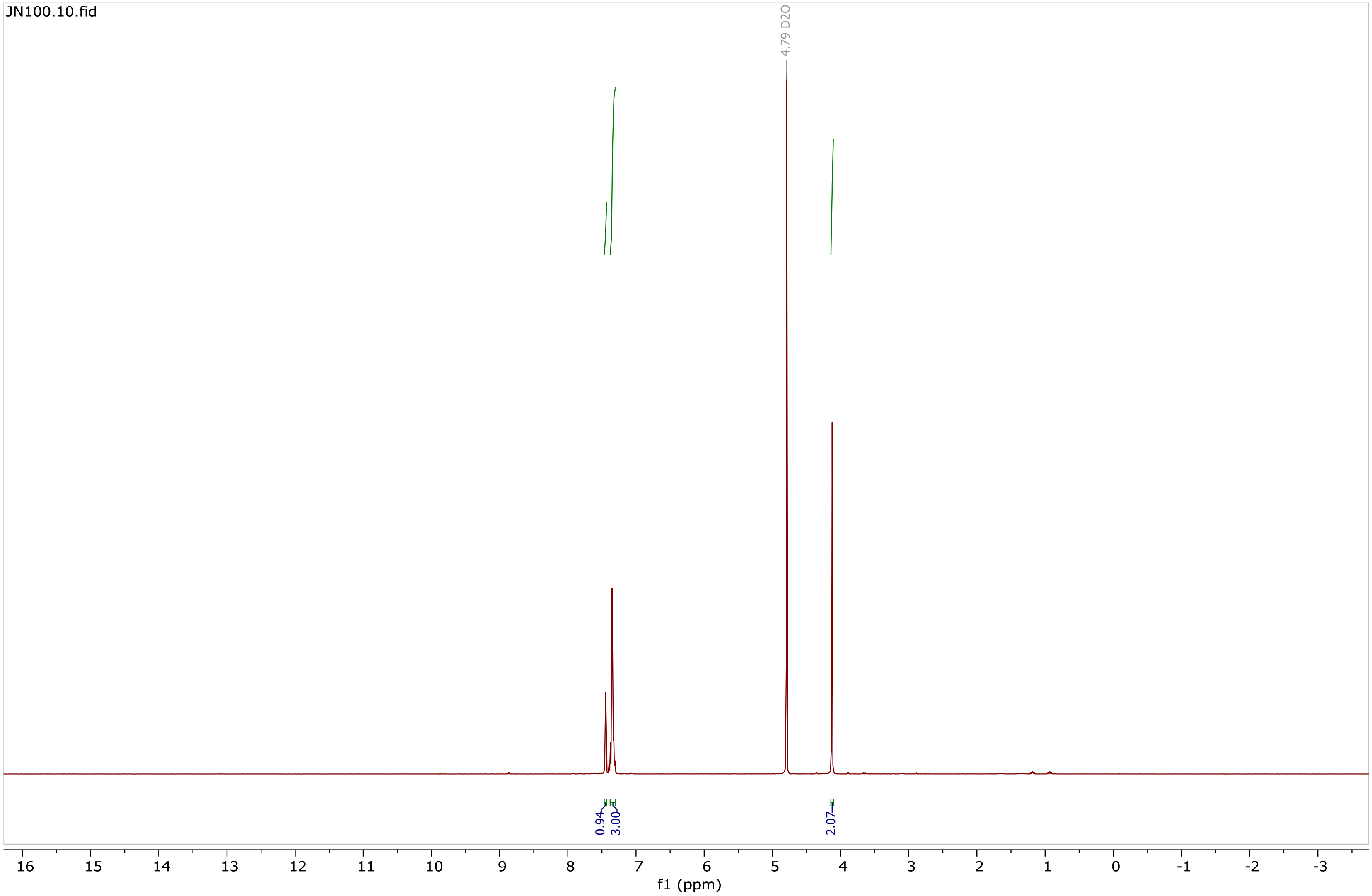

^{13}C NMR spectrum of **8d** (101 MHz, D_2O)

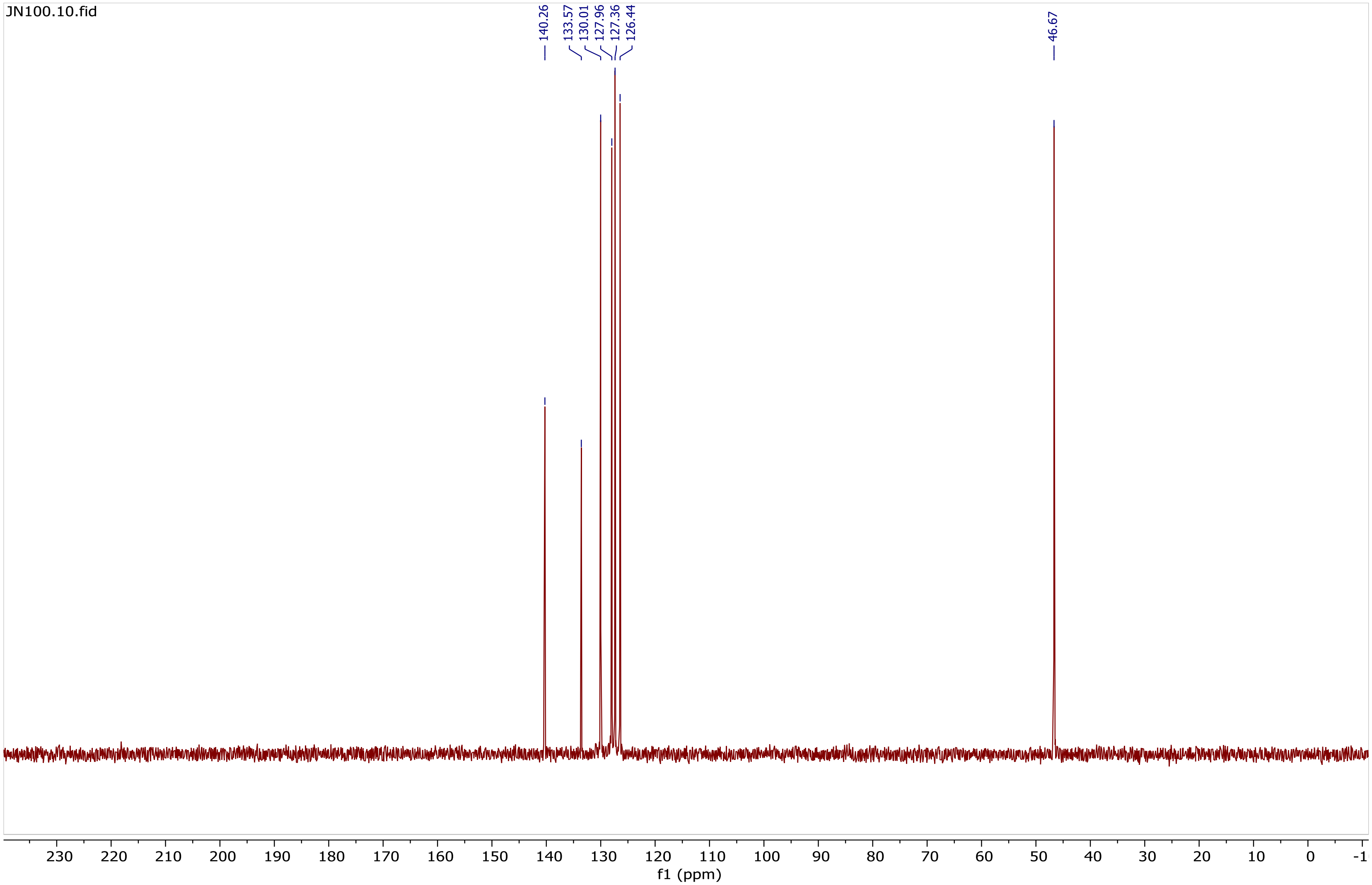

^{1}H NMR spectrum of **8e** (300 MHz, D_2O)

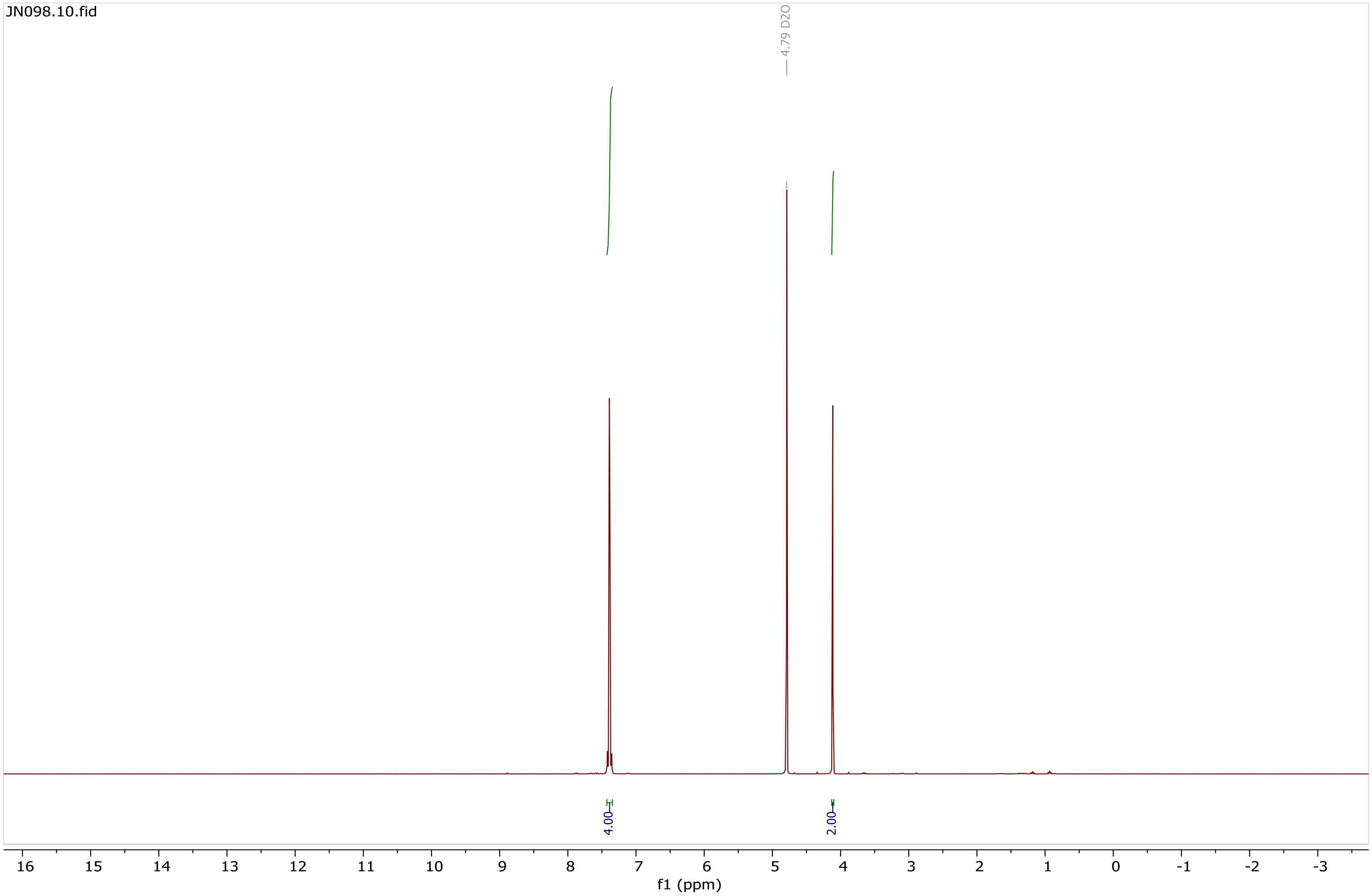

^{13}C NMR spectrum of **8e** (101 MHz, D_2O)

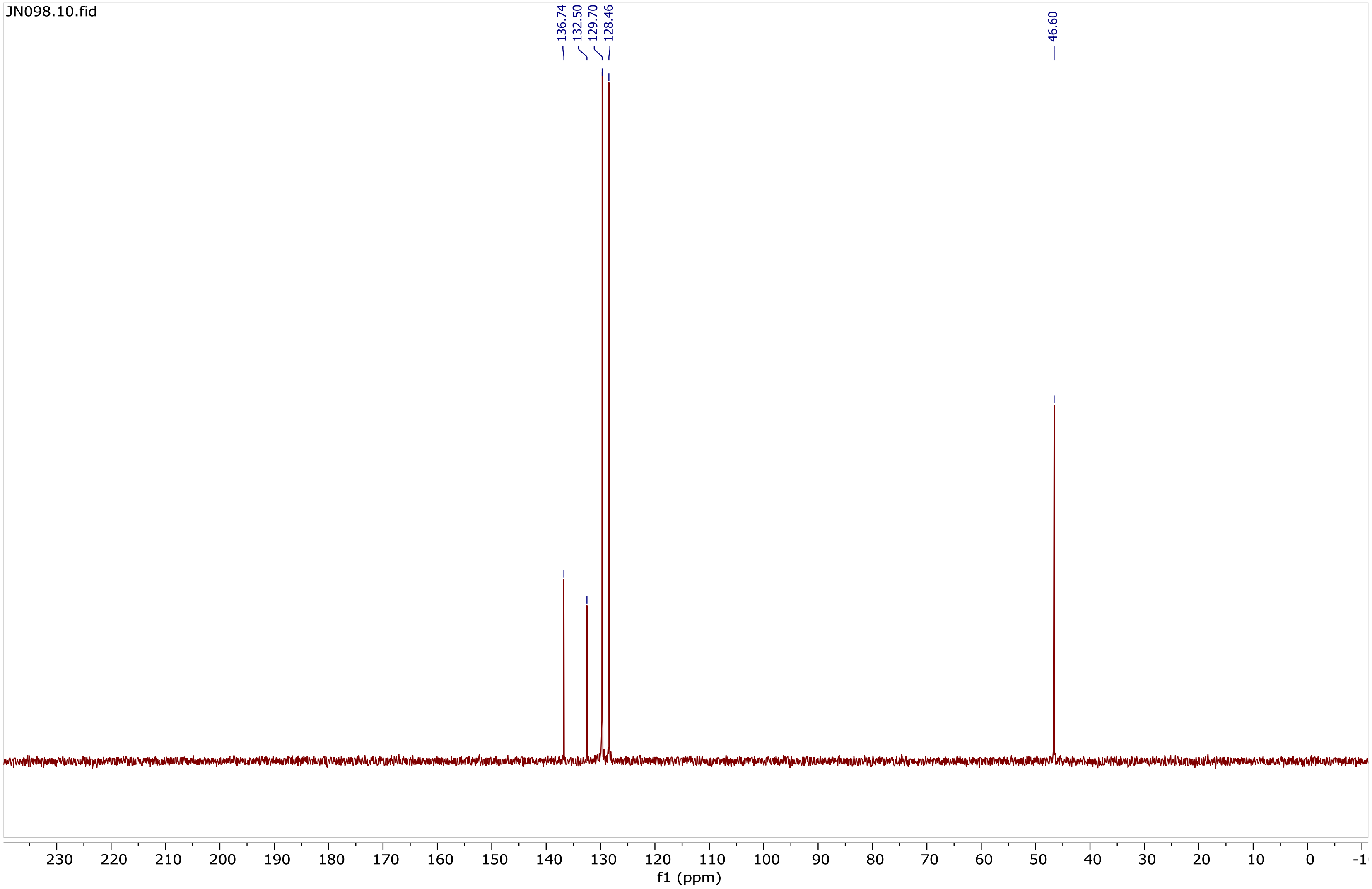

^{1}H NMR spectrum of **8f** (300 MHz, D_2O)

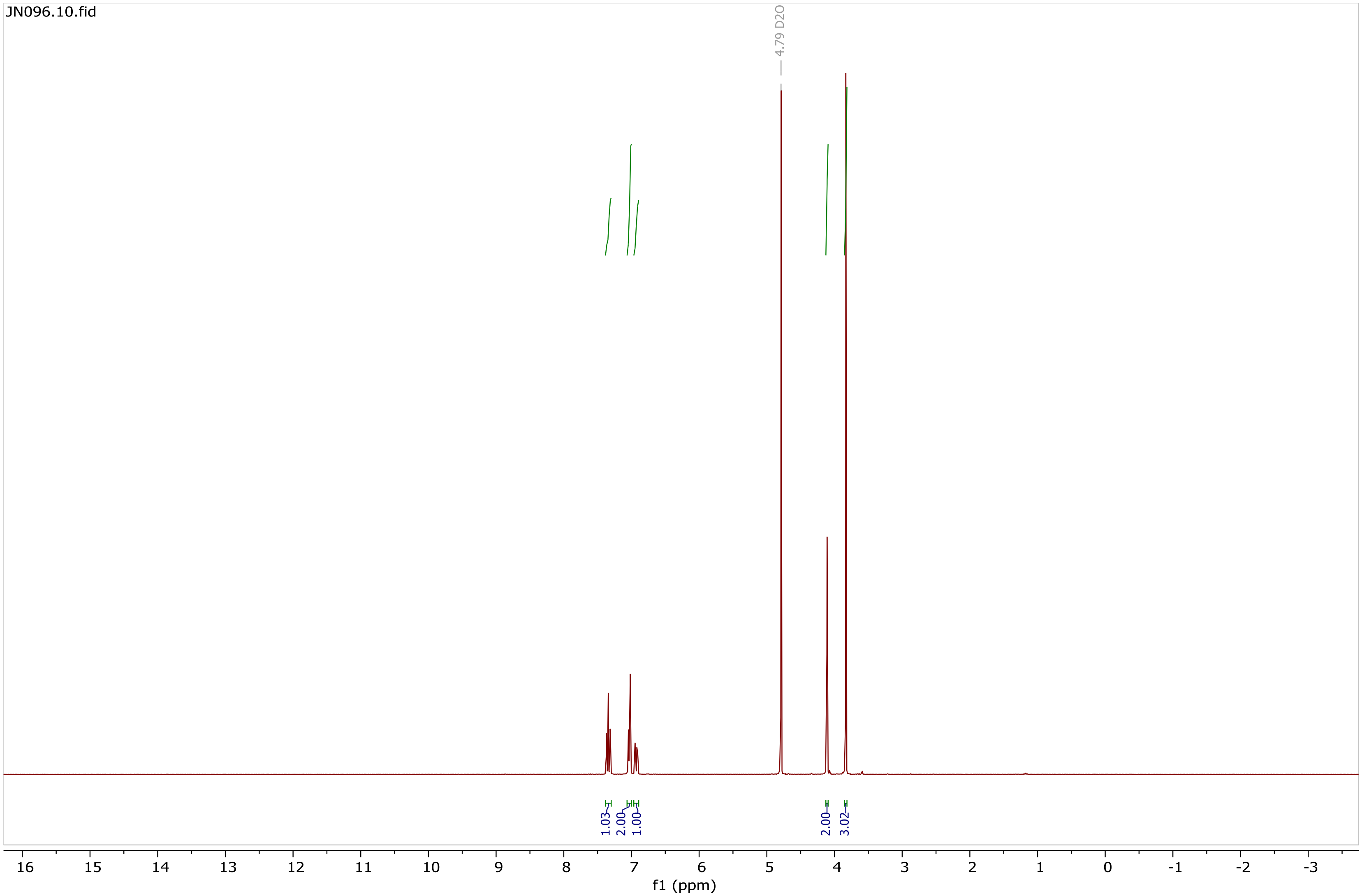

^{13}C NMR spectrum of **8f** (101 MHz, D_2O)

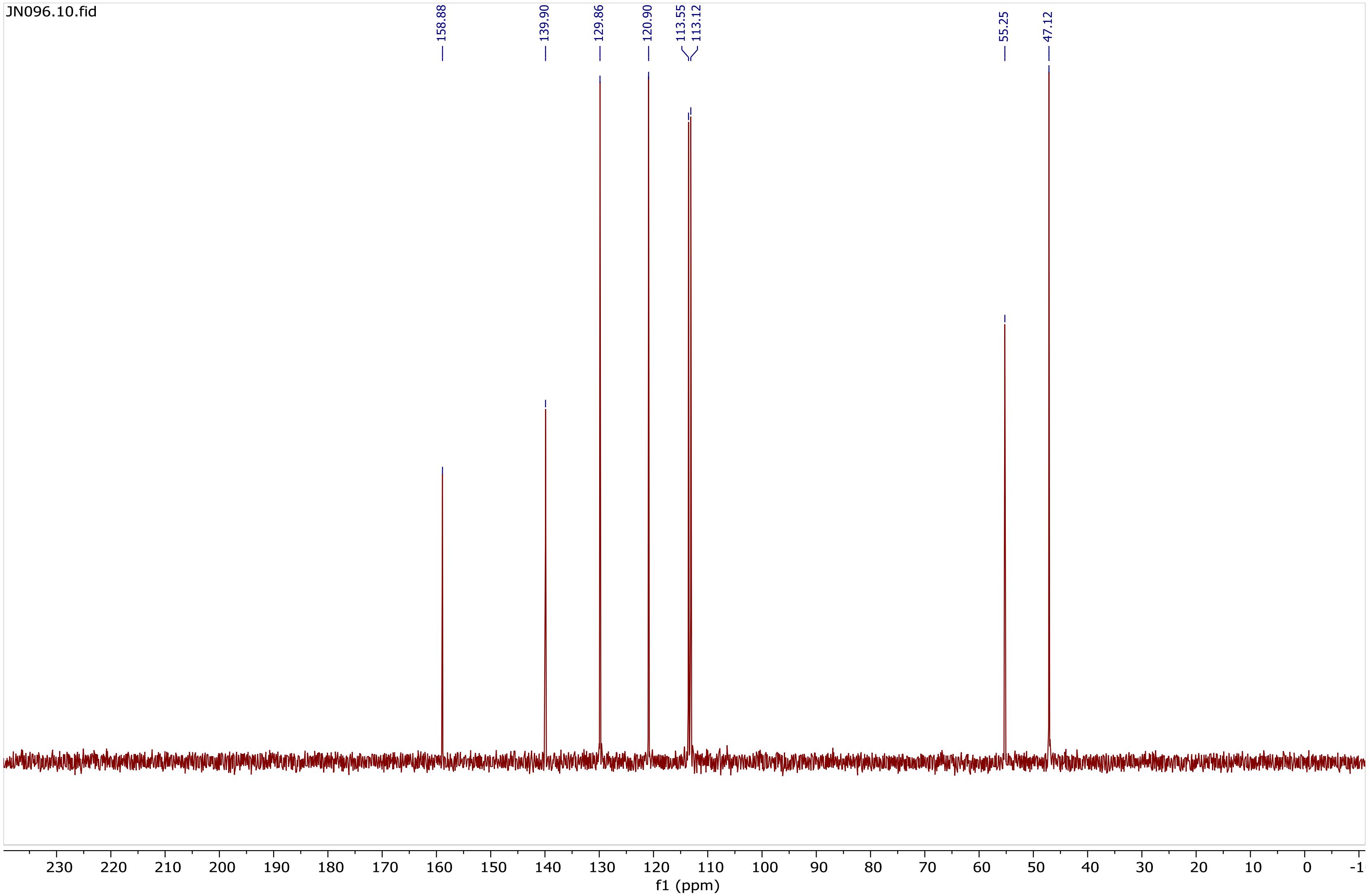

^{1}H NMR spectrum of **8g** (300 MHz, D_2O)

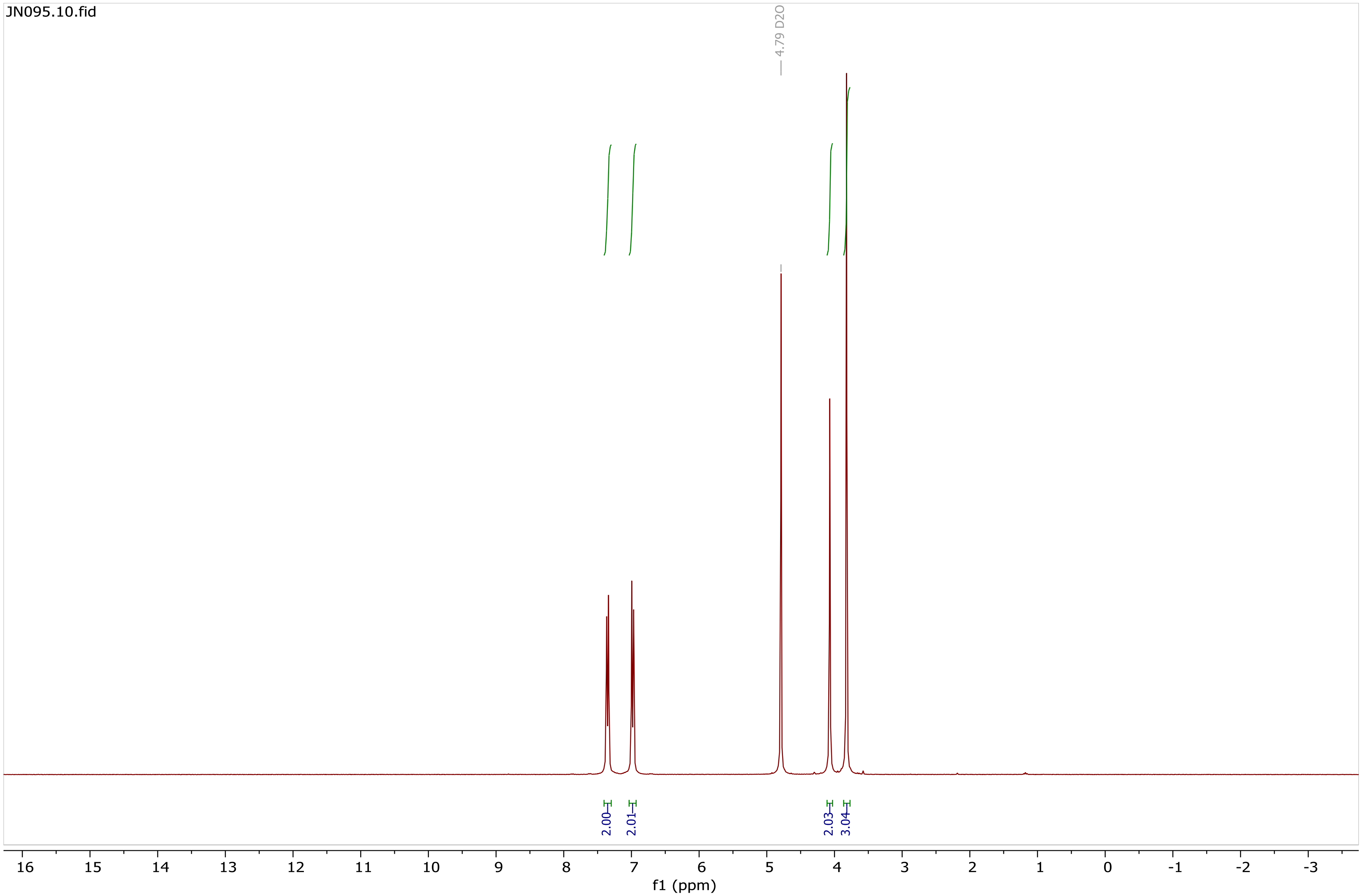

^{13}C NMR spectrum of **8g** (101 MHz, D_2O)

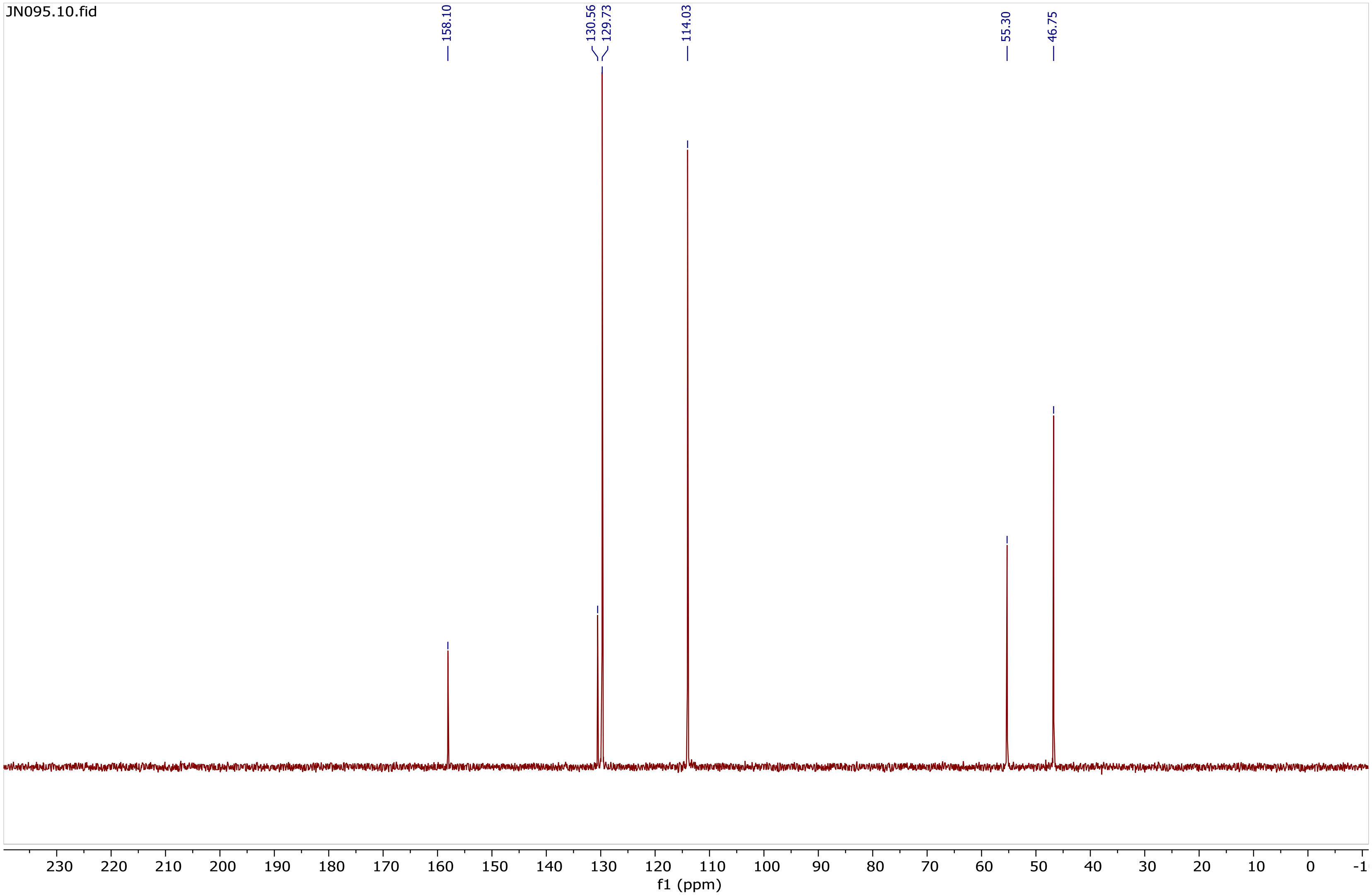

^{1}H NMR spectrum of **9** (400 MHz, $CDCl_3$)

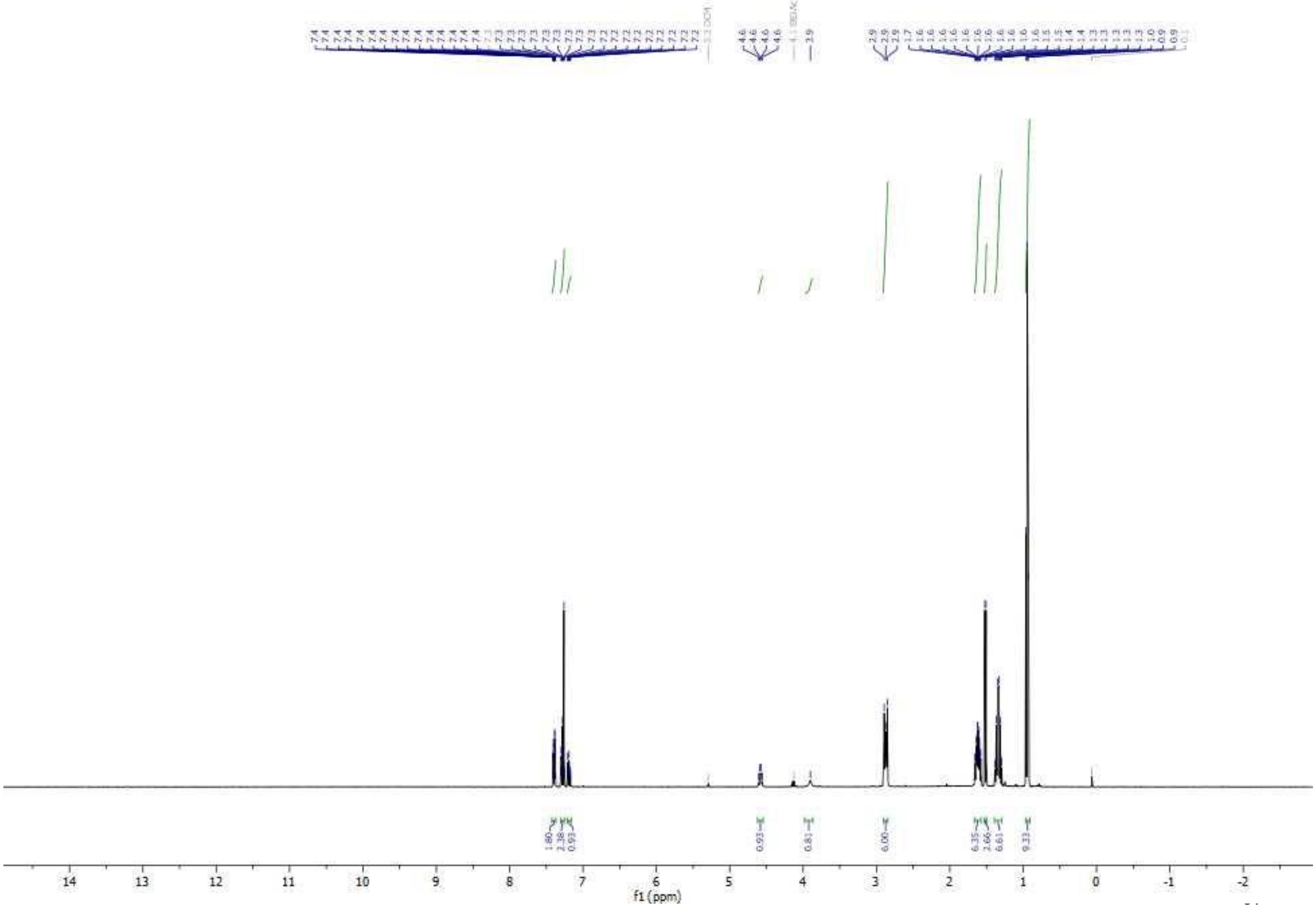

^{13}C NMR spectrum of **9** (101 MHz, $CDCl_3$)

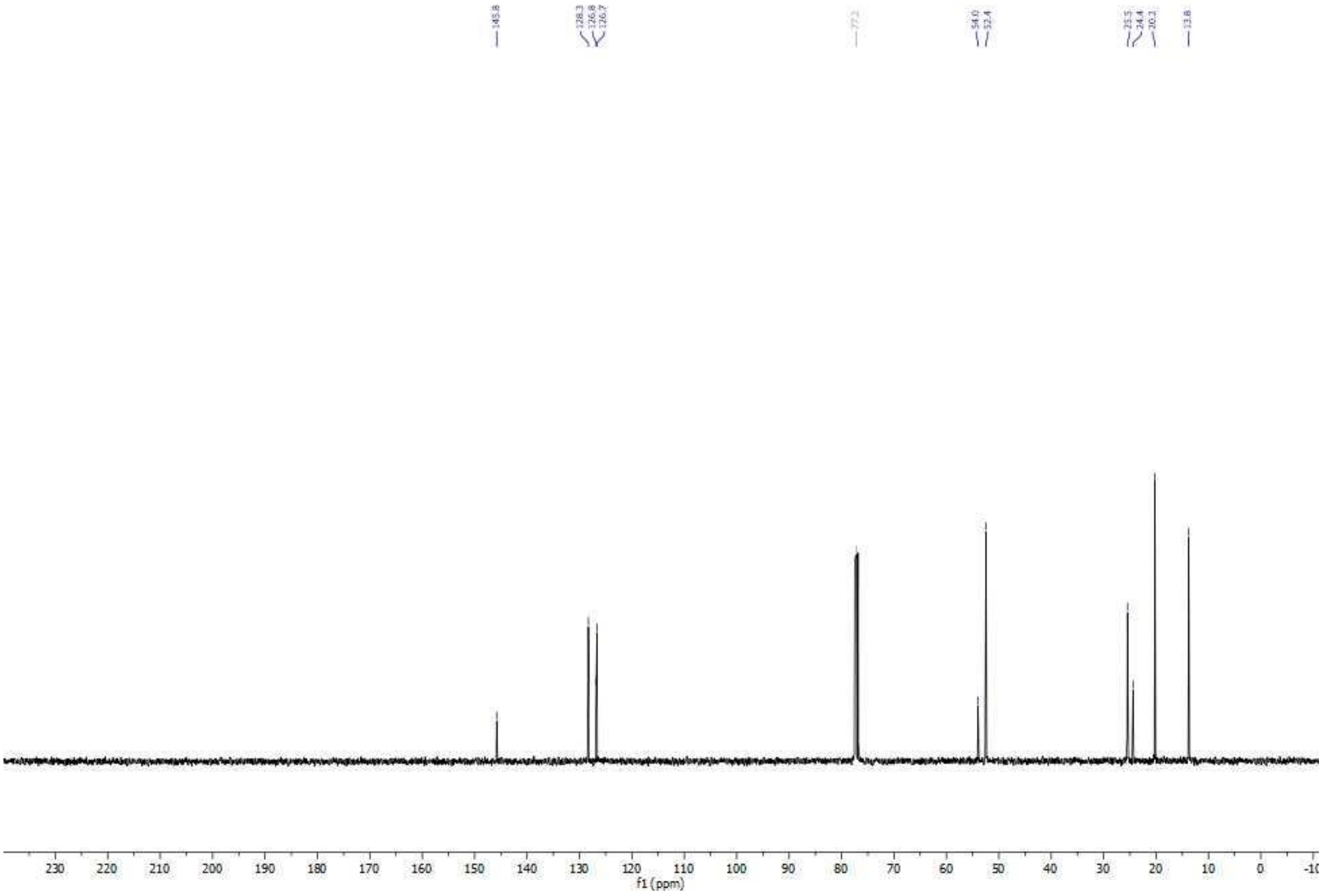

^{1}H NMR spectrum of **10** (400 MHz, $CDCl_3$)

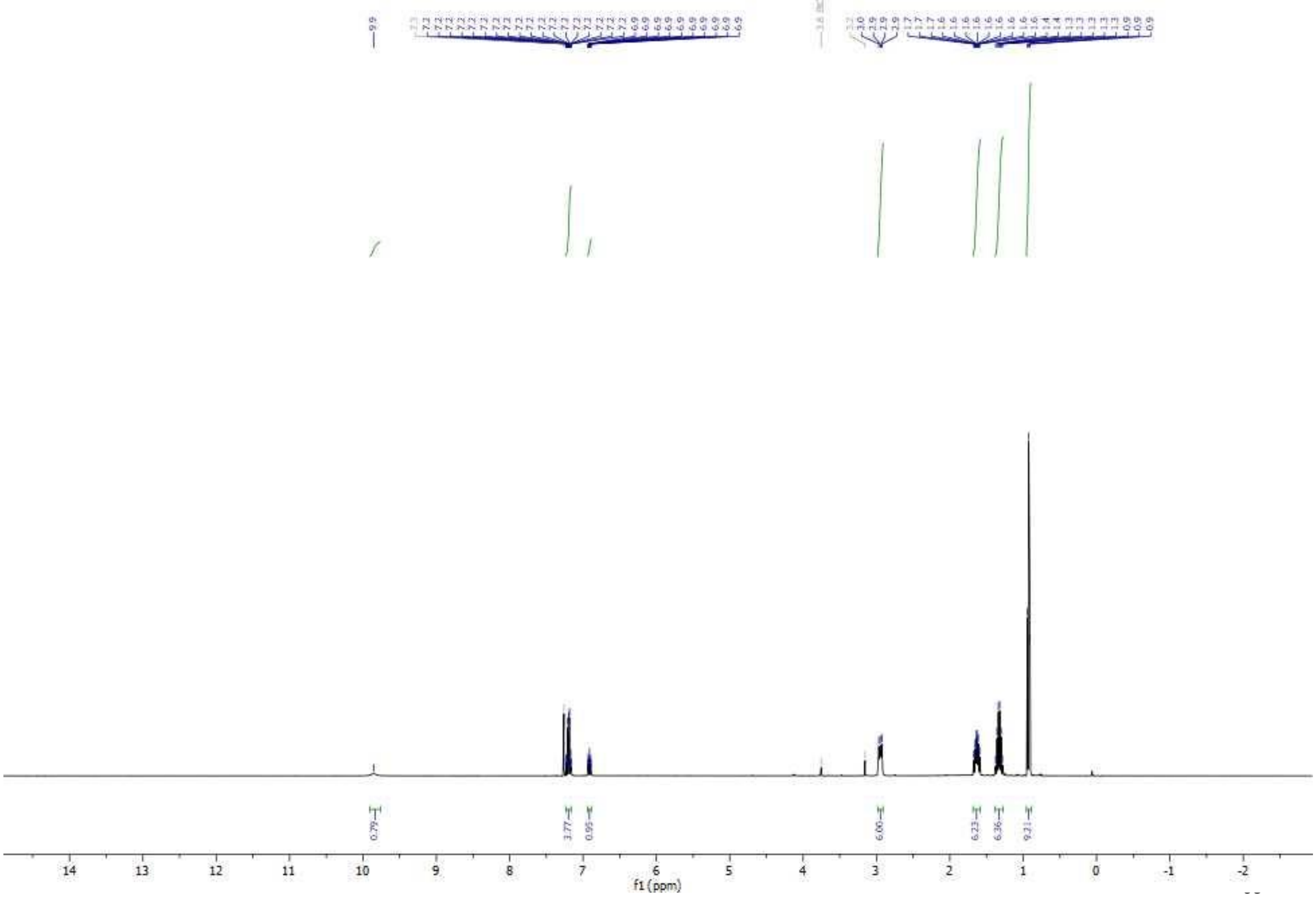

^{13}C NMR spectrum of **10** (101 MHz, $CDCl_3$)

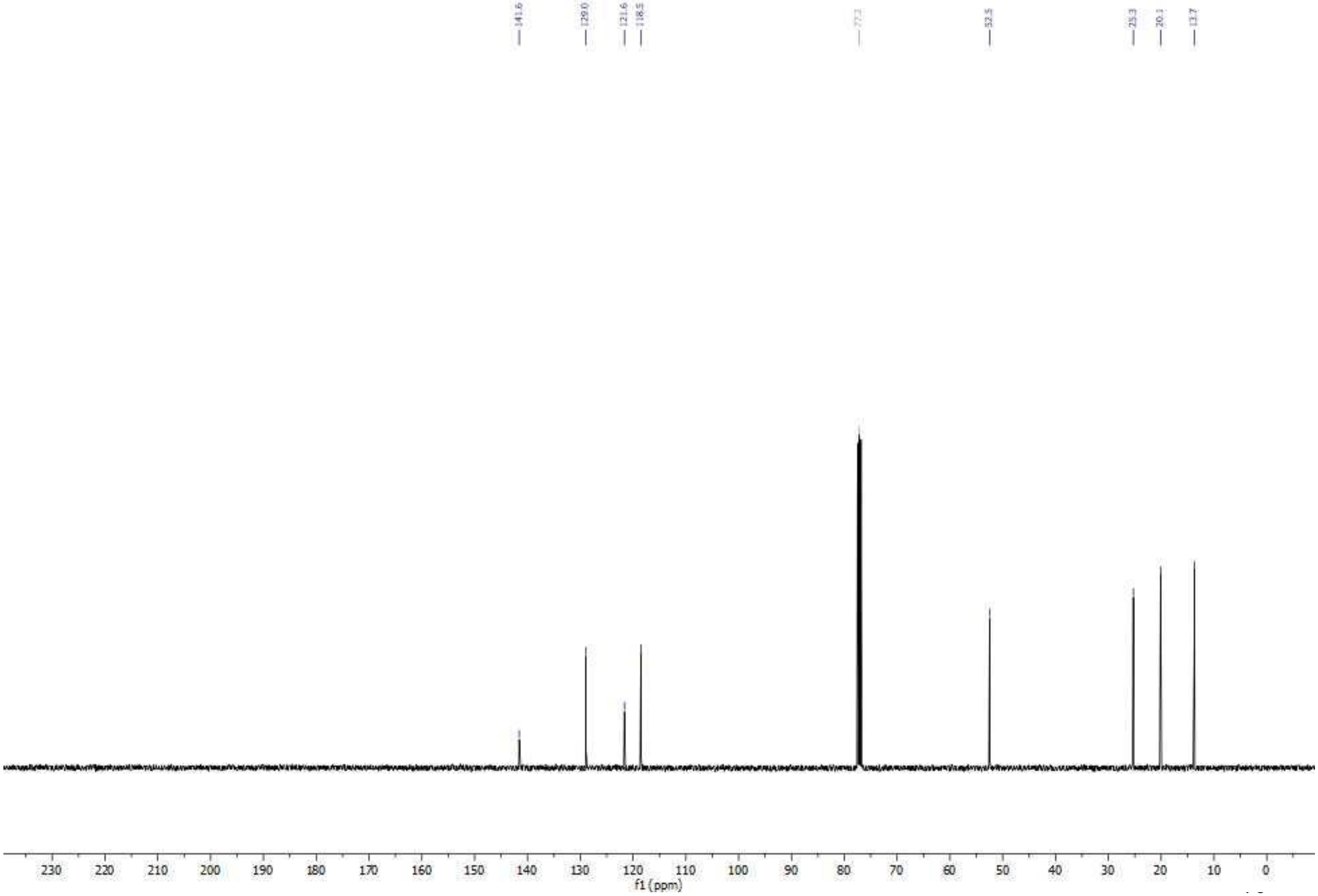

S5. TBSAB control experiment

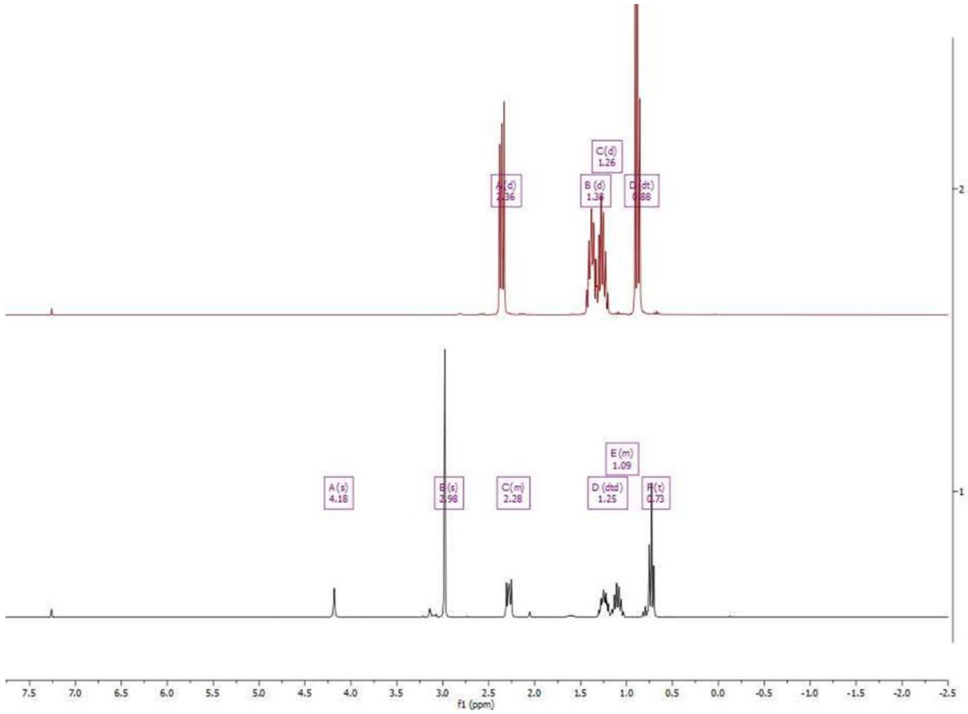

Figure S5. Tributylsulfoammonium betaine cannot be formed *in situ*. (a) ^{1}H NMR spectrum of Bu_3N; (b) ^{1}H NMR spectrum of the reaction mixture of $Me_3N.SO_3$ and Bu_3N in MeCN after 30 min.

S6. References

[1] Gill, D. M., Male, L. and Jones, A. M. Sulfation made simple: a strategy for synthesising sulfated molecules. *Chem. Commun.* **55**, 4319-4322 (2019).

[2] Benedetti, A. M., Gill, D. M., Tsang, C. W. and Jones, A. M. Chemical Methods for N- and O-Sulfation of Small Molecules, Amino Acids and Peptides. *ChemBioChem* **21**, 938-942 (2020).
